# Prevalence and correlates of perinatal depression

**DOI:** 10.1007/s00127-022-02386-9

**Published:** 2023-01-16

**Authors:** Khalood Al-abri, Dawn Edge, Christopher J. Armitage

**Affiliations:** 1 Division of Psychology and Mental Health, University of Manchester, G35 Coupland 1 Building, Manchester, UK; 2 Department of Community and Mental Health, College of Nursing, Sultan Qaboos University, Muscat, Oman; 3 Equality, Diversity and Inclusion Research Unit, Greater Manchester Mental Health NHS Trust, Manchester, UK; 4 Manchester University NHS Foundation Trust, Manchester Academic Health Science Centre, Manchester, UK; 5 NIHR Greater Manchester Patient Safety Translational Research Centre, University of Manchester, Manchester, UK

**Keywords:** Perinatal depression; prevalence, Correlates, Systematic reviews, Meta-analysis

## Abstract

**Purpose:**

This systematic review of systematic reviews aims to provide the first global picture of the prevalence and correlates of perinatal depression, and to explore the commonalities and discrepancies of the literature.

**Methods:**

Seven databases were searched from inception until April 2022. Full-text screening and data extraction were performed independently by two researchers and the AMSTAR tool was used to assess the methodological quality.

**Results:**

128 systematic reviews were included in the analysis. Mean overall prevalence of perinatal depression, antenatal depression and postnatal depression was 26.3%, 28.5% and 27.6%, respectively. Mean prevalence was significantly higher (27.4%; SD = 12.6) in studies using self-reported measures compared with structured interviews (17.0%, SD = 4.5; *d* = 1.0) and among potentially vulnerable populations (32.5%; SD = 16.7, e.g. HIV-infected African women) compared to the general population (24.5%; SD = 8.1; *d* = 0.6). Personal history of mental illness, experiencing stressful life events, lack of social support, lifetime history of abuse, marital conflicts, maternity blues, child care stress, chronic physical health conditions, preeclampsia, gestational diabetes mellitus, being exposed to second-hand smoke and sleep disturbance were among the major correlates of perinatal depression.

**Conclusion:**

Although the included systematic reviews were all of medium–high quality, improvements in the quality of primary research in this area should be encouraged. The standardisation of perinatal depression assessment, diagnosis and measurement, the implementation of longitudinal designs in studies, inclusions of samples that better represent the population and better control of potentially confounding variables are encouraged.

**Supplementary Information:**

The online version contains supplementary material available at 10.1007/s00127-022-02386-9.

## Introduction

Perinatal depression is defined as a non-psychotic depressive episode with various degrees of severity, from mild to major, occurring during pregnancy and up to one year following delivery [1–5], which has been shown to exert harmful effects on both mother and child [6–9]. Mothers may suffer from spontaneous abortion, bleeding during the gestational period, increased resistance of the uterine artery, preterm deliveries, and assisted deliveries, such as Caesarean section (CS), or the use of vaginal instruments [7]. Babies may have lower APGAR (Appearance Pulse Grimace Activity Respiration) scores, increased admission to neonatal care, a higher risk of poor growth development, and increased episodes of diarrheal infection [7, 9]. During the postpartum period, maternal depression has been linked with negative impacts on breastfeeding [3] and mother–infant interactions [1, 10].

Systematic reviews to establish the prevalence and correlates of perinatal depression are an important step in developing effective strategies and interventions, and several systematic reviews have been conducted to date [2, 11–13]. The number of systematic reviews addressing the prevalence and correlates of perinatal depression has been on the rise (a PubMed search found two reviews in the 1990s, 11 reviews in the 2000s, and 95 reviews in the 2010s). Systematic reviews of systematic reviews have been used to provide a broad overview and an up- to -date summary of the research in a particular field, which cannot be provided in more focused systematic reviews, highlighting commonalities and discrepancies, and hopefully identifying new promising approaches [14, 15].

The aim of the present research was to systematically review previous systematic reviews of perinatal depression to provide a global estimate of the prevalence rates and explore potential influences on those prevalence rates, including population characteristics and the methodological quality of the systematic reviews undertaken. Knowledge of the prevalence of perinatal depression and who is at risk will help inform public health policies to prevent and treat perinatal depression. We aimed to answer three research questions:

1. What is the global prevalence of perinatal depression?
2. What are the risk factors associated with developing perinatal depression?
3. What features of the systematic review and/or population under investigation might account for the prevalence and/or correlates of perinatal depression?

## Materials and methods

### Search strategy

**Fig. 1:**
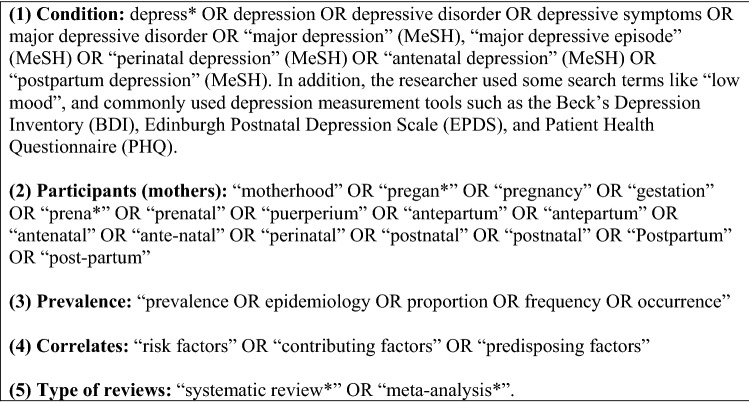
Search terms used (for MeSH terms and keywords in abstract and title)

The review followed guidelines for Preferred Reporting Items for Systematic Reviews and Meta-analyses (PRISMA) [16]. The research questions, inclusion/exclusion criteria and data synthesis approaches were specified in advance and published in PROSPERO (CRD42019135838). We systematically reviewed materials published from the inception of the database until April 2022. The search for systematic reviews was performed using two strategies: (1) systematic searches of electronic databases (Embase, MEDLINE, PsycINFO, CINAHL Plus and Maternity and Infant Care) covering every source related to perinatal depression and grey literature (using PROSPERO and Scopus); and (2) hand searches, searching lists of references for cited systematic reviews. We developed search terms filtered by a combination of different key words and subject medical headings (MeSH), as shown in Fig. 1.

### Inclusion and exclusion criteria

To be considered “systematic”, a review must: (a) describe systematic searches of multiple databases to identify studies that match their research questions; (b) include details of inclusion and exclusion criteria; and (c) make some attempt at data synthesis, including meta-analysis. We included systematic reviews of all cohort, case–control, or cross-sectional studies that investigated and reported the prevalence of perinatal depression or its correlates (risk factors). In addition, all systematic reviews with interventions or experimental studies were included if their control groups provided any outcomes relevant to the prevalence and correlates of perinatal depression. Systematic reviews were included if they assessed the risk factors and prevalence of perinatal depression among women of all ages, ethnicity, country of origin or language spoken. No publication date or language restrictions were applied.

Systematic reviews were excluded if they were duplicates and if they did not report the prevalence of, or factors associated with, perinatal depression.

### Data extraction

The initial search was performed by the first author using pre-determined research terms and strategies for searching databases. All search results were exported to EndNote (version 9) and duplicates were removed. Two reviewers (the first author and the doctoral candidate) then screened all of the titles and abstracts for eligibility, with an agreement level of 99%. Following this, the two reviewers read the full text of potentially relevant systematic reviews to determine whether they met the criteria of eligibility for final inclusion (96% agreement). In cases of disagreement between the reviewers, a third reviewer (a co-author) made the final decision. The main reviewer extracted data according to a pre-designated extraction form. The data extraction form included details about the first author of the systematic review, publication year, title of review, review objective or aims, number of included studies, type of studies, characteristics of population and sample size, countries, time of measurements, type of depression measurements and key findings (prevalence and/or risk factors).

### Assessment of methodological quality

The validated Assessment of Multiple Systematic Reviews (AMSTAR) tool was used to assess the quality of the methodologies in the systematic reviews [17, 18]. AMSTAR contains 11 items with binary scoring; each item has a score of 1 if the criterion is met and 0 if it is not met, is unclear or inapplicable [19]. The total score of the review quality is then calculated by totaling the scores of each item [19]. The AMSTAR score has three levels of review quality: 8–11 indicates high quality, 4–7 indicates medium quality, and 0–3 indicates low quality [19].

## Results

**Fig. 2:**
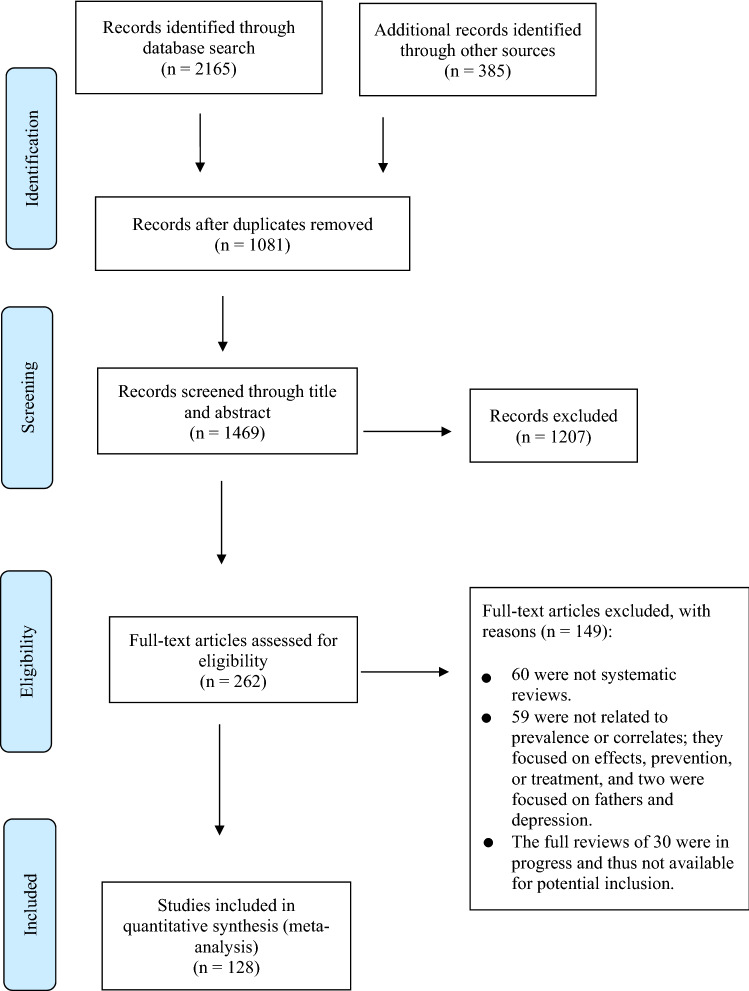
PRISMA diagram of search strategy

The review selection process in this study is presented in Fig. 2 [16]. We obtained 2550 potentially relevant records (comprising 2165 records identified through database searching and 385 identified from other sources, such as grey literature). Of the 2550 identified records, 1081 were duplicates (indexed in more than one database) and were excluded. The remaining 1469 records were included for initial screening of their titles and abstracts, as a result of which 1207 did not meet the inclusion criteria and were excluded. Full-text reviews were then carried out on the remaining 262 reviews, which identified 128 studies that met the inclusion criteria.

### Description of the included systematic reviews

The 128 included systematic reviews described 4242 primary studies, of which 54 (42.2%) reported the prevalence of perinatal depression (supplementary Table 1) and 74 (57.8%) examined risk factors associated with developing perinatal depression (supplementary Table 2). All systematic reviews were examined for features that might account for their apparent discrepancies in the prevalence and/or correlates of perinatal depression.

Seventy-seven (60.2%) of the systematic reviews achieved high quality AMSTAR scores (8–11), while the remaining 51 (39.8%) achieved a medium quality score (4–7) (supplementary Table 3).

### Global prevalence of perinatal depression

The overall mean prevalence of perinatal depression among the included reviews was 26.3%, (SD = 11.6, median = 23.8%). The mean prevalence of antenatal depression (from 11 of the 54 reviews, 22%) was 28.5% (SD = 18.2) versus the mean prevalence of postnatal depression (from 24 of the 54 reviews, 44%) which was 27.6% (SD = 8.0). The effect size for the difference was *d* = 0.1, which is considered a small effect [20].

### Prevalence of difference between using self-reported measures and diagnostic assessment

There was a difference between the mean perinatal prevalence of depression identified using self-reported measures (27.4%, SD = 12.6) compared to those identified using a structured interview (17.0%, SD = 4.5; *d* = 1.0) and those identified using both self-reported measures and structured interviews (25.7%, SD = 9.0; *d* = 0.8). Out of the included systematic reviews, 48.1% were based only on self-reported measures, 50.0% were based on both self-reported and diagnostic assessments, and 1.9% of the included systematic reviews were based only on diagnostic assessment.

### Prevalence in potentially vulnerable populations

The prevalence of perinatal depression was markedly higher (32.5%; SD = 16.7) among potentially vulnerable populations, such as immigrants [21–25], HIV-infected African women [26], women who gave birth prematurely or whose infants had a very low birth weight [27], mothers who abused substances during pregnancy [28], military personnel [29], women who had experienced earthquakes [30, 31] and pregnant inmates in correctional facilities [32], compared to the general population (excluding data from vulnerable groups), with an estimated mean prevalence of 24.5% (SD = 8.1; *d* = 0.6). This may indicate that potentially vulnerable populations are at a higher risk of developing perinatal depression than the general population.

The Prevalence of depression varied widely (SD = 16.7, Table 1), which was largely attributable to Mukherjee et al. [32], who reported 80% prevalence among pregnant inmates in their last trimester. Mukherjee et al. [32] interpreted their findings as being due to an absence of satisfactory medical care, isolation, stress, anxiety, maternal role transition, and parenting worries. This review uniquely considered depression among incarcerated pregnant women. Further research is required to address mental health issues among what might be a particularly vulnerable group.

**Table 1:** The mean prevalence of perinatal depression among vulnerable groups

No	First author	Publication year	Number of studies	Sample size	Vulnerable population	Mean prevalence (%)
1	Alhasanat	2015	26	Ranged from 60 to 2,137	Arabic immigrant women	35.6
2	Anderson	2017	53	Total of 20,263 mothers	Immigrant women	28.5
3	Falah Hassani	2015	24	Ranged from 61 to 24,455	Immigrant women	20.0
4	Fellmeth	2017	40	Ranged from 31 to 909 (19, 349)	Immigrant women originating from LMICs	24.35
5	Nilaweera	2014	15	Ranged from 58 to 54,594 (102,427)	South Asian immigrant women	12.5
6	Mukherjee	2014	3	Ranged from 25 to 1,213	Women in correctional facilities	80
7	Khatri	2019	7	Ranged from 99 to 670 women	Experiencing earthquake	22.7
8	Ren	2014	8	Ranged from 29 to 1545	Post-earthquake population	33.4
9	Vigod	2010	26	Total of 2392 mothers	Preterm and low‐birth‐weight infants	40
10	Sowa, NA	2015	22	Ranged from 70 to 1922	HIV-infected African women	33
11	Chapman	2013	6	Ranged from 24 to 595	Substance abuse during pregnancy	32.85
12	Klaman	2016	10	Ranged from 82 to 3956	Military women	27.65

*LMICs*  lower- and middle-income countries

### Prevalence of perinatal depression during the COVID-19 pandemic

The overall mean prevalence of perinatal depression among the included three updated systematic reviews and meta-analyses [33–35], with a total of 43 studies evaluating perinatal depression during the COVID-19 pandemic, was 28% (SD = 6).

### Factors associated with perinatal depression

A total of 2123 studies reported correlates of perinatal depression in 74 systematic reviews. The correlates were routinely classified as ‘major’ or ‘minor’.

### Major correlates

Major correlates of perinatal depression were defined as statistically significant predictors in multivariable/logistic regression analyses or with a medium-to-large significant effect size (*rs* > 0.30). Other correlates, which were classed as minor or inconsistent, can be found in supplementary Table 2.

Medium to large significant effect sizes were reported in meta-analyses between perinatal depression and the following risk factors: personal history of mental illness (e.g., anxiety, depression, etc.), with mean *r* ranging from 0.30 to 0.51; childcare stress (*r* = 0.48–0.49) or infant temperament (0.33–0.34); experiencing stressful life events (*r* = 0.36–0.40); lack of social support (*r* = 0.37–0.45); maternity blues (*r* = 0.35–0.37); and marital conflicts or dissatisfaction (*r* = 0.37–0.39) [6, 11, 36, 37].

Lifetime history of abuse (e.g., childhood/adult abuse, maternal violence, or intimate partner violence) was found to conclusively and consistently lead to a developed risk of perinatal depression, sourced from nine systematic reviews and five meta-analyses [36–49]. Women who had experienced intimate partner violence during pregnancy had increased odds of both antenatal and postnatal depression, 1.69–3.76 and 1.46–7.04, respectively [41]. A history of abuse was significantly associated with antenatal depression, with moderate effect sizes for: any abuse (*r* = 0.29; 95% CI = 0.22–0.35; *P* < 0.001); physical abuse (*r* = 0.27; 95% CI = 0.24–0.30; *P* < 0.001); sexual abuse (*r* = 0.26; 95% CI = 0.22–0.30; *P* < 0.001); and emotional abuse (r = 0.34; 95% CI = 0.23–0.44; *P* < 0.001) [46]. A recent meta-analysis found that women who had experienced intimate partner violence had a 5.46-fold increased risk of postnatal depression (OR = 5.46 (95%CI: 3.94- 7.56) [36]. Another meta-analysis revealed that women who had experienced maternal violence were noted to be at a higher risk of postnatal depression (OR = 2.04; 95% CI 1.72–2.41) [49]. This risk was increased in women with a history of having experienced sexual violence (OR = 1.56; 95% CI 1.35–1.81), emotional violence (OR = 1.75; 95% CI 1.61–1.89), physical violence (OR = 1.90; 95% CI 1.36–2.67), and domestic violence (OR = 2.05; 95% CI 1.50–2.80) or childhood violence (OR = 1.59; 95% CI 1.34–1.88) [49].

In three of the included meta-analyses, gestational diabetes mellitus (GDM) was reported to lead to a significantly increased risk of postnatal depression (relative risk (RR) = 1.32, 95% CI 1.09–1.60, *P* = 0.001) [50] (RR = 1.59 (95% CI 1.22–2.07, *P* = 0.001) [51] compared to women without GDM. This finding was echoed by a US-based systematic review, which noted a strong relationship between GDM and postnatal depression among ethnically diverse women from LICs [52].

Chronic medical conditions were at increased risk for perinatal depression (adjusted pooled odds ratios [aPOR] = 1.45; 95% CI 1.25–1.67), specifically: diabetes (aPOR = 1.34; 95% CI 1.07–1.69); hypertension/heart disease (aPOR = 1.60; 95% CI 1.05–2.45); migraine (aPOR = 1.75; 95% CI 1.20–2.54); and other neurological disorders (aPOR = 1.45; 95% CI 1.19–1.77) [53]. The risk of perinatal depression was also noted to increase in severity among women with preeclampsia and preterm deliveries (severity score of 2.7) compared to healthy preterm mothers (severity score of 1.2) [54].

Other factors, such as exposure to second-hand smoke, also significantly increased the risk of developing perinatal depression (ORs = 1.77; 95% CI = 1.12–2.79; *P* = 0.01) [55]. Sleep disturbance as a predictor of postnatal depression was examined in one systematic review [56], with effect size being used as an indicator for the strength of the relationship between postnatal depression and postpartum sleep disturbance, categorised as: small (0.2–0.4), medium (0.5–0.7), large (0.8–1) and very large (1.1–2). That particular systematic review showed that the effect sizes ranged from moderate to large and very large (0.6–1.7), indicating a strong relationship between sleep disturbance and postnatal depression among its included studies, bar one study with a small effect size (0.4) [56].

## Discussion

To the best of our knowledge, this is the first systematic review of systematic reviews focusing on perinatal depression and its correlates, providing an overall, global viewpoint. The primary aim of this study was to estimate the mean global prevalence of perinatal depression, which was found to be 26.3% (SD = 11.6) with a median prevalence of 24.3%. Notably, the absolute difference in perinatal depression prevalence estimates between antenatal (28.5%) and postnatal (27.6%) depression, though significant, was relatively small. Evidence shows that antenatal depression leads to an increased risk of developing postnatal depression [3, 57]. Therefore, early screening for depressive symptoms and diagnosis by an allied health professional during pregnancy is crucial to prevent postnatal depression, by providing early intervention and support. Perinatal depression rates were also noted to be higher among potentially vulnerable populations compared to the general population, thus developing protocols for the early identification and management of perinatal depression is essential for vulnerable groups.

Another aim of this study was to examine the correlates of risk factors for developing perinatal depression. Medium to large significant effect sizes were reported in meta-analyses between perinatal depression and the following of factors: personal history of mental illness (e.g., anxiety, depression, etc.); child care stress or infant temperament; experiencing stressful life events; lack of social support; maternity blues; marital conflicts or dissatisfaction and history of lifetime abuse (e.g., childhood/adult abuse, maternal violence, or intimate partner violence).

The third aim of this study was to report on the features of the included systematic reviews; variations in how the included systematic reviews were conducted could have potentially affected the findings of this systematic review of systematic reviews. Notably, the systematic reviews were restricted to a local community or geographical region [58–60]. Of the 128 included systematic reviews, 54 reported the prevalence of perinatal depression, 39 (72.2%) of which were country-specific, and only 10 (18.5%) reported the prevalence across more than one country. Populations present in a specific country may not be necessarily representative of the general population across other countries and there are even differences within countries due to a variety of differences, such as ethnicity and culture. There is currently no evidence of how these variations could potentially affect the findings, and so primary, cross-cultural research is needed to help understand this issue.

Various risk factors, such as exposure to second-hand smoke [55], GDM

[50, 51, 61] and sleep disturbance [62] were found to significantly increase the risk of postnatal depression; however, these risk factors were examined during the postnatal period and there was a lack of evidence regarding their association with antenatal depression. Therefore, separating the assessment of risk factors of both antenatal and postnatal depression is an important first step in clarifying the cause and effects among the many potential risk factors. Additionally, periodic assessment of the risk factors is needed as these correlates, such as social support and experiencing stressful life events, may fluctuate over time. Further research is needed to investigate these correlates both after delivery and during the pregnancy.

We also observed that each systematic review used different time periods to assess or identify the symptoms of depression. For example, some studies measured depression in the first trimester of pregnancy [63], while others measured depression during the pregnancy or 6 months after the birth, with some even including participants up to 1 year postnatally [1]. The time period during which the research is being carried out should be assessed more thoroughly prior to the start of the study, there is a need for further epidemiology studies to confirm prevalence rates of depression at different time points, as well as the need to confirm which specific time point(s) would allow for the best rate of diagnosis of depressed women [64].

Another noted discrepancy among the included systematic reviews was the assessment method used to identify depressive symptoms, there was a larger number of reviews using self-reported measures as compared to diagnostic assessment. This might have affected the estimates of perinatal depression due to self-reported measures potentially overestimating the findings. It was also observed that the self-reported measures among the included systematic reviews used different cut-off points for the indication of any depressive disorder or major depressive disorder: different Edinburgh Postnatal Depression Scale cut-off points of 9/10 (or more) to 12/13 (or more) out of 30; Beck’s Depression Inventory with 15 or 16 (or more) out of 63; and CESD between 16 (or more) to 24 (or more) out of 60. Despite this variation, there was no clear correlation between cut-off points and prevalence estimates for either measurement tool, suggesting that using lower scores may increase the number of false positives but may not necessarily increase the estimate.

Another feature among the systematic reviews was a lack of, or limited, adjustment for confounding factors which may affect the consistency of some of the risk factors of perinatal depression. For example, there were inconsistent findings in the relationship between CS and developing perinatal depression [65–68]. This may be explained due to the fact that CS seems to present as a risk factor only if women are vulnerable to perinatal depression, brought on by another factor, such as lack of experience with children and/or a negative birth experience. The impact of CS on maternal mood may also depend on the context in which the CS arose (e.g., cultural rules, poor mental health, previous negative birth experiences, preparedness and the social support available to the mother) [65, 67].

Although the systematic reviews were all of medium–high quality based on the AMSTAR tool, the quality of the primary studies needs further improvement in the following areas: publication bias, fewer longitudinal studies and more cross-sectional studies, different measures of predictor(s) and outcome(s), and sample size. These features may have led to low methodological quality, which in turn may increase confusion regarding the certainty of the estimates of the prevalence of perinatal depression and its risk factors.

Despite the aforementioned limitations, to the best of our knowledge, this is the first systematic review of systematic reviews summarising the global prevalence of perinatal depression and its associated risk factors. No publication date or language restrictions were applied and this helped to include and review all systematic reviews focusing on the prevalence and risk factors of perinatal depression. The main strength of this systematic review of systematic reviews is that it brings together reviews of differing quality, in different languages, summarises them and discusses them critically. The results of this study offer a concise and comprehensive review that covers both the estimation of perinatal depression and its common correlates.

Healthcare professionals, including nurses, midwives, and physicians, should be aware of the prevalence and risk factors of perinatal depression globally. Perinatal depression can lead to negative consequences [1, 3, 7–10], therefore the identification of effective and culturally applicable interventions to prevent and treat perinatal depression is crucial. The use of antidepressants and telemedicine was noted to be the most effective therapeutic interventions for treating perinatal depression based on recent research [69]. The early detection of perinatal depression is known to be important regarding the healthcare outcomes of the mothers and their babies. Screening programmes, carried out by well-trained healthcare professionals, are believed to lead to the most effective outcomes [13]. Special attention is needed in assessing and screening for depressive symptoms during both the antenatal and postnatal periods. As the perinatal period is the most common time in which women have contact with their healthcare providers, this is when at-risk women should be identified and supported. It is essential that all healthcare and allied professionals ensure that pregnant patients are appropriately screened for antenatal depression, in order to identify any symptoms of mental illness, such as depression, early and allow for appropriate support during pregnancy, preventing the risk of developing postnatal depression. The development of policies and the integration of mental health services into the primacy care system must be a high priority. To support this, research focusing on understanding the experiences of healthcare professionals, as well as perinatal depression in the primary care system, needs to be expanded upon, to clarify a clear pathway for policies addressing mental health needs in primary healthcare systems.

## Conclusion

This systematic review of systematic reviews found that perinatal depression is highly prevalent and strongly associated with risk factors for depression in the general population, rather than factors associated with pregnancy. Improvements with regards to the quality of the primary research are needed and should aim to be more consistent, namely in: the design of longitudinal studies, representative study samples, standard measurements or scales with standard cut-off points, standard definitions of depression and confounding variables controlled. To align with these recommendations, more consideration is also needed with regards to different cultural expressions of distress and some individuals lack of access to mental health care in some countries.

### Supplementary Information

Below is the link to the electronic supplementary material.

Supplementary file1 (DOCX 15 KB)

Supplementary file2 (DOCX 47 KB)

Supplementary file3 (DOCX 57 KB)

Supplementary file4 (DOCX 29 KB)

Supplementary file5 (DOCX 19 KB)

## Data Availability

This article's data, including tables, is available electronically.

## References

[1] Fisher J, Mello MCD, Patel V, Rahman A, Tran T, Holton S, Holmes W (2012). Prevalence and determinants of common perinatal mental disorders in women in low-and lower-middle-income countries: a systematic review. Bull World Health Organ.

[2] Gavin NI, Gaynes BN, Lohr KN, Meltzer-Brody S, Gartlehner G, Swinson T (2005). Perinatal depression: a systematic review of prevalence and incidence. Obstet Gynecol.

[3] Gelaye B, Rondon MB, Araya R, Williams MA (2016). Epidemiology of maternal depression, risk factors, and child outcomes in low-income and middle-income countries. Lancet Psychiatry.

[4] Gunduz-Bruce H, Takahashi K, Huang MY (2022). Development of neuroactive steroids for the treatment of postpartum depression. J Neuroendocrinol.

[5] Worthen RJ, Beurel E (2022). Inflammatory and neurodegenerative pathophysiology implicated in postpartum depression. Neurobiol Dis.

[6] Beck CT (2001). Predictors of postpartum depression: an update. Nurs Res.

[7] Bonari L, Pinto N, Ahn E, Einarson A, Steiner M, Koren G (2004). Perinatal risks of untreated depression during pregnancy. Can J Psychiatry.

[8] Dagher RK, Bruckheim HE, Colpe LJ, Edwards E, White DB (2021). Perinatal depression: challenges and opportunities. J Womens Health.

[9] Rahman A, Iqbal Z, Bunn J, Lovel H, Harrington R (2004). Impact of maternal depression on infant nutritional status and illness: a cohort study. Arch Gen Psychiatry.

[10] Paulson JF, Dauber S, Leiferman JA (2006). Individual and combined effects of postpartum depression in mothers and fathers on parenting behavior. Pediatrics.

[11] Beck CT (1996). A meta-analysis of predictors of postpartum depression. Nurs Res.

[12] O’hara MW, Swain AM (1996). Rates and risk of postpartum depression—a meta-analysis. Int Rev Psychiatry.

[13] Woody CA, Ferrari AJ, Siskind DJ, Whiteford HA, Harris MG (2017). A systematic review and meta-regression of the prevalence and incidence of perinatal depression. J Affect Disord.

[14] French DP, Cameron E, Benton JS, Deaton C, Harvie M (2017). Can communicating personalised disease risk promote healthy behaviour change? A systematic review of systematic reviews. Ann Behav Med.

[15] Keyworth C, Epton T, Goldthorpe J, Calam R, Armitage CJ (2020). Delivering opportunistic behavior change interventions: a systematic review of systematic reviews. Prev Sci.

[16] Moher D, Liberati A, Tetzlaff J, Altman DG, PRISMA Group* (2009). Preferred reporting items for systematic reviews and meta-analyses: the PRISMA statement. Ann Internal Med.

[17] Shea BJ, Grimshaw JM, Wells GA, Boers M, Andersson N, Hamel C, Bouter LM (2007). Development of AMSTAR: a measurement tool to assess the methodological quality of systematic reviews. BMC Med Res Methodol.

[18] Smith V, Devane D, Begley CM, Clarke M (2011). Methodology in conducting a systematic review of systematic reviews of healthcare interventions. BMC Med Res Methodol.

[19] Sharif MO, Janjua-Sharif FN, Ali H, Ahmed F (2013). Systematic reviews explained: AMSTAR-how to tell the good from the bad and the ugly. Oral Health Dent Manag.

[20] Cohen J (1988) Statistical power analysis Jbr the behavioral. Sciences. Hillsdale (NJ): Lawrence Erlbaum Associates*,* 18–74

[21] Alhasanat D, Fry-McComish J (2015). Postpartum depression among immigrant and Arabic women: literature review. J Immigr Minor Health.

[22] Anderson FM, Hatch SL, Comacchio C, Howard LM (2017). Prevalence and risk of mental disorders in the perinatal period among migrant women: a systematic review and meta-analysis. Arch Womens Ment Health.

[23] Falah-Hassani K, Shiri R, Vigod S, Dennis CL (2015). Prevalence of postpartum depression among immigrant women: a systematic review and meta-analysis. J Psychiatr Res.

[24] Fellmeth G, Fazel M, Plugge E (2017). Migration and perinatal mental health in women from low-and middle-income countries: a systematic review and meta-analysis. BJOG Int J Obstet Gynaecol.

[25] Nilaweera I, Doran F, Fisher J (2014). Prevalence, nature and determinants of postpartum mental health problems among women who have migrated from South Asian to high-income countries: a systematic review of the evidence. J Affect Disord.

[26] Sowa NA, Cholera R, Pence BW, Gaynes BN (2015). Perinatal depression in HIV-infected African women: a systematic review. J Clin Psychiatry.

[27] Vigod SN, Villegas L, Dennis CL, Ross LE (2010). Prevalence and risk factors for postpartum depression among women with preterm and low-birth-weight infants: a systematic review. BJOG Int J Obstet Gynaecol.

[28] Chapman SLC, Wu LT (2013). Postpartum substance use and depressive symptoms: a review. Women Health.

[29] Klaman SL, Turner K (2016). Prevalence of perinatal depression in the military: a systematic review of the literature. Matern Child Health J.

[30] Khatri GK, Tran TD, Fisher J (2019). Prevalence and determinants of symptoms of antenatal common mental disorders among women who had recently experienced an earthquake: a systematic review. BMC Psychiatry.

[31] Ren JH, Chiang CLV, Jiang XL, Luo BR, Liu XH, Pang MC (2014). Mental disorders of pregnant and postpartum women after earthquakes: a systematic review. Disaster Med Public Health Prep.

[32] Mukherjee S, Pierre-Victor D, Bahelah R, Madhivanan P (2014). Mental health issues among pregnant women in correctional facilities: a systematic review. Women Health.

[33] Chen Q, Li W, Xiong J, Zheng X., J. I. j. o. e. r., & health, p (2022) Prevalence and risk factors associated with postpartum depression during the COVID-19 pandemic: a literature review and meta-analysis 19(4):2219 10.3390/ijerph19042219 PMC8872263 35206407

[34] Safi‐Keykaleh M, Aliakbari F, Safarpour H, Safari M, Tahernejad A, Sheikhbardsiri H, Obstetrics (2022) Prevalence of postpartum depression in women amid the COVID‐19 pandemic: a systematic review and meta‐analysis 157(2):240–247 10.1002/ijgo.14129 PMC9087783 35122433

[35] Shorey SY, Ng ED, Chee CY, J. S. J. o. P. H (2021) Anxiety and depressive symptoms of women in the perinatal period during the COVID-19 pandemic: a systematic review and meta-analysis 49(7):730–740 10.1177/14034948211011793 33966511

[36] Desta M, Memiah P, Kassie B, Ketema DB, Amha H, Getaneh T, Sintayehu M (2021). Postpartum depression and its association with intimate partner violence and inadequate social support in Ethiopia: a systematic review and meta-analysis. J Affect Disord.

[37] Yang K, Wu J, Chen X (2022). Risk factors of perinatal depression in women: a systematic review and meta-analysis. BMC Psychiatry.

[38] Alvarez-Segura M, Garcia-Esteve L, Torres A, Plaza A, Imaz ML, Hermida-Barros L, Burtchen N (2014). Are women with a history of abuse more vulnerable to perinatal depressive symptoms? A systematic review. Arch Womens Ment Health.

[39] Beydoun HA, Beydoun MA, Kaufman JS, Lo B, Zonderman AB (2012). Intimate partner violence against adult women and its association with major depressive disorder, depressive symptoms and postpartum depression: a systematic review and meta-analysis. Soc Sci Med.

[40] Choi KW, Sikkema KJ (2016). Childhood maltreatment and perinatal mood and anxiety disorders: a systematic review. Trauma Violence Abuse.

[41] Halim N, Beard J, Mesic A, Patel A, Henderson D, Hibberd P (2018). Intimate partner violence during pregnancy and perinatal mental disorders in low and lower middle income countries: a systematic review of literature, 1990–2017. Clin Psychol Rev.

[42] Han A, Stewart DE (2014). Maternal and fetal outcomes of intimate partner violence associated with pregnancy in the Latin American and Caribbean region. Int J Gynecol Obstet.

[43] Howard LM, Oram S, Galley H, Trevillion K, Feder G (2013). Domestic violence and perinatal mental disorders: a systematic review and meta-analysis. PLoS Med.

[44] Hutchens BF, Kearney J, Kennedy HP (2017). Survivors of child maltreatment and postpartum depression: an integrative review. J Midwifery Womens Health.

[45] Paulson JL (2020) Intimate partner violence and perinatal post-traumatic stress and depression symptoms: a systematic review of findings in longitudinal studies. Trauma Violence Abuse 1524838020976098. 10.1177/1524838020976098 33252020

[46] Shamblaw AL, Cardy RE, Prost E, Harkness KL (2019). Abuse as a risk factor for prenatal depressive symptoms: a meta-analysis. Arch Womens Ment Health.

[47] Wosu AC, Gelaye B, Williams MA (2015). History of childhood sexual abuse and risk of prenatal and postpartum depression or depressive symptoms: an epidemiologic review. Arch Womens Ment Health.

[48] Wu Q, Chen HL, Xu XJ (2012). Violence as a risk factor for postpartum depression in mothers: a meta-analysis. Arch Womens Ment Health.

[49] Zhang S, Wang L, Yang T, Chen L, Qiu X, Wang T, Qin J (2019). Maternal violence experiences and risk of postpartum depression: a meta-analysis of cohort studies. Eur Psychiatry.

[50] Arafa A, Dong JY (2019). Gestational diabetes and risk of postpartum depressive symptoms: a meta-analysis of cohort studies. J Affect Disord.

[51] Azami M, Badfar G, Soleymani A, Rahmati S (2019). The association between gestational diabetes and postpartum depression: a systematic review and meta-analysis. Diabetes Res Clin Pract.

[52] Barakat S, Martinez D, Thomas M, Handley M (2014). What do we know about gestational diabetes mellitus and risk for postpartum depression among ethnically diverse low-income women in the USA?. Arch Womens Ment Health.

[53] Brown HK, Qazilbash A, Rahim N, Dennis CL, Vigod SN (2018). Chronic medical conditions and peripartum mental illness: a systematic review and meta-analysis. Am J Epidemiol.

[54] Delahaije DH, Dirksen CD, Peeters LL, Smits LJ (2013). Anxiety and depression following preeclampsia or hemolysis, elevated liver enzymes, and low platelets syndrome. A systematic review. Acta Obstet Gynecol Scand.

[55] Suzuki D, Wariki WM, Suto M, Yamaji N, Takemoto Y, Rahman MM, Ota E (2019). Association of secondhand smoke and depressive symptoms in nonsmoking pregnant Women: a systematic review and meta-analysis. J Affect Disord.

[56] Bhati S, Richards K (2015). A systematic review of the relationship between postpartum sleep disturbance and postpartum depression. J Obstet Gynecol Neonatal Nurs.

[57] Bowen A, Muhajarine N (2006). Antenatal depression. Can Nurse.

[58] Ayano G, Tesfaw G, Shumet S (2019). Prevalence and determinants of antenatal depression in Ethiopia: a systematic review and meta-analysis. PLoS ONE.

[59] Getinet W, Amare T, Boru B, Shumet S, Worku W, Azale T (2018) Prevalence and risk factors for antenatal depression in Ethiopia: systematic review. Depress Res Treat 10.1155/2018/3649269 PMC6077581 30112199

[60] Sawyer A, Ayers S, Smith H (2010). Pre-and postnatal psychological wellbeing in Africa: a systematic review. J Affect Disord.

[61] Liu X, Wang S, Wang G (2021) Prevalence and risk factors of postpartum depression in women: a systematic review and meta‐analysis. J Clin Nurs 10.1111/jocn.16121 34750904

[62] Xiao RS, Kroll-Desrosiers AR, Goldberg RJ, Pagoto SL, Person SD, Waring ME (2014). The impact of sleep, stress, and depression on postpartum weight retention: a systematic review. J Psychosom Res.

[63] Jha S, Salve HR, Goswami K, Sagar R, Kant S (2018). Burden of common mental disorders among pregnant women: a systematic review. Asian J Psychiatr.

[64] Gaynes BN, Gavin N, Meltzer-Brody S, Lohr KN, Swinson T, Gartlehner G, Miller WC (2005) Perinatal depression: prevalence, screening accuracy, and screening outcomes: summary. AHRQ evidence report summaries 10.1037/e439372005-001 PMC4780910 15760246

[65] Carter FA, Frampton CM, Mulder RT (2006). Cesarean section and postpartum depression: a review of the evidence examining the link. Psychosom Med.

[66] Moameri H, Ostadghaderi M, Khatooni E, Doosti-Irani A (2019). Association of postpartum depression and cesarean section: a systematic review and meta-analysis. Clin Epidemiol Global Health.

[67] Olieman RM, Siemonsma F, Bartens MA, Garthus-Niegel S, Scheele F, Honig A (2017). The effect of an elective cesarean section on maternal request on peripartum anxiety and depression in women with childbirth fear: a systematic review. BMC Pregnancy Childbirth.

[68] Xu H, Ding Y, Ma Y, Xin X, Zhang D (2017). Cesarean section and risk of postpartum depression: a meta-analysis. J Psychosom Res.

[69] Chow R, Huang E, Li A, Li S, Fu SY, Son JS, Foster WG (2021). Appraisal of systematic reviews on interventions for postpartum depression: systematic review. BMC Pregnancy Childbirth.

