# Correction to: Prevalence and correlates of perinatal depression

**DOI:** 10.1007/s00127-023-02468-2

**Published:** 2023-04-20

**Authors:** Khalood Al-abri, Dawn Edge, Christopher J. Armitage

**Affiliations:** 1 Division of Psychology and Mental Health, University of Manchester, G35 Coupland 1 Building, Manchester, UK; 2 Department of Community and Mental Health, College of Nursing, Sultan Qaboos University, Muscat, Oman; 3 Equality, Diversity and Inclusion Research Unit, Greater Manchester Mental Health NHS Trust, Manchester, UK; 4 Manchester University NHS Foundation Trust, Manchester Academic Health Science Centre, Manchester, UK; 5 NIHR Greater Manchester Patient Safety Translational Research Centre, University of Manchester, Manchester, UK

**Correction to: Social Psychiatry and Psychiatric Epidemiology** 10.1007/s00127-022-02386-9

In this article supplementary table incorrectly processed as the text table. It has been corrected in original article.

The original article has been corrected.

