# Supplementary material for: Prevalence and correlates of perinatal depression
Source: Soc Psychiatry Psychiatr Epidemiol. 2023 Jan 16;58(11):1581–90. doi: 10.1007/s00127-022-02386-9 (PMC9842219; doi:10.1007/s00127-022-02386-9)
Supplement: Supplementary file 1 — Supplementary file1 (DOCX 15 KB) [file 127_2022_2386_MOESM1_ESM.docx]

- **Full search terms for one database (CHINAL 1/04/2022)**

| Search | Add to builder | Query | Items found |
| --- | --- | --- | --- |
| #5 | [Add](https://www.ncbi.nlm.nih.gov/pubmed/advanced) | ( depress* OR depression OR depressive disorder OR depressive symptoms OR major depressive disorder OR “ major depression” OR “ major depressive episode” ) AND ( risk factors OR contributing factors OR predisposing factors ) AND ( systematic review* OR “meta-analysis* ) AND ( “ pregan*” OR “ pregnancy” , OR “ gestation” OR “ prena*” OR “ prenatal” OR “ puerperium” OR “ antepartum” , OR “ ante-partum” OR “ antenatal” OR “ ante-natal” OR “ perinatal” , OR “ postnatal” , OR “ post-natal” OR” Postpartum” OR “ post-partum” ) | 137 |
| [#4](https://www.ncbi.nlm.nih.gov/pubmed/advanced) | [Add](https://www.ncbi.nlm.nih.gov/pubmed/advanced) | depress* OR depression OR depressive disorder OR depressive symptoms OR major depressive disorder OR “ major depression” OR “ major depressive episode” ) AND ( prevalence OR epidemiology OR proportion OR frequency OR occurrence ) AND ( risk factors OR contributing factors OR predisposing factors ) AND ( systematic review* OR “meta-analysis* ) AND ( “ pregan*” OR “ pregnancy” , OR “ gestation” OR “ prena*” OR “ prenatal” OR “ puerperium” OR “ antepartum” , OR “ ante-partum” OR “ antenatal” OR “ ante-natal” OR “ perinatal” , OR “ postnatal” , OR “ post-natal” OR” Postpartum” OR “ post-partum” ) | 52 |
| [#3](https://www.ncbi.nlm.nih.gov/pubmed/advanced) | [Add](https://www.ncbi.nlm.nih.gov/pubmed/advanced) | ( antenatal depression and postpartum depression and postnatal depression ) AND ( prevalence OR epidemiology OR proportion OR frequency OR occurrence ) OR ( risk factors OR contributing factors OR predisposing factors ) AND ( systematic review* OR “meta-analysis* ) | (15,410) |
| [#2](https://www.ncbi.nlm.nih.gov/pubmed/advanced) | [Add](https://www.ncbi.nlm.nih.gov/pubmed/advanced) | ( “ perinatal depression” (MeSH) OR ‘’antenatal depression’’ (MeSH) OR ‘’postpartum depression’’ (MeSH) ) AND ( prevalence OR epidemiology OR proportion OR frequency OR occurrence ) OR ( risk factors OR contributing factors OR predisposing factors ) AND ( systematic review* OR “meta-analysis* ) | (15,350) |
| [#1](https://www.ncbi.nlm.nih.gov/pubmed/advanced) | [Add](https://www.ncbi.nlm.nih.gov/pubmed/advanced) | ( depress* OR depression OR depressive disorder OR depressive symptoms OR major depressive disorder OR “ major depression” (MeSH), “ major depressive episode” (MeSH) ) AND ( prevalence OR epidemiology OR proportion OR frequency OR occurrence ) OR ( risk factors OR contributing factors OR predisposing factors ) AND ( systematic review* OR “meta-analysis* ) AND ( motherhood OR pregan* OR pregnancy OR gestation OR prena* OR prenatal OR puerperium OR antepartum OR ante-partum OR antenatal OR ante-natal OR perinatal OR postnatal OR post-natal OR Postpartum OR post-partum ) | 39,114 |
