# Supplementary material for: Prevalence and correlates of perinatal depression
Source: Soc Psychiatry Psychiatr Epidemiol. 2023 Jan 16;58(11):1581–90. doi: 10.1007/s00127-022-02386-9 (PMC9842219; doi:10.1007/s00127-022-02386-9)
Supplement: Supplementary file 2 — Supplementary file2 (DOCX 47 KB) [file 127_2022_2386_MOESM2_ESM.docx]

Table 1: Characteristics of the included systematic reviews related to the prevalence of perinatal depression.

| **No.** | **First author** | **Publication year** | **Title of Review** | **Review Objective or Aims** | **Number of Included Studies** | **Type of Studies** | **Sample Size** | **Characteristics of Population** | **Countries** | **Time of Measurements/ Time points** | **Type of Depression Measurements** | **Key Findings** |
| --- | --- | --- | --- | --- | --- | --- | --- | --- | --- | --- | --- | --- |
| 1 | Alhasanat | 2015 | Postpartum Depression Among Immigrant and Arabic Women: Literature Review. | To identify the prevalence and risk factors of postnatal depression among immigrant women of Arabic descent in selected industrialized countries and to compare it with the prevalence and risk factors for postnatal depression among Arabic women who are living in their native countries. | 26 | Immigrant women = 14 studies (8 cross-sectional, 3 longitudinal, 1 secondary analysis of data from a cross-sectional study, 2 analyses of national survey data). Native women = 12 studies (5 longitudinal, 6 cross-sectional, 1 comparative descriptive). | Immigrant women: ranged from 60 to 2,137. Native women: 95 to 2,326. | Adult Arabic women aged 18 years plus, either immigrants or native, recruited from urban hospitals or community health centres. | Immigrants to: Canada, Taiwan, Australia, USA. Native countries: United Arab Emirates (UAE), Morocco, Jordan, Lebanon, Bahrain, Egypt, Qatar, Israel | Measurements of depression ranged from 1st trimester of their pregnancy to 12 months postnatal. | Immigrant women: 10 studies used the EPDS, 1 self-report of depressive feelings and SF36 with EPDS, 2 CES-D, 2 PDSS-S. Native women = 12 studies used the EPDS, 3 EPDS and MINI, 1 DSM IV-TR. | Prevalence of postnatal depression ranged from: 11.2-60% in Immigrant women in industrialized countries. Native women: 10–37%. Risk factors and postnatal depression among all: lack of social support, lack of emotional support from spouse or partner, stressful life events, low income & intimate partner violence. For immigrant women, immigration stress, lack of access to health care services, lack of social support was found to be more predominant. |
| 2 | Alhasanat | 2017 | Acculturation and postpartum depressive symptoms among Hispanic women in the United States: Systematic review. | To examine the relationship between acculturation and postnatal depression among immigrant or refugee women in the United States. | 7 | 3 longitudinal studies, 4 cross-sectional studies. | Sample size ranged from 66 to 3,952 women. | Hispanic, young (mean age 23–28 years). 1 study ranged from 21 to 40 years old. All studies had primiparous or multiparous participants. Most studies had participants of low socioeconomic status (annual family income of ≤$20,000). | USA | Depression measured during pregnancy or immediately postnatal and up to 13 months postnatal. | 4 CES-D, 2 BDI-FS, 1 PDSS-S. Acculturation measures: Country of birth, Country of residence, Language preferences used by most studies to measure acculturation. | Prevalence of postnatal depressive symptoms of Hispanic women in the USA ranged from 18-59%. Higher risk of postnatal depressive symptoms was associated with higher levels of acculturation. |
| 3 | Anderson | 2017 | Prevalence and risk of mental disorders in the perinatal period among migrant women: a systematic review and meta-analysis. | To examine the prevalence and risk of mental health disorders among migrant women during pregnancy and the postnatal period. | 53 | 31 cross-sectional surveys, 20 prospective cohorts, 2 randomized controlled trials. | Prevalence of antenatal depression was 5110 migrant women across 16 studies, postnatal depression was 15,153 migrant women across 33 studies. | Migrant women were included only for prevalence estimates, not for the estimates of risk as this required non-migrant participants. | USA, Canada, Australia, Taiwan, China | 6 studies measured symptoms of mental health disorders in both antenatal and postnatal women, presenting data separately, 3 studies measured symptoms in both periods but combined the data, 33 measured symptoms in postnatal women only, 11 only in the antenatal period. Time points for postnatal measurements ranged from 1 week to 12 months. | EPDS, CES-D, PHQ-9, diagnostic psychiatric interview, BDI-FS, BDI-II DASS MMIS PDSS. | Migrant women: median prevalence of antenatal depression was 28%, estimates ranging from 12-45%. Median prevalence of postnatal depression was 19%, with estimates ranging from <1-59%. Migrants to Canada had an association with increased risk for antenatal (OR = 1.86, 95% CIs 1.32–2.62) and postnatal depression (OR = 1.98, 95% CIs 1.57–2.49). Migrants to the USA showed a decreased risk of antenatal depression (OR = 0.71, 95% CIs 0.51–0.99). Studies from the USA and Australia found no association between migrant status and increased risk of postnatal depression. Risk factors of increased perinatal mental health disorders (e.g. depression) were: low social support, minority ethnicity, and low socioeconomic status, lack of language proficiency and refugee or asylum-seeking status. |
| 4 | Ayano | 2019 | Prevalence and determinants of antenatal depression in Ethiopia: A systematic review and meta-analysis. | To systematically examine the prevalence and risk factors of antenatal depression in Ethiopia. | 5 | 5 cross-sectional studies. | Total sample size: 2126. | Pregnant women from community and institutions. | Ethiopia | 2 studies measured depression in the 1st trimester of pregnancy, 4 measured depression in the 2nd and 3rd trimesters of pregnancy. | EPDS BDI | Pooled prevalence of antenatal depression in Ethiopia was 21.28% (95% CI; 15.96–27.78). Highest prevalence in the 3rd trimester was 32.10%; 2nd trimester was 18.86%; 1st trimester was 19.13%. Prevalence was measured at 26.48% (BDI) and 18.28% (EPDS). Community setting prevalence was 15.50% c.f. 25.77% in the institution settings. Risk factors of antenatal depression were: a history of stillbirth, complications during pregnancy, past history of depression, no or irregular follow-ups or not satisfied by ANC, low income, partner violence, food insecurity, low or median social support, unmarried, younger age (20-29 years), housewives and farmers. |
| 5 | Azami | 2018 | The prevalence of depression in pregnant Iranian women: A systematic review and meta-analysis. | To estimate the prevalence of depression in pregnant Iranian woman. | 40 | Not reported. | Total sample size was 15,870 pregnant Iranian women. | Mean age across 13 studies was 25.90 ± 0.36 years. | Iran | Depression was measured in the 1st, 2nd and 3rd trimesters of pregnancy. | Beck’s questionnaire, GHQ, PHQ | Very high heterogeneity (I2 = 99.70%, P < 0.001). Estimated prevalence of antenatal depression was 41.22% (95% CI: 33.88 48.96). Prevalence estimates: mild depression was 23.71% (95% CI: 16.39 - 33.01); moderate depression was 10.67% (95% CI: 7.02-15.98); severe depression was 4.30% (95% CI: 2.33-7.80).West Iran: lowest estimated prevalence was 34.41% (95% CI: 17.38-56.67); South Iran: highest estimated prevalence was 51.69% (95% CI: 37.32-65.78). Trimesters and prevalence of depression: 1st trimester was 50.52% (95% CI: 39.91-61.08), 2nd trimester was 44.96% (95% CI: 26.92-64.42), 3rd trimester was 48.33% (95% CI: 36.6460.20). |
| 6 | Bennett | 2004 | Prevalence of depression during pregnancy: systematic review. | To systematically identify the prevalence of depression during pregnancy by various trimesters, as identified by validated screening instruments (e.g. EPDS and BDI) and structured interviews, to compare the prevalence rates among those instruments. | 21 | Cross-sectional longitudinal studies. | Sample sizes ranged from 29 to 14890 (mean 409). | Generally recruited from urban populations (n = 18); 15 had participants with diverse socioeconomic status, 3 recruited participants from obstetric and prenatal units. Mean age of participants was 27.7 years. | Majority was from USA, also UK, Canada, Sweden, Finland, Spain, Australia, France, Hong Kong, Brazil, Portugal, and Japan. | Within 1st, 2nd and 3rd trimester of pregnancy. | 8 used BDI and EPDS, 7 structured interviews, 2 BDI and structured Interviews. | Prevalence of depression in 1st trimester was 7.4% (95% CI = 2.2, 12.6), 2nd trimester it increased to 12.8% (95% CI = 10.7, 14.8), later pregnancy was 12.0% (95% CI = 7.4, 16.7). Self-reported 2nd trimester rates was 47% for pregnant women of low socioeconomic status, 39% for the 3rd trimester. Structured interviews: the rates were lower at 28% in the 2nd trimester, 25% in the 3rd trimester. These rates were higher in these groups than in the general population. |
| 7 | Chapman | 2013 | Postpartum substance use and depressive symptoms: a review. | To examine the association between postpartum substance use and postnatal depression. | 6 | 6 examined the association between postpartum substance use and postnatal depression, longitudinal studies. | Sample sizes of ≤595. | Participants were recruited from teaching hospitals or prenatal units. | White, Native American, Black and Hispanic women. | Pregnancy to 6 months postnatal. | Not reported. | Only 6 studies examined the prevalence of postnatal depression among substance-using mothers. Findings: postnatal substance use and mothers with a history of substance use had higher prevalence rates of postnatal depression estimated at 19.7–46% c.f. women who were not substance users or had a history of it. |
| 8 | Falah-Hassani | 2017 | The prevalence of antenatal and postnatal co-morbid anxiety and depression: a meta-analysis. | To examine the prevalence of co-morbid anxiety and depression in the perinatal period. | 66 | Not reported. | Total: 162120 women. | Not reported. | USA, Australia, Brazil, Canada, the Netherlands, France, Germany, Greece, Italy, New Zealand, Norway, Portugal, Singapore, Tanzania, UK, Vietnam, Bangladesh, Croatia, Ghana, Hong Kong, Hungary, Ireland, Israel, Japan, Nigeria, Poland, Romania, Saudi Arabia, Switzerland, Turkey. | Within pregnancy. | EPDS, BDI, HADS, DASS, HSCL-25, SRDS, clinical diagnosis. | Prevalence rates of antenatal anxiety and depression (mild to severe) identified by self-report was 9.5% (95% CI: 7.8–11.2), co-morbid anxiety symptoms and moderate/severe depressive symptoms was 6.3% (95% CI: 4.8–7.7). Prevalence of antenatal anxiety disorder and depression identified by clinical diagnosis was 9.3% (95% CI: 4.0–14.7), co-morbid generalized anxiety disorder and depression was 1.7% (95% CI: 0.2–3.1). The prevalence of co-morbid anxiety and mild/severe depression was 8.2% (95%: CI 6.5–9.9) between 1- 24 weeks postnatally, while co-morbid anxiety and moderate/severe depressive symptoms was 5.7% (95% CI: 4.3-7.1, 13 studies, n = 20849). The prevalence of co-morbid anxiety and depression identified by clinical diagnosis was 4.2% (95% CI: 1.9–6.6). |
| 9 | Falah-Hassani, | 2015 | Prevalence of postpartum depression among immigrant women: a systematic review and meta-analysis. | To examine the prevalence of postnatal depression in immigrant women, compared to non-immigrant women, and identify factors of postnatal depression among immigrant women. | 22 | 12 cross-sectional studies, 10 prospective cohort | Sample size ranged from 61 to 24,455. | Recruited from general population, hospitals/health centres. | Canada, Taiwan, Australia, USA , Israel, Sweden, Norway, Switzerland, UK. | Ranged from 1 week to 1 year postpartum. | EPDS, CES-D | Prevalence of postnatal depression among 13,749 immigrant women was estimated at 20% (95% CI: 17–23%, N = 18). It was found that 50,519 immigrant women were at a 1.5-2 fold higher risk of experiencing postnatal depression than non-immigrant women (pooled unadjusted odds ratio [OR] = 2.10; 95% CI: 1.62–2.73, 15 studies; adjusted OR = 2.18, 95% CI 1.60–2.96, 7 studies) in meta-analysis consisting of 35,557 women. Risk factors associated with postnatal depression in immigrant women were: a decreased length of residence in the destination country, lack or low level of social support, difficulties with marital adjustment, inadequate family income. |
| 10 | Fellmeth | 2017 | Migration and perinatal mental health in women from Low and Middle‐Income Countries: a systematic review and meta‐analysis. | To examine the prevalence, risk factors and interventions for perinatal mental disorders among migrant women originating from Low and Middle-Income Countries (LMIC) who have resettled in another country. | 40 | case‐control, cross‐sectional, cohort or intervention study design. | 19,349 women, sample sizes ranged from 31 to 909 migrant women. | Mean age range: 24-33 years. 33 studies included postpartum women. 2 included refugees or asylum seekers, 14 included a non‐migrant comparison group of which 9 reported higher rates of mental illness among migrant groups. | 22 from North America, 6 Europe, 6 Australasia. | Not reported. | EPDS (23 studies), CES-D (9 studies), BDI-II PDSS | The pooled prevalence of any depressive disorder and major depressive disorder were 31.4% (95% CI 23.2–40.2%, P = 0.00, I2 96.4%) and 17.3% (95% CI 12.4–22.8%, P = 0.00, I2 92.5%) respectively. Perinatal depression appears to be a major mental health issue in migrant women, affecting possibly 1 in 3 women. More than 1 in 5 women may be affected by a major depressive disorder. Risk factors for perinatal depression: history of previous depression, lack of social support for migrant women. Social isolation may be intensified by language and culture obstacles leading to significant suffering for new mothers lacking support from their resettlement country. |
| 11 | Fisher | 2012 | Prevalence and determinants of common perinatal mental disorders in women in Low and Lower-Middle-Income Countries: a systematic review. | To examine the prevalence and risk factors of of non-psychotic common perinatal mental disorders (CPMDs) in Low and Lower-Middle-Income Countries (LMIC). | 47 | Prospective studies, cohort studies, cross-sectional studies, 3 reported prevalence data about CPMDs in pregnant women, 34 reported prevalence data during 1st year postnatal. | Ranged from 65 to 1065. | Participants mainly from health facilities/tertiary teaching hospitals. Some women from community-based health services & via household visits. | Low and Lower-Middle-Income Countries. | Pregnancy (trimesters 1, 2 and 3) and up to 1 year postnatally. | Self-reported, EPDS, GHQ, MINI, diagnostic assessment. | Antenatal data on CPMDs including depression was available for 9 countries, showing a prevalence of 15.6% (95% confidence interval, CI: 15.4-15.9). Postnatal data on CPMDs was available for 17 countries, showing a prevalence of 19.8% (19.5-20.0). Meta-analysis showed significant differences between: antenatal prevalence identified by self-report symptom measures (13.43%; 95% CI: 12.4-14.5), and that was identified by diagnostic assessment (21.75 %; 95% CI: 19.8-23.7). The mean prevalence identified by each measure was also significant: self-report (20.80%; 95% CI: 20.0-21.6) compared to diagnostic assessments (16.09%; 95% CI: 14.6-17.6). CPMD risk factors involved: socioeconomic disadvantage (OR range: 2.1-13.2); unintended/unwanted pregnancy (1.6-8.8); younger women (2.1-5.4); being unmarried (3.4-5.8); lacking partner support (2.0-9.4); conflict with in-laws (2.1-4.4); experiencing intimate partner violence (2.11-6.75); lack of emotional and practical support (2.8-6.1); having a daughter not a son (1.8-2.6); having a history of mental illness (5.1-5.6). Protective factors: being more educated (relative risk: 0.5; P = 0.03); having a secure job (OR: 0.64; 95% CI: 0.4-1.0); being or belonging to an ethnic majority (OR: 0.2; 95% CI: 0.1-0.8) having kindness and support from intimate partner (OR: 0.52; 95% CI: 0.3-0.9). |
| 12 | Gavin, N. I., | 2005 | Perinatal depression: a systematic review of prevalence and incidence. | To identify the prevalence and incidence of perinatal depression and compare these with prevalence of depression in women at non-childbearing times. | 28 | Cross-sectional studies, cohort studies, case-control studies. | Sample sizes ranged from 54 to 4,964 women. | Women had a limited racial/ethnic mix. | USA, UK, Japan, Canada, Hong Kong, Spain, Australia, Norway, Scotland, Portugal. | Pregnancy and 1st year postnatal. | Structured clinical interview, some added EPDS and GHQ | 1st trimester of pregnancy registered the highest point of prevalence of depression, around 11.0% (95% CI: 7.6-15.8%), 2nd and 3rd trimesters saw a decrease to an average prevalence of 8.5% (95% CI: 6.6–10.9%/6.5–11.0%) within the same group. The highest prevalence of major and minor depression was 12.9% (95% CI: 10.6-15.8%) in the 3rd month postpartum, dropping to 6.5% (95% CI: 4.0–9.9%, 2.7–12.9%) between 8-12 months postpartum. Estimation of the prevalence of major or minor depression was 18.4% (95% CI: 14.3–23.3%) and 19.2% (95% CI: 10.7–31.9%) during pregnancy to 3 months postnatal. The incidence of new major and minor depressive episodes was less at 14.5% during this period. No conclusions were drawn regarding the association between incidence of depression among perinatal women compared to women at non-childbearing times. |
| 13 | Gelaye | 2016 | Epidemiology of maternal depression, risk factors, and child outcomes in Low-Income and Middle-Income Countries. | To systematically identify the prevalence and risk factors of perinatal depression; and the relationship bewteen perinatal depression and infant and childhood outcomes among women who are originally from Low-Income and Middle-Income Countries (LMIC). | 104 | Not reported. | Total: 87046 participants. | Not reported. | LMIC e.g. Brazil, Turkey, South Africa, China, Pakistan, Bangladesh, Ethiopia, Ghana, Jamaica, Jordan, Malawi, Malaysia, Mexico, Nepal, Papua New Guinea, Peru, Tanzania, Thailand, Vietnam. | During pregnancy and 1 year postnatal. | PHQ-9, EPDS, BDI, MINI, SCID, HDRS, AKUADS, CIDI, JHSC, PCEMD, SRDS, SRQ | The prevalence of prenatal depression was 25.8% (95% CI: 22·8–29·0%). The prevalence of postnatal depression was 19.7% (95% CI: 16·9–22·8%). Finding: perinatal depression was significantly associated with: pregnant women maltreated as children (physically, emotionally, sexually abused) or who experienced intimate partner violence in one or all of those areas, had a low education level, low socio-economic status, poor or lacking social support. Perinatal depression is accompanied by negative effects on child development, e.g. in their physical and neurocognitive growth. |
| 14 | Getinet | 2018 | Prevalence and Risk Factors for Antenatal Depression in Ethiopia: Systematic Review. | To examine the prevalence and risk factors of antenatal depression in Ethiopia and to examine the association between unplanned pregnancy and antenatal depression. | 9 | 7 cross-sectional, 2 cohort studies. | Not reported. | Ethiopian women, recruited from attending antenatal care service | Ethiopia | During pregnancy. | EPDS, BDI, PHQ | BDI findings on antenatal depression: 5 studies pooled prevalence based on BDI was 25.33 (20.74, 29.92). EPDS findings on antenatal depression: 3 studies pooled prevalence based on EPDS was 18.73 (11.30, 26.17). Mixed measures: 9 studies pooled prevalence of antenatal depression using BDI (n = 5), EPDS (n = 3), and PHQ (n = 1) was 23.56 (19.04,28.07). Risk factors for antenatal depression were: age (younger age during pregnancy), marital status (being unmarried or single ), low income, occupation, history of previous mental disorder, previous irregular antenatal follow-up, unplanned/unwanted pregnancy, pregnancy related complication, marital conflict, lack of social support. |
| 15 | Hahn-Holbrook | 2018 | Economic and health predictors of national postpartum depression prevalence: a systematic review, meta-analysis, and meta-regression. | To identify the global prevalence of postpartum depression (PPD) and the economic and health predictors of PPD. | 291 | Not reported. | 296, 284 women. | Not reported. | Global countries. | Earlier or later in the postpartum period. | Based on EPDS | Global prevalence of postnatal depression was estimated at about 17.7% (95% CI: 16.6–18.8%). After adjustment of the EPDS cut offs for possible (≥10) and probable depression (≥13), a possible depression prevalence was found to be 21.3%, probable depression was 16.7%. These estimates were found to be significantly higher than the 13% found in studies of High Income Countries, closer to the 19% prevalence of postnatal depression meta-analyses of Low & Middle-Income Countries. The highest rates of postnatal depression were found to be in: Chile (38%, 95% CI: 35–41%), South Africa (37%; 95% CI: 31–42%), Hong Kong (30%, CI: 28–31%), Turkey (28%, CI: 27–29%). The lowest rates of postnatal depression were estimated to be in: Singapore (3%; 95% CI: 2–5%), Nepal (7%; 95% CI: 5–10%), the Netherlands (8%; 95% CI: 7–9%), Switzerland (11%; 95% CI: 7–13%). Predictors of higher rates of postnatal depression accounted for 73% of nations' variation in postnatal depression prevalence: countries with a significantly higher estimation rate of income inequality (R2 = 41%), maternal mortality (R2 = 19%), infant mortality (R2 = 16%), younger mothers working ≥40 hours a week (R2 = 31%) |
| 16 | Halbreich | 2006 | Cross-cultural and social diversity of prevalence of postpartum depression and depressive symptoms. | To estimate the prevalence of postnatal depression among a wide range of countries. | 143 | Not reported. | Not reported. | Not reported. | 40 countries, most were Western economically developed countries. | Within 1 year postpartum. | EPDS, CES-D, BDI, GHQ, clinical interview. | Prevalence of postnatal depression varies among countries, ranging from 0% to almost 60%. In Singapore, Malta, Denmark and Malaysia, for example, there are very few reports of the prevalence of postnatal depression, ranging from 0.5–9% In Guyana, Costa Rica, Italy, South Africa, Korea and Taiwan, for example, the higher prevalence was estimated to range from 34-57%. |
| 17 | Jha | 2018 | Burden of common mental disorders among pregnant women: A systematic review. | To examine the burden of common mental health disorders (CMD) among pregnant women. | 23 | 17 cross-sectional, 6 cohort/longitudinal. | 15,794 pregnant women. | Recruited at household level via health facility/primary care settings, tertiary care/medical college. | Low, Upper Middle & High income countries. | Different trimesters of pregnancy. | EPDS, BDI, PHQ, MINI, STAI, SCAN, SCID, DSM IV, SI for ICD 10. | The prevalence of CMDs among pregnant women ranged from 1–37%. Depression: prevalence ranged from 1–30%. Anxiety: prevalence ranged from 1–26%. Risk factors for CMDs included: lower socioeconomic status (e.g. poverty or food insecurity), intimate partner violence, history of previous of CMDs, history of mental illness among family, single marital status. Effect of CMDs on child health outcome: prematurity and low birth weight. |
| 18 | Jones | 2013 | Social relationships and postpartum depression in South Asia: A systematic review. | To examine the association between social relationships and postnatal depression in South Asia. | 9 | 3 cross-sectional, 5 longitudinal, 1 qualitative study. | Quantitative sample sizes ranged from 137 to 632 women. | Recruited from community-based samples and a hospital-based antenatal clinic. | Nepal, Bangladesh, India, Pakistan. | 2–12 weeks postpartum. | ICD diagnostic criteria, EPDS. | Prevalence of postnatal depression ranged from a very low, 4.9% in Nepal, to 19.8–35.6% in Bangladesh, India and Pakistan. Risk factors: low or lacking social support, poor relationships with husband and parents-in-law (but multivariate analyses showed that relations were weakened). Complex associations of social relationship risk factors that are not yet well understood and diverse aspects of support that have not yet been systematically studied. |
| 19 | Karaçam | 2018 | Status of postpartum depression in Turkey: A meta-analysis. | To identify the prevalence of postnatal depression and risk factors in Turkey. | 47 | 46 cross-sectional, 1 descriptive, 1 case-control observational design. | Sample sizes varied between 49 to 2,514. | Field-data and data obtained in hospital. | Turkey | 0–12 months postnatal. | EPDS, BDI, ZSDS | High risk of postnatal depression: 4,740 of 18,780 women. Prevalence in Turkey was 24% (95% CI: 21–27%), rate varying between 9%–51%. Highest prevalence was found to be 36.4% in the Mediterranean region. Lowest prevalence was 19.5% in the Marmara Region. 54 risk factors of postnatal depression were identified, such as history of mental illness before pregnancy, history of unplanned or unwanted pregnancies, income level or sociodemographic status, marital conflicts feeling unhappy displeasure with spouse or marital life and being a housewife. |
| 20 | Khatri | 2019 | Prevalence and determinants of symptoms of antenatal common mental disorders among women who had recently experienced an earthquake: a systematic review. | To examine the prevalence and risk factors of antenatal common mental disorders (CMDs) among women who had recently experienced an earthquake. | 7 | Cross-sectional surveys. | Sample size ranged from 99 to 670 women who experienced an earthquake. 6803 were used as a comparison control Group. | 6 studies recruited from hospital antenatal clinics 1 from antenatal clinic or local government offices issuing pregnancy journals, Mother-Child Health Handbook. Only 3 had a comparison control group. | Japan, Taiwan, China. | During pregnancy. | EPDS, K6, CHQ-12 | Significant symptoms of antenatal CMD prevalence in post-earthquake survivors ranged from 4.6% (95% CI: 3.2; 6.5) to 40.8% (95% CI: 35.5; 46.4), It was called ‘psychological stress’ in Japan and ‘depression’ in China. 2 of the 4 studies in China reported women who had experienced earthquakes during gestation, prevalence of antenatal depressive symptoms was 7.1% (95% CI: 5.1; 9.5) and 35.2% (95% CI: 26.9; 44.1) using EPDS with same cut-off score ≥14. The other 2 studies found that the prevalence of depressive symptoms among women who had experienced earthquake 18 months was 40.8% (95% CI: 35.5; 46.4) and 4 years 34.5% (95% CI: 28.9; 40.6; based on the EPDS with a cut-off score ≥10) before pregnancy. Risk factors were: marital conflict, poor/lacking social support, multiparty stresses, life events during pregnancy, having a negative coping style. |
| 21 | Klainin | 2009 | Postpartum depression in Asian cultures: a literature review. | To identify risk factors for postnatal depression among women in Asian cultures. | 64 | 61 studies were quantitative, 3 were qualitative. The majority were cross-sectional, remainder were cohort studies. | Sample sizes ranged from 11to 2514. | Recruited from hospitals/medical centres, antenatal clinics and community-based settings. | 17 Asian countries: China, Hong Kong, India, Indonesia, Iran, Israel, Japan, Lebanon, Malaysia, Nepal, Pakistan, Singapore, Taiwan, Thailand, Turkey, United Arab Emirates, Vietnam. | Range: 3 weeks-1 year postpartum. | EPDS, CIS-R, SCAN, SCID-IV, SCI based on DSM-III, CES-D, PDRS, PDS. | Prevalence of postnatal depression among 17 Asian countries ranged from 3.5-63.3%. Highest rate: Malaysia. Lowest rate: Pakistan. Risk factors: biological/physical factors (e.g., history of medical conditions, poor nutritional status e.g. low riboflavin (vitamin B2), low level of docasahexaenoic acid (DHA), psychological (e.g. antenatal depression, history of mental illness, stressful life events), obstetric/paediatric (e.g. unwanted pregnancy, the absence of breastfeeding, infant with medical problems), socio-demographic (e.g. poverty), cultural factors (e.g. preference of infants’ gender and conflicts with mother-in-law), traditional postnatal rituals did not establish psychological benefits for new mothers. |
| 22 | Klaman | 2016 | Prevalence of perinatal depression in the military: A systematic review of the literature. | To examine the prevalence of perinatal depression among military service women and spouses of military servicemen. | 10 | cross-sectional and longitudinal studies | Sample sizes ranged from 82 to 3,956. | Military service women and military spouses. | Not reported. | Pregnancy (all 3 trimesters) and 6 months postnatal. | EPDS, ICD-9 diagnostic codes, PHQ-9, PDSS | Prevalence of perinatal depression in 10 studies ranged from 4.6-50.7%. Military women: prevalence ranged from 11.3-24%. Military spouses: prevalence ranged from 4.6-50.7%. Both military women and spouses: prevalence ranged from 6.1-16.4%. Deployment status during pregnancy and postpartum produces a unique risk factor for perinatal depression among military populations. |
| 23 | Lobato | 2011 | The extent of post-partum depression in Brazil: a systematic review. | To identify prevalence of postnatal depression in Brazil. | 14 |  |  |  | Brazil | Ranged from 2 weeks and 6 months postnatal. | Majority of studies used EPDS. | The prevalence of postnatal depression was about 30-40% among studies conducted at Family Health Program basic health units among underprivileged populations. The prevalence of postnatal depression was 20% among studies based on large samples of population and tertiary hospital units. |
| 24 | Mahendran | 2019 | Prevalence of antenatal depression in South Asia: a systematic review and meta-analysis. | To examine the prevalence of antenatal depression in South Asia. | 33 | 25 cross-sectional, 7 prospective cohort design. | Total: 13087 pregnant women Sample size ranged from 45 to 1400. | Recruitment from both health facilities and the community. | South Asia: Pakistan, India, Bangladesh, Sri Lanka, Maldives, Nepal. | Pregnancy trimesters 1, 2 or 3. | EPDS, HADS, AKUADS, K-10, MADRS, HDRS, CES-D, PHQ-9, DASS-42 | South Asian countries: antenatal depression pooled prevalence was 24.3% (95% CI: 19.03-30.47). High degree of heterogeneity (I2 = 97.66%) and publication bias (p = 0.668) among studies. Lower prevalence rates: India (17.74%, 95% CI: 11.19-26.96) and Sri Lanka (12.95%, 95% CI: 8.29-19.68). Higher prevalence rates: Pakistan (32.2%, 95% CI: 23.11-42.87) and Nepal (50%, 95% CI: 35.64-64.36). |
| 25 | Mersha | 2018 | Prevalence and Associated Factors of Perinatal Depression in Ethiopia: A Systematic Review and Meta-Analysis. | To identify prevalence and risk factors of perinatal depression in Ethiopia. | 8 | Cross-sectional | Total participants: 4624 mothers. Sample size ranged from 196 to 1311. | 4 community-based, 4 health facility-based. | Ethiopia. | Ranged from ante to postnatal. | EPDS, BDI, WHO, SRQ-20, PHQ-9 | Ethiopia: perinatal depression pooled prevalence was 25.8% (95%CI: 24.6-27.1%). Risk factors: previous history of depression (RR:3.78; 95% CI: 2.18-6.57; I2 = 41.6%), low socioeconomic status (RR: 4.67; 95% CI: 2.89-7.53; I2 = 0%), living apart from spouse (RR: 3.76; 95% CI: 1.96-7.38; I2 = 36.4%), obstetric complications in previous or current pregnancy (RR: 2.74; 95% CI: 1.48-5.06; I2 = 67.7%), unplanned pregnancy (RR: 2.73; 95% CI: 2.11-3.53; I2 = 0%). |
| 26 | Mu | 2019 | Postpartum depressive mood (PDM) among Chinese women: a meta-analysis. | To examine to the prevalence of PDM in China. | 26 | cohort studies, cross-sectional studies. | Total: 7618 women. | Not reported. | China | Ranged from 0–6 months postnatal. | EPDS, SDS, GHQ-12, HAD | Postnatal depression prevalence was 21% (95% CI: 17–25%). It was at its highest rate 0-1.5 months after delivery, but this decreased to 10.4% (95% CI 9.7–11.1%, P < 0.001) after publication bias was modified. |
| 27 | Mukherjee | 2014 | Mental health issues among pregnant women in correctional facilities: a systematic review. | To examine the prevalence and factors of mental health issues among pregnant inmates in correctional facilities, such as: jails, prisons, or any other secure place where convicts are conﬁned. | 11 | 1 mixed-methods, 10 cohort or cross-sectional studies. | Sample size ranged from 25 to 1,213. | Inmates | USA | During pregnancy. | BDI, CES-D, STAI-S | 80% of pregnant inmates in their last trimester had experienced depression. Most stated that absence of satisfactory medical care, isolation, stress, anxiety, maternal role transition, and parenting worries were the most contributory factors. |
| 28 | Mukherjee | 2016 | Racial/ethnic disparities in antenatal depression in the United States: A systematic review. | To identify racial/ethnic disparities in the prevalence and correlates of antenatal depression in the USA. | 41 | 13 cross-sectional, 21 longitudinal studies. | Sample size varied from 56 to 19,219. | Some studies had specific groups of women, e.g. smokers; smoking quitters; low-income inner city women, minority women. Most were recruited from obstetric care at Federally Qualified Health Centers (FQHCs), Community Obstetric/Gynecological Clinics, University-based Hospitals. | USA | During pregnancy | CES-D, EPDS, BHQ, PHQ, SCID, (DIS)-IV. | Antenatal depression: prevalence ranged from 10-30% in USA; higher among non-Hispanic blacks (NHB) and Hispanics, c.f. non-Hispanic whites (NHWs). Higher rates of depression were found among employed NHBs compared to NHWs; whereas racial difference was not observed among unemployed women. Higher parity, complex stress, and lower self-esteem associated significantly with depression among NHBs. Low satisfaction, low social support, and higher stress were seen as higher risk factors for depression among NHWs and Hispanics respectively. |
| 29 | Nilaweera | 2014 | Prevalence, nature and determinants of postpartum mental health problems among women who have migrated from South Asian to high-income countries: a systematic review of the evidence. | To identify the prevalence & factors of postnatal mental health issues among South Asian women who have migrated to High-Income Countries. | 15 | 10 x quantitative (cohort & cross-sectional) 5 x qualitative studies. | Total sample size was 102,427 (quantitative) Total sample size 84 x qualitative studies (approx.32 x S. Asian. | Not reported. | USA, Canada, Norway, Australia, UK. | Birth to 1 year postpartum. | PPDS DSM-IV CES-D EPDS. | Prevalence: Widely diverse re significant symptoms of postnatal depression (1.9–52%). Most common rate ranged from 5 to 20%. 2 x increased high risk to overseas born women with a S. Asian group (OR 1.8–2.5). Risk Factors (most common): social isolation low quality of relationship with the partner, difficulties with accessing health care e.g lacking English language proficiency, unfamiliarity/ lack of attention to local mental health services, attitude to role of health care providers cultural factors. |
| 30 | Özcan | 2017 | Postpartum depression prevalence and risk factors in Turkey: a systematic review and meta-analysis. | To examine the prevalence of and risk factors for postnatal depression in Turkey. | 52 | 14 follow-up, 38 descriptive and cross-sectional. | Sample size ranged from 41 to 2514. | 1 study included primiparous women, 51 the postnatal period (1 to 24 months). | Turkey | Pregnancy to 18 months postnatal. | EPDS, BDS, BAI, SCL-90-R, STAI, GHQ, SCDI-I | Prevalence of postnatal depression in Turkey was 23.8% (95% CI: 21.6–26.1%), rate values varied between 5-61.8% among the studies. Prevalence in developed cities was 21.2% (95% CI: 17.2–25.9%). Prevalence in developing cities was higher, 25% (95% CI: 22.4–27.8%). Major risk factors of postnatal depression: economic status and the work status of the spouse, unplanned pregnancy, stressful life events during gestation, health issues in the child, personal/family history of psychiatric illness, conflicts with family/spouse, lack of social support |
| 31 | Ren | 2014 | Mental disorders of pregnant and postpartum women after earthquakes: a systematic review. | To identify the psychological impact of earthquakes on perinatal women. | 8 | Cross-sectional studies | Sample size ranged from 29 to 1545. | Post earthquake pregnant/postnatal women. | Not reported. | Ranged from pregnancy to postnatal. | CHQ, EPDS | In women who had not experienced an earthquake, the prevalence of antenatal depression ranged from 7-13%, c.f postnatal depression at 3.5-63.3%. In those who had experienced an earthquake, antenatal prevalence of depression ranged from 7.1-40.8%. The postnatal prevalence of depression was found to range from 11.6-29%, especially if the earthquake had occurred during gestation. Contributory factors to perinatal depression: stressful events, family relationships, economic status and work status, social support, the timing of the earthquake during gestation, history of birth and baby-rearing. |
| 32 | Sawyer | 2010 | Pre-and postnatal psychological wellbeing in Africa: a systematic review. | To examine the prevalence and risk factors of maternal mental health disorders in African women living in Africa. | 35 | 19 x cross-sectional 11 x longitudinal 5 x case–control studies. | Total 10,880 participants. Sample sizes ranged from 27 to 1723. | Recruitment from antenatal and postnatal health clinics; the community or both. | Africa (Nigeria, South Africa, Uganda, Ethiopia, Morocco, The Gambia, Zimbabwe, Malawi). | Ranged from ante to postnatal. | SCI EPDS HADS BDI SRDS PDQ | Antenatal Depression was the most commonly found disorder in the studies, with prevalence rates from 4.3% to 17.4% & a mean prevalence of 11.3% (95% CI 9.5%–13.1%).Postnatal Depression rates ranged from 3.2% to 48.0%; with a mean prevalence of 18.3% (95% CI 17.6%–19.1%). Lack of support and marital/family conflict were associated with perinatal mental health disorders. Sociodemographic & obstetric factors were inconclusively related to poorer mental health. |
| 33 | Schmied | 2013 | Maternal mental health in Australia and New Zealand: A review of longitudinal studies. | To examine factors that impact on the mental health of Australian and New Zealand women during pregnancy and the postnatal period. | 8 | longitudinal studies | Not reported | Not reported | Australia and New Zealand | Ranged from antenatal to postnatal | EPDS, DSM-1V, MINI | Prevalence of moderate to severe depression for at least a small part of the postnatal year ranged from 10-20%. 2 strong associated factors were: a history of previous depression/anxiety, deprived partner, relationship depression |
| 34 | Shorey | 2018 | Prevalence and incidence of postpartum depression among healthy mothers: a systematic review and meta-analysis. | To examine the prevalence and incidence of depression after the birth among healthy mothers without any previous history of depression. | 58 | 30 prospective cohort studies, 26 cross-sectional studies, 2 case-control studies. | Total: 37, 294 | Not reported. | Asia, Europe, the Middle East, North America, South America, Australia, Africa. | Ranged from pregnancy to 1 year postpartum. | Clinical interviews, EPDS, BDI, IDD, DASS, PHQ-9, HDRS | The incidence of postnatal depression was 12% (95% CI: 0.04–0.20) c.f. the global prevalence of postnatal depression at 17% (95% CI: 0.15–0.20) among healthy mothers who had not experienced any previous history of depression. Prevalence rates were statistically significant (P <0.001) among diverse geographical regions. The highest prevalence was found in the Middle East (26%, 95% CI: 0.13–0.39); followed by Australia (21%, 95% CI: 0.16–0.25), South America (19%, 95% CI: 0.18–0.21), Asia (16%, 95% CI: 0.13–0.20), North America (16%, 95% CI: 0.11–0.20), Africa (11%, 95% CI: 0.05–0.17) Europe (8%, 95% CI: 0.05–0.11) had the lowest prevalence. There was no statistical variance in rate between diverse screening time points, however prevalence was found to be increased beyond 6 months after the birth. |
| 35 | Sowa, N. A., | 2015 | Perinatal depression in HIV-infected African women: a systematic review. | To examine prevalence and incidence of antenatal and postnatal depression in human immunodeficiency virus (HIV)–infected African women. | 22 | Cross-sectional studies x secondary analysis cohort studies. | Sample size ranged from 70 to 1922. | (HIV)–infected African women recruited mainly from urban populations & antenatal clinics. | Africa. | Ranged from pregnancy to 1 year postpartum. | ICD-10 DSM-IV EPDS CES-D subsets of HSCL-8 HSCL-15 SRQ-20 SSQ. | 2 x diagnostically confirmed antenatal depression 9 x suspected antenatal depression prevalence (cases where screening tools identified depression, unconfirmed by diagnostic measure). 2 x studies diagnostically confirmed postnatal depression.10 x suspected postnatal depression prevalence. Mean prevalence of ante & postnatal depression was 23.4% & 22.5% respectively, among HIV infected women. Mean prevalence of suspected ante & postnatal depression was 43.5% & 31.1% respectively. |
| 36 | Takegata | 2017 | Cross-national differences in psychosocial factors of perinatal depression: A systematic review of India and Japan. | To compare factors relating to perinatal depression in India and Japan. | 50 | Cross-sectional studies, longitudinal studies, qualitative studies | Sample size ranged from 54 to 5801. | Not reported. | India, Japan. | Ranged from pregnancy to 1 year postpartum. | CES-D, BDI, PHQ, DSM-IV, SCID, EPDS, KPDS | In India, the use of SRQ indicated a prevalence of 16-33% antenatal and 7-65% postnatal depression. In Japan, SRQ indicated a prevalence of 6–41% antenatal and 8–29% postnatal depression. The prevalence of both using structured interviews ranged from 16% in India to 19% in Japan. A rate of 5% was found for both antenatal and postnatal depression. Shared risk factors in India and Japan: vulnerable personality, history of abuse, age, marital conflict, lower socio-demographic status. India: low socioeconomic status, living only with the husband, pregnancy not welcomed by the husband, bearing a female child, conflicts with in-laws. Japan: infertility treatment, conflict with work–life balance, conflicts with biological mother or in-laws, concerns around social associations with the other mother’s friends. |
| 37 | Underwood | 2016 | A review of longitudinal studies on antenatal and postnatal depression. | To identify recent prevalence of depression during pregnancy and after childbirth. | 16 | Longitudinal studies | Sample size ranged from 525 to 8323. | Young, single women of low socioeconomic status were under-represented. | Iran, Italy, Canada, Turkey, Norway, UK, USA, Germany, Sweden, Thailand, Australia, Brazil, France, New Zealand. | Ranged from pregnancy to 12 months postnatal | DSM-IV, SCID-I, EPDS, CES-D 7 item subscale: DSSI | Mean prevalence rate of ante- and postnatal depression were 17% and 13%, respectively. Longitudinal studies found that about 39% of women who had experienced antenatal depression went on to have postnatal depression too. 47% of women with postnatal depression were found to have experienced antenatal depression. 7% of women experienced significant antenatal depressive symptoms that continued in the postnatal period. Greatest risk factors emerging were a past history of depression or other mental health illness. |
| 38 | Upadhyay | 2017 | Postpartum depression in India: a systematic review and meta-analysis. | To identify the prevalence and risk factors of postnatal depression in Indian mothers. | 38 | Cohort studies, cross-sectional studies, 1 RCT | Total: 20,043 mothers | Mean age of the mothers was ≤ 25 years. Women were recruited from urban and rural hospitals. | India | Ranged from within 2 weeks to 1 year postpartum. | EPDS, BDI, MINI, DSM-IV, SCID | Overall pooled prevalence of postnatal depression was 22% (95% CI: 19–25). The highest prevalence was found in the southern regions (26%; 95% CI: 19–32) and the lowest in the northern regions (15%; 95% CI: 10–21). Pooled prevalence was 19% (95% CI: 17–22) after excluding 8 studies reporting postnatal depression within 2 weeks of the birth. Differences in prevalence by mothers' age, geographical location and study setting were non-significant.Risk factors included: financial problems, domestic violence, past history of psychiatric problems, marital conflict, poor support from husband having a female baby. |
| 39 | Veisani | 2013 | Trends of postpartum depression in iran: a systematic review and meta-analysis. | To identify prevalence of postnatal depression in Iranian women. | 41 | Not reported. | 21,907 women. | Not reported. | Iran | Ranged from 2 to 52 weeks postpartum. | EPDS, BDI | Pooled prevalence of postnatal depression was 25% (95% CI: 22.7–27.9%). EPDS: Iran's prevalence of postnatal depression was 24.3% (95% CI: 21.0–27.7), BDI prevalence: 25.3% (95% CI: 22.7–27.9). Subgroup prevalence: unwanted delivery: 43.4% (35.6–51.1) illiterate 31.6% (18.1–45.0), housewives: 30.7% (25.2–36.3), history of depression: 45.2% (35.4–53.1). |
| 40 | Veisani | 2012 | Prevalence of postpartum depression in Iran-A systematic review and meta-analysis. | To identify prevalence of postnatal depression in Iranian women. | 15 |  | 11665 Sample size: average of 777 |  | Iran | Ranged from pregnancy to 1 year postpartum. | EPDS, BDI. | In Iran, the pooled prevalence of postnatal depression was 28.7% (CI 95%: 24.9-32.5). EPDS had a prevalence of 26.9% (CI 95%: 22.3-31.6) while BDI had a prevalence of 30.5% (CI 95%: 21.4-39.6). The west and south-eastern border areas of Iran had the greatest rate of postnatal depression, whereas the centre region had the lowest rate. Women with a history of depression (39.6%, CI 95%: 36.6-73.3), employed women (33.5%, CI 95%: 12.1-54.8), housewives (29%, CI 95%: 14.5-43.5), women with wanted pregnancies (40.3%, CI 95%: 26.6-54.1), women with unwanted pregnancies (50.5%, CI 95%: 34.8-66.3) were more likely to have postnatal depression. |
| 41 | Vigod | 2010 | Prevalence and risk factors for postpartum depression among women with preterm and low‐birth‐weight infants: a systematic review. | To examine the prevalence and risk factors for postnatal depression among women with preterm infants. | 26 | Not reported. | Total: 2392 mothers of preterm infants. | Women with preterm infants. | Not reported. | Ranged from 2 to 52 weeks postpartum. | EPDS BDI CES-D | Mothers with significantly preterm or very low birth weight infants had a higher risk of developing postnatal depression in the 1st year postpartum Rates of early postnatal depression were as high as 40% among these women. Sustained depression was related to: earlier gestational age, lower birth weight, infant with illness or disability poor or lacking social support. |
| 42 | Villegas | 2011 | Postpartum depression among rural women from developed and developing countries: a systematic review. | To examine the prevalence of and risk factors of postnatal depression in rural communities within developed and developing countries. | 19 | 8 cross‐sectional, 7 longitudinal studies | Sample sizes ranged from 16 to 6,627 rural women. | Rural mothers in 1st year postpartum. Age: 14 to 42 years. | 8 developed countries, 11 developing countries. | Ranged from 2 weeks to 12 months postpartum. | EPDS, BDI, CES-D, PDSS, SCID, PRIME‐MD, CIS‐R | Total prevalence of postnatal depression among rural women was 27.0% (95% CI, 18.8‐37.2%). Developing countries had greater rates of postnatal depression among rural women (31.3%; 95% CI, 21.3–43.5%) than developed ones (21.5%; 95% CI, 10.9–38.0%). Numerous shared risk factors: single status, low socioeconomic status, past history of psychiatric problems, antenatal depression, stressful events, history of abuse, poor or lacking social support, rural women in developing countries, having 2-5 more young children, unemployed/illiterate husband years of marriage, lack of awareness of taking care of infants, difficulties with in‐laws infant’s gender. |
| 43 | Vliegen | 2014 | The course of postpartum depression: a review of longitudinal studies. | To examine the course of postnatal depression and its risk factors. | 23 | 23 longitudinal studies. | Sample sizes ranged from 14 to 552 mothers. | Mothers recruited from community and clinical sample. | Not reported. | Ranged from 1 week to 12 month postpartum. | EPDS, CES-D, GHQ, SCID, BDI, BDI-II, SPI | Prevalence of postnatal depression was 30% among mothers in community and 50% in clinical settings. The severity of the depression reduced at various time points after the birth, not always to a non-depressed level, not always statistically significant. Subgroups of depressed mothers: e.g. chronically depressed mothers with sustained clinically elevated depressive symptoms, remitted mothers whose postnatal depression was major and acute initially but then no longer found at their follow-up. Common risk factors were: lower quality of partner relationship, and of parenting or maternal care, stressful life events, family conflict, lack of social support, history of depression or mental disorders, history of sexual abuse, personality factors, e.g. immature defence style and excessive self-criticism. |
| 44 | Woody | 2017 | A systematic review and meta-regression of the prevalence and incidence of perinatal depression. | To identify global prevalence and incidence of perinatal depression. | 96 | Not reported. | Sample size ranged from <100 to 5000+. | Not reported. | 3 Low Income Countries, 33 Middle Income Countries, 60 High Income Countries. | Ranged from pregnancy to specific different points of the postnatal month. | 37 diagnostic instruments, 74 symptom scales e.g. EPDS. | Meta-regression found 31.1% of the inconsistency in prevalence described among studies. Prevalence rates based on using symptom scale measures were significantly higher than prevalence based on using diagnostic tools (OR: 1.6, 95% CI: 1.3–2.0). Prevalence of perinatal depression was higher among women from Low and Middle-Income Countries (13.1%, 95% CI: 12.2–14.1) than among women from High Income or developed countries (11.4%, 95% CI: 10.8–12.1). Pooled prevalence was found to be significantly higher among studies of Low and Middle Income countries than those from High Income Countries in both antenatal (19.2%, 95% CI: 18.0–20.5 vs. 9.2%, 95% CI: 8.4–10.0, respectively) and postnatal periods (18.7%, 95% CI: 17.8–19.7 vs. 9.5%, 95% CI: 8.9–10.1) respectively. The review concludes that the global pooled prevalence of perinatal depression was 11.9% with (95% CI: 11.4–12.5). |
| 45 | Zegeye | 2018 | Prevalence and determinants of antenatal depression among pregnant women in Ethiopia: a systematic review and meta-analysis. | To examine the prevalence of antenatal depression and its risk factors among pregnant women in Ethiopia. | 10 | 9 cross-sectional studies, 1 prospective cohort. | Total: 4983 Ethiopian women. Sample size ranged from 187 to 1311. | Women recruited from public health centres or hospitals. | Ethiopia | During pregnancy | EPDS, BDI, PHQ-9 | In Ethiopia, the general prevalence of antenatal depression was found to be about 24.2% (95% CI: 19.8, 28.6). The subgroup analysis found that the highest and lowest prevalence of antenatal depression were 26.9% (21.9–32.1) in Addis Ababa and 17.25% (95% CI: 6.34, 28.17) in Amhara. Risk factors included: previous history of abortion (OR: 3.0, 95% CI: 2.1, 4.4), marital conflict (OR: 7.2; 95% CI: 2.7, 19.0), absence of social support from husband (OR: 3.2: 95% CI: 1.2, 8.9), previous history of pregnancy complication (OR: 3.2: 95% CI: 1.8, 5.8). |
| 46 | Atif | 2021 | Perinatal depression in Pakistan: A systematic review and meta- analysis | To examine the prevalence perinatal depression and its risk factors among Pakistani mothers. | 43 | 30 cross-sectional studies, 10 prospective cohort studies, 1 case-control study, 1 RCT study, 1 quasi-experimental study | Total: 17 544 women. Sample size ranged from 75 to 1369. | Women recruited from hospitals or community-based. The mean age of the mothers was 26.9 years | Pakistan | Ranged from ante- to postnatal. | EPDS, CES-D, AKUADS, HADS, GADS, DASS-21, BDI, SDS, SSDS, Pitt's questionnaire for puerperal depression, HDRS, CIS | In Pakistan, the pooled prevalence of antenatal depression was 37% (95% CI: 30-44), while postnatal depression was 30% (95% CI: 25-36).The most common risk factors for perinatal depression were intimate partner violence and poor relationship with spouse, low-income level and unintended pregnancy. |
| 47 | Yin | 2021 | Prevalence and associated factors of antenatal depression: Systematic reviews and meta-analyses | To examine the prevalence of antenatal depression and its risk factors. | 173 | Cross-sectional studies, prospective cohort studies. | Total: 197,047 women. Sample size ranged from 21 to 35,374. | The majority of studies recruited participants during the third trimester. | 50 countries across six continents, USA (n = 39), Australia (n = 11), Brazil (n = 11), China (n = 10) | During pregnancy | EPDS, CES-D, BDI, PHQ-9, SCI/CD | In 173 studies, the prevalence rate of any antenatal depression was 20.7% (95% CI: 19.4–21.9%, P = 0.000, I2 = 98.4%), while in 72 studies, the prevalence rate of major antenatal depression was 15.0% (95% CI: 13.6–16.3%, P = 0.000, I2 = 97.8%). |
| 48 | Endomba | 2021 | Perinatal depressive disorder prevalence in Africa: A systematic review and Bayesian analysis | To examine the prevalence of perinatal depression in Africa | 154 | Cross-sectional studies, prospective cohort. | Total: 113,147 women  Sample size ranged from 23 to 4680. | Women recruited from public health centres or hospitals. | Africa. | Ranged from ante- to postnatal. | EPDS (Majority), HADS, MINI, PHQ-9, SCID, BDI, K-10, DSM-IV, PRQ, SRDS, SRQ, CESD, PDQ, DASS-42 | The prevalence of antenatal depression was 22.8% (95% Credible interval [CrI]: 21.5–24.1) among women with no specified disease and 31.9% (95% CrI: 30.2–33.6) among those with HIV. The prevalence of postnatal depression was 21.2% (95% CrI: 20.0–22.5) among women with no specified disease, 30.0% (95% CrI: 28.2–31.8) with HIV, and 44.6% (95% CrI: 35.9–53.8) with poor pregnancy outcomes. |
| 49 | Shorey | 2021 | Anxiety and depressive symptoms of women in the perinatal period during the COVID-19 pandemic: A systematic review and meta-analysis | To examine the prevalence of perinatal anxiety and depressive symptoms among pregnant women and postnatal mothers during the COVID-19 period. | 26 | 17 cross-sectional studies, 9 case control studies. | Total: 24,040 women. | Women aged from 18 to 50 years. Women in the perinatal period during COVID-19 pandemic. | Asia (n = 12), Europe (n = 8), North America (n = 6). | Ranged from ante to 18 months postnatal. | EPDS, BDI, HADS, PHQ-2 | The prevalence rates for antenatal depressive symptoms and postnatal depressive symptoms were 27% (95% CI: 0.20–0.33) and 17% (95% CI: 0.10–0.24), respectively. |
| 50 | Liu | 2021 | Prevalence and Risk Factors of Postpartum Depression in Women: A Systematic Review and Meta-analysis | To examine the prevalence of postnatal depression and its risk factors. | 33 | 4 case control studies, 29 cohort studies | Total: 137,866 women. Sample size ranged from 162 to 90,194. | Women aged from 18 to 50 years. | China (n = 6), United States (n = 3), Iran (n = 3), Japan (n = 3), Singapore (n = 3), Australia (n = 2), Brazil (n = 2), Thailand (n=1), Finland (n=1), Maldives (n=1), Tanzania (n=1), Czech Republic (n=1), Kenya (n=1), Sweden (n=1), Malaysia (n=1), Denmark (n=1), France (n=1), Central Europe (n=1) | Ranged from pregnancy to 12 month postpartum. | EPDS | The overall prevalence of postnatal depression was 14.0% (95% CI: 12.0%–15.0%). The prevalence of postnatal depression varies by nation (ranging from 5.0-26.32%). The risk factors of postnatal depression include: gestational diabetes mellitus (OR = 2.71, 95% CI: 1.78–4.14, I2 = 0.0%), antenatal depression (OR = 2.40, 95% CI: 1.96–2.93, I2 = 96.7%), giving birth to boys (OR = 1.62; 95% CI: 1.28–2.05; I2 = 0.0%), history of depression during pregnancy (OR = 4.82, 95% CI: 1.32–17.54, I2 = 74.9%), history of depression (OR = 3.09, 95% CI: 1.62–5.93, I2 = 86.5%) and epidural anaesthesia during delivery (OR = .81, 95% CI: .13–4.87, I2 = 90.1%). |
| 51 | Alshikh Ahmad | 2021 | Prevalence and risk factors of postpartum depression in the Middle East: a systematic review and meta–analysis | To examine the prevalence of postnatal depression and its risk factors among among the women in Middle East countries. | 15 | 9 cross-sectional studies, 6 cohort studies | Total: 6683 women. Sample size ranged from 56 to 1379. | Women aged from 15 to 48 years. | Middle East countries, such as Iran. | Ranged from 2nd trimester to 8 months postnatal. | EPDS, BDI (only one study) | The overall prevalence of postnatal depression in Middle Eastern mothers was very high 27% (95% CI: 0.19–0.35). The common risk factors included: poor economic, pregnancy related complications, low education, unplanned pregnancy, housewife, lack of social support and feeding by formula. |
| 52 | Corcoran | 2022 | Prevalence of depression during pregnancy and postpartum periods in low-income women in developed countries | To examine the prevalence of perinatal depression in women living in low-income in developed countries. | 64 | Not mentioned | Total: 218,035 women. | Women of varying ethnicities. | Most of the studies (82%) were conducted in the United States. | Ranged from ante- to postnatal. | EPDS, BDI, PHQ, CESD, DSM | The prevalence of perinatal depression was 33.82% among low-income women. |
| 53 | Safi‐Keykaleh | 2022 | Prevalence of postpartum depression in women amid the COVID-19 pandemic: A systematic review and meta-analysis | To examine the prevalence of postnatal depression during COVID-19. | 24 | 18 cross-sectional studies, 5 cohort studies, 2 case control studies | Sample size ranged from 34 to 5134. | Postpartum women during the COVID-19 pandemic | Ireland, Norway, Switzerland, Netherlands, UK, China, Hong Kong, Turkey, Serbia, Poland, Japan, Brazil, Spanish, Italy, Israel, Mexico, Belgium, Argentina, Saudi Arabia. | During the postnatal period. | EPDS, PDSS-SF | The prevalence of postnatal depression was 12% (95% CI = 0.07–17, I2 = 97%), 27% (95% CI = 15–39, I2 = 99%), 44% (95% CI = 40–49, I2 = 0.0%), 27% (95% CI = 0.06–49, I2 = 97.4%), 28% (95% CI = 18–39, I2 = 98.5%), 37% (95% CI = 32–42), 28% (95% CI = 23–33, I2 = 98.5%) based on EPDS ≥9, EPDS ≥10, EPDS ≥11, EPDS ≥12, EPDS ≥13, PDSS-SF ≥17 respectively. |
| 54 | Chen | 2022 | Prevalence and Risk Factors Associated with Postpartum Depression during the COVID-19 Pandemic: A Literature Review and Meta-Analysis | To evaluate the COVID-19 pandemic's impact on the prevalence of postnatal depression and to determine risk factors for postnatal depression during the COVID-19 pandemic. | 8 | 8 cross-sectional studies | Total: 6480 postpartum women sample size ranged from 50 to 592 | Postpartum women during the COVID-19 pandemic | Developed countries, such as the UK, countries in Europe, and Canada. | Women up to 18 months postnatal period. | EPDS | The overall prevalence of postnatal depression was 34% during the COVID-19 pandemic. Risk factors for postnatal depression included: socio-demographic and clinical features (maternal age, job status, marital status, pregnancy intention and smoking), lack of supports, stress and anxiety, and factors related the COVID-19 (fears of infection). |
