# Supplementary material for: Prevalence and correlates of perinatal depression
Source: Soc Psychiatry Psychiatr Epidemiol. 2023 Jan 16;58(11):1581–90. doi: 10.1007/s00127-022-02386-9 (PMC9842219; doi:10.1007/s00127-022-02386-9)
Supplement: Supplementary file 3 — Supplementary file3 (DOCX 57 KB) [file 127_2022_2386_MOESM3_ESM.docx]

Table 2: Characteristics of the included systematic reviews related to the risk factors associated with developing perinatal depression.

| **No.** | **First author** | **Publication year** | **Title of Review** | **Review Objective or Aims** | **Number of Included Studies** | **Type of Studies** | **Sample Size** | **Characteristics of Population** | **Countries** | **Time of Measurements/Timepoints** | **Type of Depression Measurements** | **Key Findings** |
| --- | --- | --- | --- | --- | --- | --- | --- | --- | --- | --- | --- | --- |
| 1 | Arafa | 2019 | Gestational diabetes and risk of postpartum depressive symptoms: A meta-analysis of cohort studies. | To examine the association between gestational diabetes and risk of postnatal depression by a using a meta-analysis of cohort studies. | 10 | 10 cohort studies: 5 prospective cohort studies and 5 retrospective. | Total sample size of 2,000,002. | Examine the rate of depression in women with gestational diabetes compared with those without it. | USA, Europe, Canada, Iran. | Depression was measured between 1-12 months postnatal. | EDPS, ICD, Self Reported | A significant association between gestational diabetes and increasing risk of postnatal depression (pooled RR = 1.32, 95% CI: 1.09–1.60), compared with women without gestational diabetes. Heterogeneity was high among the studies (I2 = 69.1%, P for heterogeneity = 0.001). Study design modified the association, and the pooled RRs for prospective was .95 (95% CI:1.48, 2.57), for retrospective cohort studies was 1 and 1.16 (95% CI: 1.12, 1.21). No heterogeneity was found on both retrospective cohort studies (I2 = 0%, P for heterogeneity = 0.55) and prospective cohort studies (I2 = 22.1%, P for heterogeneity = 0.27). |
| 2 | eCouto | 2015 | Postpartum depression: A systematic review of the genetics involved. | To identify which genes and polymorphisms are related to postnatal depression and to explain if these genes are same as related to major depression. | 20 | Not reported. | Not reported. | Not reported. | Sweden, USA, Germany, China (Taiwan), Spain, Canada, UK, Brazil, Israel and Netherlands. | Not reported. | EPDS, CES-D, SADS, SCI for DSM-IV; HRSD-17, CIDI-SF, MADRS | The focus was on serotonin transporters, most commonly 5HTT and 5HTTLPR. Findings: 5HTTLPR was associated with postnatal depression in the studies. Perinatal depression was associated with TPH1 and TPH2 polymorphisms, atechol-O-methyl transferase (COMT) and Monoamine Oxidase (MAO) polymorphisms, and they are therefore also considered as risk factors. |
| 3 | Halim, N., | 2018 | Intimate partner violence during pregnancy and perinatal mental disorders in Low and Lower Middle Income Countries: A systematic review of the literature. | To systematically examine the association between Intimate partner violence (IPV) experiences during pregnancy and developing perinatal mental disorders in women living in LLMIC. | 24 | 12 cross-sectional, 12 prospective-cohort studies. | Sample size ranged from 61 to 1369 participants selected randomly/non-randomly from clinics or communities. | Age range: 15-30 years; Gestational range: 12-34 weeks; Postnatal range: 6 weeks - 6 months. 15% of Zimbabwean women in the study were HIV-positive. | LMIC (South Asia, East Asia, Pacific Middle East, North Africa, Sub-Saharan Africa). | Ranged from 6 weeks to 6 months postpartum | EPDS, BDI, GHQ, SCI | The prevalence of IPV ranged from: physical IPV 2-35%; sexual IPV 9-40% psychological IPV 22-65%. The prevalence of antenatal depression ranged from 15-65% with suicidal ideation in pregnancy ranging from 5-11%. The prevalence of postnatal depression ranged from 5-35%. With suicidal ideation ranging from 2-22% postnatal. Women who had experienced IPV had 1.69-3.76 and 1.46-7.04 higher chances of developing both antenatal and postnatal depression than women who had not, depending on country, type and severity of IPV. |
| 4 | Han | 2014 | Maternal and foetal outcomes of intimate partner violence associated with pregnancy in the Latin American and Caribbean region (LAC). | To identify the risk factors, prevalence, and adverse maternal outcomes of IPV‐P in LAC. | 31 | Cross-sectional prospective-cohort studies, case control studies. | Not reported. | Not reported. | LAC countries: Peru; Bolivia; Colombia; Brazil; Mexico; Guatemala; Costa Rica; Nicaragua; Haiti; Dominican Republic. | Not reported. | Not reported. | Prevalence rates of IPV among LAC women ranged from 3% to 44%. IPV during pregnancy was significantly associated with unintended pregnancies and adverse maternal factors such as depression, and pregnancy‐related symptom distress. The prevalence of depression in women with experience of IPV during pregnancy was estimated to be from 18.1-25.8%. |
| 5 | James-Hawkins | 2019 | Human and economic resources for empowerment and pregnancy-related mental health in the Arab Middle East.a systematic review. | To examine the influence of human and economic resources for women’s empowerment regarding their pre- and postnatal mental health. | 24 | Not reported. | Sample sizes ranged from 79 to 1659 women, with an average of 483 participants across studies. | Primary data were collected from participants via their clinics. | Arabic countries such as Egypt, KSA, Oman | All 3 trimesters of pregnancy to 1 year postnatal | EPDS, MINI, DASS-21, BDI, HADS, DDI | There is a negative relationship between perinatal education and depression; there are inconsistent and unclear relationships between employment and depression. In the Arabic world, there is link between financial stress and perinatal depression. High financial stress has been associated with Arabic women because along with caring for children and their household duties they may also be constrained from going to work by their husbands resulting in feelings of helplessness towards finance and their family. |
| 6 | Lancaster | 2010 | Risk factors for depressive symptoms during pregnancy: a systematic review. | To examine risk factors for antenatal depression that can be assessed in routine obstetric settings. | 57 | Not reported. | Sample sizes ranged from 39 to 6642. | Age varied from 20 to 33 years. | USA (most studies), Australia, Norway, Germany, Canada, Netherlands, Sweden, UK, Finland. | During pregnancy. | EPDS, ADS-K (German version of CESD), BDI, CESD, DACL, HADS, HSCL, MINI, PHQ, SADS, SCID | Most common risk factors in antenatal depression: maternal anxiety, stressful life events, history of past depression, low/lacking social support, unintended pregnancy, Medicaid insurance, domestic violence, lower income, lower educational attainment, smoking or alcohol use or illicit drug use, single status, poor relationship quality. Multivariate analyses: Significant: Stressful life events, lack of social support, and domestic violence. |
| 7 | Lara-Cinisomo | 2016 | A biopsychosocial conceptual framework of postpartum depression risk in immigrant and US-born Latina mothers in the United States. | To examine a conceptual framework that identifies risk factors of postnatal depression among immigrant and U.S.-born Latinas in the United States of America (USA). | 16 | Secondary data analysis experimental cross-sectional longitudinal studies. | Sample size ranged from 12 to 5,380. | Immigrant and US-born Latinas in the USA. | USA | During pregnancy and postpartum. | CES-D, BDI, EPDS, PDSS, DSM-IV, Hamilton Rating Scale | Postnatal depression of Latina women in the US appeared to be associated with contextual and cultural psychosocial factors (e.g. poverty and childhood/adulthood traumatic experiences and acculturative stress). Physical: These cultural and contextual stressors may influence neuroendocrine functions. e.g. dysregulation of the hypothalamic-pituitary-adrenal axis response system and decreased levels of the oxytocin is negatively affected by stress and trauma may create risk of developing postnatal depression. |
| 8 | Lawson | 2015 | The relationship between sleep and postpartum mental disorders: A systematic review. | To examine the relationship between sleep and postnatal mental disorders. | 31 | 1 RCT, 2 chart reviews, 28 cohort studies/cross-sectional designs | Sample sizes varied greatly from 16 more than 4000. | Not reported. | USA (most studies). Remainder from Norway, Iran, Portugal, Canada, United Kingdom, Australia, Belgium, Taiwan. | Not reported. | EPDS, BDI, CES-D, PHQ-9, MINI, HADS, SCID/DSM-IV | Development of postnatal depression was associated with self-reported sleep distribution during the antenatal and postnatal periods. |
| 9 | Petzoldt | 2018 | Systematic review on maternal depression versus anxiety in relation to excessive infant crying. | To examine the role of maternal depression and anxiety considering them as preceding, concurrent and subsequent conditions of Excessive Crying (EC). | 30 | 20 longitudinal cohort designs, 6 case-control designs, 4 cross-sectional designs. | Sample size ranged from 24 to 7765 mother-infant dyads. | Mean maternal age ranged from 25.2 to 33.7 years (5 did not report on this). | USA, Canada, Australia, Germany, Switzerland, Turkey, Netherlands, Denmark, Sweden, Belgium, Italy, Poland, Spain. | Pregnancy to postnatal. | EPDS, BDI, CES-D, PHQ-2, DSM-IV, SCID-I, BSI | Significant positive association in most studies for perinatal depression as well as for maternal anxiety and EC. Concurrent and subsequent perinatal depression was significantly associated with EC. Simultaneously, preceding perinatal depression was not associated. Perinatal depression can be a risk factor or a consequence of EC. Anxiety temporally recedes illness and can be a predictive risk factor for both subsequent EC and associated perinatal depression. |
| 10 | Recto | 2017 | Psychosocial risk factors for perinatal depression among female adolescents: A systematic review. | To identify psychosocial risk factors for antenatal and postnatal depression among adolescents. | 17 | longitudinal studies intervention studies. | Sample sizes ranged from 62 to 932. | Female adolescents of diverse ethnicities: Primarily non-Hispanic White, Hispanic, & African-American. | Not reported. | Pregnancy to postnatal. | EPDS, CES-D, BDI, PHQ, CDRS-R | Most common factors associated with perinatal depression: lack of social support especially support from the baby's father, perceived stress, past history of depression, history of sexual abuse (e.g. rape, attacks on sexual body parts, child molestation, forced sex) physical violence (e.g. hitting, slapping, beating, pushing, or being exposed to a weapon) Additional factors include: adolescent's perception of her pregnancy, family criticism of pregnancy and parenting, self-efficacy,self-confidence, using drugs or substances, parental stress, public violence, anxiety, African-American ethnicity. |
| 11 | Ross | 2010 | Risk for postpartum depression associated with assisted reproductive technologies and multiple births: a systematic review. | To examine if women who conceive using assisted reproductive technologies (ART) and women with multiple births are associated to increase risk of postnatal depression or not. | 13 | Not reported. | Sample size ranged from 24 to 330. | Women using ART or with multiple births. | Not reported. | Ranged from 2–52 weeks postpartum. | EPDS, CES-D, GHQ | Women who conceive using ART showed little or no increased risk of developing postnatal depression. It was reported by only 1 out of 7 studies who found a significant association. Stronger but inconsistent findings (3 studies) suggest a possible association between multiple births & postnatal depression. |
| 12 | Trujillo | 2018 | A systematic review of the associations between maternal nutritional biomarkers and depression and/or anxiety during pregnancy and postpartum. | To examine the associations between nutritional biomarkers and depression/anxiety during pregnancy and in the first year of the giving birth. | 38 | 27 cohort, 9 cross-sectional 2 case-control studies. | Sample sizes ranged from 18 to 4101. | Not reported. | USA, Asia, Europe & Oceania. | Ranged from pregnancy to 1 year postpartum. | EPDS most commonly used in the studies. | 13 found contradictory or no associations between serum/plasma/erythrocyte fatty acid concentrations & risk of depression or anxiety in the ante & postnatal periods. Alterations in serum cholesterol levels showed a significant inverse association with depression. Most studies examining the serum vitamin D levels found that lower vitamin D levels may be associated significantly with ante & postnatal depression. Plasma tryptophan levels were also found inversely associated with risk of postnatal depression. With very limited evidence, the review found no significant association between vitamin B12/folate/ferritin concentrations & risk of postnatal depression. |
| 13 | Abajobir | 2016 | A systematic review and meta-analysis of the association between unintended pregnancy and perinatal depression. | To examine the association of unintended pregnancy and perinatal depression. | 10 | 5 cross-sectional 4 observational longitudinal 1 randomized clinical trial design. | Sample sizes ranged from 215 to 18,059 participants. | Wide range from pregnancy to 12 months postnatal, from community or hospitals. | Australia, USA, China, Ethiopia, Ireland. | 3 measured depression in pregnancy, 7 measured post-natal depression at ranging times e.g. 6 mths/12mths; 1 no report of measurement of depression. | DSSI, BDI, CESD, EPDS | 20% total prevalence of perinatal depression are unintended pregnancy. 2 higher perinatal depression when pregnancy is unintended. |
| 14 | Aghajafari | 2018 | Vitamin D Deﬁciency and Antenatal and Postpartum Depression: A Systematic Review. | To systematically summarize the association of 25(OH)D and perinatal depression. | 14 | 8 prospective designs as follows: 2 secondary analyses of randomized controlled trials, 4 cross-sectional, 1 nested case-control, 1 randomized controlled trial. | 11,888 women. | Gestational age: 9 -36 weeks, postnatal sampling between birth & 12 months. | Australia, China, Denmark, Iran, Japan, Netherlands, Turkey, & United States. | 7 postnatal depression 5 antenatal depression, 2 measured ante & postnatal depression. | EPDS, CES-D, BDI, MINI, DASS-21, PHQ-9, Physician diagnosis/self-report | Antenatal Depression:(AD) 5 of 7 studies showed signiﬁcant association with lower levels of vitamin D and AD, only 1 did not. 1 found higher levels of vitamin D concentration associated with a higher risk of AD at 16 weeks. Higher vitamin D was associated with reduced AD at 18.5. 21 & 38– 40 weeks gestation. Postpartum Depression (PPD) 5 of 9 studies showed signiﬁcant association between vitamin D in pregnancy and PPD, but 4 showed no association. Low level vitamin D concentration (<47 nmol/L) was associated with increased PPD at 3 days postpartum. Higher vitamin D was associated with decreased PPD at 1 week, 4 weeks, 6 weeks, 8 weeks, 3 months & 7 months. Only 1 study associated low level Vitamin D with higher scores of depressive symptoms, but not with diagnosis of major depression disorder. |
| 15 | Alipour | 2018 | The most important risk factors affecting mental health during pregnancy. | To determine the risk factors of mental health disorders during pregnancy among Iranian women. | 30 | 16 cross-sectional studies + descriptive analytical, case control, cohort, longitudinal & correlational studies. | Sample size was 10,465, with a mean of 267 (standard deviation 304.8). | Iranian with mean maternal age 24-32 years. | Iranian | During pregnancy | GHQ-28, BDI, EPDS, SC90, DASS-21, SSTAI, PRAQ, PSRS | Significant relationship between antenatal mental health risks e.g. depression & anxiety &: lack of social support, marital status, domestic violence, unintended/unwanted pregnancy, & socioeconomic status. Iranian women: socioeconomic status & marital quality posed the highest antenatal health risks. |
| 16 | Alvarez-Segura | 2014 | Are women with a history of abuse more vulnerable to perinatal depressive symptoms? | To 1. Examine the association between maternal lifetime abuse and perinatal depression & 2. Investigate whether there is a difference between the effects of childhood abuse compared to adulthood abuse on perinatal depression. | 43 | 29 cross-sectional & 14 longitudinal. | Sample sizes ranged from 38 to 6,421. | Mostly Caucasian. | USA, Canada, Asia, Oceania, Europe, and Latin America. | Antenatal or postnatal. | EPDS, BDI, CES-D, PHQ-9, HADS, HADS + EPDS + formal diagnostic assessment as per the ICD-10 PCV criteria. | Higher perinatal depressive symptom scores were found in women with any history of lifetime abuse (childhood/adult abuse, or Intimate partner violence (IPV) such as psychological, sexual, or physical aggression). IPV appeared to predict perinatal depression especially when found during the antenatal period. |
| 17 | Azami | 2019 | The association between gestational diabetes and postpartum depression: A systematic review and meta-analysis. | To examine the relationship between Gestational diabetes (GDM) and postnatal depression. | 18 | 15 cohort studies, 2 cross-sectional studies 1 case-control study | Total sample size 2,370,958 | Compare the depression rates women with without GDM. | USA, Europe, Asia. | 7 depression measured pre-10th postnatal week 4 post-10th week 4 measurement time unclear. | EPDS, CES-D, PHQ-9, PHQ-2, CES-D, ICD-10, ICD-9, HADS | Meta-analysis: GDM significantly increased the risk of postnatal depression, relative risk (RR) was 1.59 (95% CI: 1.22–2.07, p = 0.001). 15 cohort studies: RR 1.67 (95% CI: 1.22–2.28), 2 cross-sectional studies RR 1.37 (95% CI: 0.91–2.05) 1 case-control study 1.29 (95% CI: 0.98–1.68). |
| 18 | Barakat | 2014 | What do we know about gestational diabetes mellitus and risk for postpartum depression among ethnically diverse low-income women in the USA? | To examine the relationship between (GDM) and postnatal depression among low-income, ethnic minority women. | 3 | Cohort studies. | Total sample size 16201. | Ethnically diverse low-income women, non-white, with Latinas, African-Americans, and Asians. | USA. | Depression measured during pregnancy & 12 months postnatal. | ICD-9, SF-36, CESD | 1 study by Kozhimannil et al. (2009) suggested a strong relationship between GDM & postnatal depression among ethnically diverse low-income women. The others suggested high rates of postnatal depression among all groups of women in the studies. |
| 19 | Beck | 2001 | Predictors of Postpartum Depression. | To update the predictors of postnatal depression. | 84 | 67 longitudinal 17 were cross-sectional. | Not reported. | Not reported. | USA, Canada, UK, New Zealand, Australia, South Africa, Ireland, France, Japan, Belgium, Portugal, United Arab Emirates, Israel, Switzerland, Brazil, China, Nigeria, Netherlands, Finland. | Depression measured 2 weeks postnatal. | Formal diagnostic assessment EPDS, CESD, BDI, ZSDS, HRSD | Meta-analysis: 13 predictors of postnatal depression.10 moderate predictive relationship; included prenatal depression,self esteem factors, childcare stress, prenatal anxiety, life stress, social support issues, marital relationship issues, previous depression history, infant temperament, maternity blues,3/13 predictors with a small predictive relationship = marital status, socioeconomic status, unplanned/unwanted pregnancy. |
| 20 | Beck | 1996 | A Meta-Analysis of Predictors of Postpartum Depression. | To examine the magnitude of the relationship between postnatal depression and predictors: prenatal depression, past history of depression, social support, life stress, child care stress, maternity blues, marital satisfaction, & prenatal anxiety. | 44 | 40 longitudinal 4 studies cross-sectional. | Not reported. | 38 Convenience sampling 4 random sampling. 2 used matching. | USA, Great Britain, Canada, Scotland, Australia, Ireland, South Africa. | Not reported | PEDQ, EPDS. | Effect sizes were calculated for all predictors via 3 methods: unweighted, weighted by sample size, & weighted by quality index score. R effect Cohen (1988) includes: small (r = .10), medium (r =.30), & large (r = .50). Findings: moderate to large significant effect sizes between postnatal depression & 8 predictors: prenatal depression (49 to .51), child care stress (48 to .49), life stress (.36 to.40), social support (.37 to .39), prenatal anxiety (.30 to .36), maternity blues (.35 to .37), and marital satisfaction (.29 to .37), past history of depression (.27 to .29). |
| 21 | Bell | 2016 | The birth experience and women's postnatal depression: A systematic review. | To systematically identify the relationship between the birth experience and postnatal depression. | 15 | 5 secondary analysis of previously collected data, 5 retrospective designs, 5 prospective designs. | Sample sizes ranged from 44 to 5332 women. | 13 women of all childbearing ages 2 only teenagers. Most studies included both primiparous and multiparous women. | Germany, Israel, Italy, New Zealand, Switzerland, UK, USA and Jordan. | Birth experience and postnatal depression was measured at various points ranging from 2-3 days to 12 months postnatal. | DASS 21, EPDS, CESD, BDI, Some constructed questions by the investigators. | 11/15 found a significant association between birth experience and postnatal depression. Only 4 studies found no significance. |
| 22 | Beydoun | 2012 | Intimate partner violence against adult women and its association with major depressive disorder, depressive symptoms and postpartum depression: a systematic review and meta-analysis. | To identify the magnitude of the relationship between intimate partner violence (IPV ) and depressive outcomes in women (elevated depressive symptoms (EDS) , diagnosed major depressive disorder (MDD) and postpartum depression (PPD)). | 37 | 32 cross-sectional 5 cohort studies. | Sample size ranged from 101 to 7154. | Adult women (pregnant & non-pregnant). Age range: 27 18+ years 10 less than 18 years old. | Jordan, USA, Canada, Ethiopia, New Zealand, Peru, Australia, Brazil, Italy, Hong Kong, Vietnam, South Africa, & Taiwan. (24/37) conducted in USA. | Not reported. | PHQ-9, BDI, CES-D , GHQ, EPDS, ICD-9 or 10, DSM-IV CIDI | 25 EDS, 7 PPD 5 MDD. Majority findings: moderate/strong positive associations between IPV and depression. 2- 3 fold increased risk of MDD 1.5–2-fold increased risk of both EDS and PPD among women who had experienced IPV c.f. those who had not. |
| 23 | Bhati | 2015 | A Systematic Review of the Relationship Between Postpartum Sleep Disturbance and Postpartum Depression. | To identify the association between postpartum sleep disturbance and postpartum depression (PPD). | 13 | 11 prospective longitudinal observational 1 case control 1 survey. | Total 3,793 postpartum women; sample size ranging from 22 to 2,870. | Aged 18-47 years, women were recruited from hospitals, child birth classes, obstetricians’ offices, and women's clinics. Women were in 3rd trimester of pregnancy, or 1day to 8 months postnatal. 90% were white, married or partnered, middle class, and/or socially advantaged. They were primiparous & multiparous. | USA, Australia, Norway & Taiwan. | Depression was measured in 3rd trimester of pregnancy, or 1 day to 8 months postnatal. The timing and measurement of sleep measures varied from 48 hours to 7 days and 4 weeks after postnatal period. | 11 Structured Clinical Interviews 2 Self Report. | Effect size was used as an indicator for the strength of a relationship between PPD and postpartum sleep disturbance categorized as small (.2–.4), medium (.5–.7), large (.8–1), & very large (1.1–2) relationship. Findings: there was significant relationship between sleep disturbance and PPD.1 small effect size (0.4) 12 ranged from moderate to large and very large (0.6–1.7), indicating a strong association between sleep disturbance & PPD. |
| 24 | Biaggi | 2016 | Identifying the women at risk of antenatal anxiety and depression: A systematic review. | To identify the common risk factors of antenatal anxiety and depression. | 97 | Not reported. | Ranged from 39 to 877, 579. | Not reported. | Jordan, Nigeria, Italy, India, Turkey, Pakistan, USA, Canada, Germany, Australia, Hungary, South Africa, Lithuania, Ethiopia, Serbia, Brazil, Portugal, Vietnam, Iceland, Tanzania, Malaysia, Bangladesh, Finland, UK, Switzerland, Sweden, Malawi. | Not reported. | Not reported. | Main risk factors associated with antenatal depression: lacking social or partner support; history of emotional, physical or sexual abuse or domestic violence; personal history of mental illness (eg, depression & anxiety; unplanned /or unwanted pregnancy; experience of stressful life events high perceived stress; present/ past pregnancy/delivery complications;& pregnancy loss. |
| 25 | Brown | 2018 | Chronic medical conditions and peripartum mental illness: A Systematic Review and Meta-Analysis. | To examine the association between maternal chronic medical conditions (CMCs) and peripartum mental illness (such as depression and anxiety). | 12 | 9 prospective/retrospective cohort studies 7 cross-sectional. | Sample size ranged from 305 to 707,701 women. | Studies measured multiple CMCs such as diabetes; thyroid disease; hypertension or heart disease; asthma; multiple sclerosis , epilepsy, migraine, or neurological conditions in general; & gastrointestinal disorders. 12 measured antepartum, postpartum, or peripartum depression. 1 did not distinguish between peripartum depression & anxiety; 2 measured peripartum depression & anxiety, 1 postpartum depression, anxiety & comorbidity. | USA, Canada, UK, Finland, Sweden, Norway, Peru, Lebanon, Qatar. | Not reported. | EPDS, HSC, PHQ | 14 focused on depression & 2 focused on anxiety. MCs overall were associated with peripartum mental illness (adjusted pooled odds ratios (aPOR) = 1.43, 95% confidence interval (CI): 1.25, 1.63). CMCs overall were associated with antepartum (aPOR = 1.41, 95% CI: 1.10, 1.81) & postpartum mental illness Independently (aPOR = 1.44, 95% CI: 1.13, 1.85), & with peripartum depression (aPOR = 1.45, 95% CI: 1.25, 1.67) & anxiety Independently (aPOR = 1.63, 95% CI: 1.35, 1.95). Certain illnesses e.g. diabetes (aPOR = 1.34, 95% CI: 1.07, 1.69), hypertension/heart disease (aPOR = 1.60, 95% CI: 1.05, 2.45), migraine (aPOR = 1.75, 95% CI: 1.20, 2.54), & other neurological disorders (aPOR = 1.45, 95% CI: 1.19, 1.77), but not asthma, were each associated and at increased risk of peripartum mental illness such as peripartum depression. |
| 26 | Campbell-Jackson | 2014 | The psychological impact of stillbirth on mothers. A Systematic Review. | To examine the psychological symptoms of stillbirth on mothers. | 26 | 8 qualitative (Interpretative ethnographic; questionnaire survey & In-depth interviews).   18 quantitative studies longitudinal case control cohort studies survey questionnaire. | Quantitative samples ranged from 17 to 2,292. Qualitative samples ranged from 5 to 47. | Women who experienced stillbirth (from 20 weeks gestation). Majority: white European, with college education or higher, married, & higher socioeconomic status. Quantitative generally recruited women from hospitals or clinics. | Australia, UK, USA, Canada, Netherlands, Taiwan, Sweden, & Japan. | Not reported. | 7 SDAS items, ZSRDS, CES-D, EPDS GHQ | Stillbirth is reflected as a difficult experience, increasing psychological symptoms like anxiety & depression, especially in the early months post stillbirth. But it can still be in evidence up to 3 years later. Additional risk factors for higher levels of depressive symptoms are: higher parity at the time of stillbirth being unmarried. Lack of social & emotional support during the loss may be a predictor for later anxiety and depression: women may lack a partner or someone to talk to / express their feelings to about their distressing experience. |
| 27 | Carter | 2006 | Depression, Caesarean Section and Postpartum Depression: A Review of the Evidence Examining the Link. | To examine the association between caesarean section (CS) and postnatal depression. | 24 | RCT, case-control studies, cohort studies, case-series ''women selected who were either pregnant or had recently given birth, not on the basis of either exposure to the risk factor or the presence of the outcome variable''. | Sample size ranged from 34 to 10934. | Women with experience of CS. | Australia, UK, USA, Canada, Taiwan, Sweden, France, Lebanon, Denmark, Finland. | Ranged from 10 days to 1 year postnatal. | EPDS, BDI, CEDS, BPDS | 15 /24 found no significant association between postnatal depression and CS 4 showed mixed findings. 5 found a significant association. Methodologically superior studies and meta-analyses did not find adverse association between CS & postnatal depression. CS may operate as a risk factor if women are vulnerable or at risk, but it is not a powerful predictor of postnatal depression. |
| 28 | Choi | 2016 | Childhood maltreatment and perinatal mood and anxiety disorders: A Systematic Review. | To examine the association between maternal histories of childhood maltreatment and perinatal mood and anxiety disorders (PMAD). | 35 | 60% longitudinal 23% cross-sectional 10% intervention studies 7% retrospective. | 26,239 participants Sample sizes ranged from 44 to 5,380. | The focus was on pregnant adolescents. Many studies included females under 18 years old. | USA, Israel, Australia, France, Vietnam, Netherlands, Canada, Spain. | Pregnancy to postnatal. | EPDS, BDI, PDSS, BSSQ, CIS, CDRS | Most studies focused on perinatal depression and histories of childhood maltreatment (e.g. physical & sexual abuse, emotional abuse and/or neglect). 14/18 found significant positive associations between childhood maltreatment & antenatal depression, mostly in 2nd & 3rd trimesters of pregnancy. 13/16 studies also found significant associations between childhood maltreatment and postnatal depression. |
| 29 | Dama | 2016 | Thyroid peroxidase autoantibodies and perinatal depression risk: a systematic review. | To examine the association between abnormal levels of autoantibodies thyroperoxidase (TPO-AB) and perinatal depression. | 11 | 1 case-control design 2 cross-sectional design 8 prospective cohort designs. | Sample sizes ranged from 74 to 1191. | Not reported. | Not reported. | Ranged: 1st pregnancy trimester to 1 year postnatal. | Clinical syndrome: SDIs, RDC, CIDI, DIG, SCID-NP for DSM-III-R, Clinician-rated questionnaire scales: HAM-D MADRS HADS EPDS, GHQ-30 POMS-D Investigator developed questionnaire | Antenatal depression 5/11 studies examined associations between TPO-AB during pregnancy at 12–25 weeks and antenatal depression. 3 showed significant association but 2 did not. Postnatal depression 5/11 studies examined associations between TPO-AB during pregnancy at 12–25 weeks and postnatal depression. 4 found significant associations and 1 did not. Of 4 studies that examined the associations between TPO-AB during the postnatal period and postnatal depression, 2 found significant associations but 2 did not. The review suggests that ‘elevated’ (TPO-AB+) in early to mid-pregnancy is related to antenatal depression and may be a risk factor for postnatal depression. |
| 30 | Delahaije | 2013 | Anxiety and depression following preeclampsia or haemolysis, elevated liver enzymes, and low platelets syndrome. A systematic review. | To examine the association between women with preeclampsia (PE) or HELLP (haemolysis, elevated liver enzymes, and low platelets) syndrome and depression or anxiety, c.f. women without a history of PE/HELLP. | 6 | 4 retrospective cohort studies 2 prospective cohort studies. | Total participants 5636 Sample sizes ranged from 20 to 4941. | 2 women with severe PE & women with uncomplicated normotensive pregnancies 1 included women with severe, early‐onset PE/HELLP with preterm delivery & healthy participants with a preterm delivery. 1 3 groups, women with PE, women with preterm premature rupture of membranes & women with uncomplicated pregnancies. 2 4 groups, women with preterm PE, women with term PE, women with preterm birth & women with an uneventful pregnancy. | Netherlands. | Within pregnancy. | BDI, BDII, EPDS, CES-D, PSS‐SR, PTSD-SS-SRQ, SSSRQ | 6 found positive associations between PE/HELLP and prevalence of depression/severity of depressive symptoms. There was an increased prevalence of 26% among women with PE and term births versus 7% of healthy term women. it also found increased severity with 2.7 among women with PE and preterm deliveries versus 1.2 of healthy preterm mothers. |
| 31 | Dias | 2015 | Breastfeeding and depression: a systematic review of the literature. | To examine the relationship bewteen breastfeeding and depression during perinatal period. | 48 | Not reported. | Total: 71,245 participants. | Pregnant & postnatal women | USA, UK, Australia, Brazil, Canada, Barbados, Norway, Pakistan, Turkey, China, Congo, Finland, Iceland, Italy, Japan, Mexico, Portugal, Sweden, United Arab Emirates. | Pregnancy across trimesters to 1 year postnatal. | EPDS, CES-D, BDI, a clinical interview. | Breastfeeding duration is associated with postnatal depression among all studies published in the last 30 years. Postnatal depression predicts early breastfeeding cessation. Negative breastfeeding experiences (e.g. breastfeeding worries /difficulties, negative attitudes, pain, & a low self-efficacy) may lead to the development of depressive symptoms. Gestation and postnatal depression are both linked to a shorter breastfeeding duration. Antenatal depression may predict shorter breastfeeding duration and that may increase the risk of postnatal depression. |
| 32 | Elwood | 2019 | A systematic review investigating if genetic or epigenetic markers are associated with postnatal depression. | To examine the association between genetic or epigenetic factors and postnatal depression. | 37 | Not reported. | Ranged from 48 to 10,759. | Not reported. | USA, Canada, UK, Brazil, China etc. | Pregnancy across trimesters to 1 year postnatal. | Structured clinical interview/clinical diagnosis, STAI, HDRS, BDI | Positive associations between postnatal depression & polymorphisms in the hemicentin-1(HMNC1), the catechol-O-methyl transferase (COMT), the mono-amine-oxidase type ( MAOT),the protein kinase C beta gene (PRKCB), the oestrogen receptor gene (ESR1), S allele (SLC6A4) genes in the presence of adverse life events. Additional findings: Positive associations between postnatal depression & polymorphisms in the Brain Derived Neurotrophic Factor (BDNF) gene when the postnatal period takes place during fall and winter seasons; & the Oxytocin (OXT ) and oxytocin receptor (OXTR ) genes when the mother experienced childhood adversity. Risk factors for postnatal depression: Epigenetic in relation to genotype, oestrogen, and mothers who experienced childhood adversity. |
| 33 | Howard | 2013 | Domestic violence and perinatal mental disorders: a systematic review and meta-analysis. | To identify the prevalence / or odds of having experienced domestic violence among women with perinatal mental disorders (such as depression and anxiety disorders). | 67 | Longitudinal studies cross-sectional studies. | Not reported. | 16 or more years assessed as having a perinatal mental disorder. | USA, Africa, Europe, Australia, Middle East & Asia. | Pregnancy & postpartum. | Clinical diagnosis, Self-report, EPDS, BDI. | Cross-sectional data on pooled prevalence found that women with probable antenatal and postnatal depression had a 3-to 5-fold increased risk of having experienced domestic violence during their lifetime, the past year, and during pregnancy. Meta-analysis of longitudinal data found that women who had experienced domestic violence during pregnancy had a 3-fold increased risk of developing postnatal depression (OR 3.1, 95% CI 2.7–3.6). |
| 34 | Hutchens | 2017 | Survivors of child maltreatment and postpartum depression: an integrative review. | To identify the relationship between a mother with history of child maltreatment and postnatal depression. | 16 | 6 cross‐sectional 10 longitudinal. | Sample size ranged from 53 participants to 5380. | Samples were varied in age, ethnicity, & nationality, for example the racial diversity of white, African American and Hispanic women. There were adults and adolescents in the studies. | Canada, Australia, US, Spain, the Netherlands. | Longitudinal studies: early measurement of depression: 12th week of pregnancy to 1 year postpartum. Cross‐sectional studies: ranged from early measurement from 1-2 days postpartum to 8 -10 weeks postpartum. | Clinical diagnosis, EPDS, BDI, HDRS, HADS, PDSS, CES-D | Despite the variety in quality, design, sampling, population characteristics, time frame, different instruments, and operational definitions of child maltreatment (e.g. physical, sexual abuse , neglect & emotional abuse) 12/16 studies found a significant relationship between child maltreatment and postnatal depression. The sensitization model of stress may explain the association of child maltreatment with postnatal depression. It claimed that women who suffered early maltreatment are sensitized to react in repetitive, depressive, or maladaptive behaviours during future stress. Another established pathway for this association relates to the early development of insecure or traumatic attachments that lead to low self-esteem, a known risk factor for depression. |
| 35 | Hymas | 2018 | Predicting postpartum depression among adolescent mothers: A systematic review of risk. | To examine the risk factors associated with adolescent postnatal depression. | 14 | Most studies were longitudinal 1 retrospective cohort 1 cross-sectional observational. | Sample sizes ranged from 60 to 17,823 adolescents. | Ages ranged from 12 to 19 years.  Ethnicities most represented: Caucasian, African American, European American, Black, Hispanic and Métis. | Developed countries only: USA, Canada, Portugal, Sweden & Australia. | 1 month to 1 year postpartum. | EPDS, CES-D, BDI, GHQ. | Significant Risk factors for adolescent postnatal depression Socio-economic variables e.g. economic hardship employment and education levels past history of depression lack of social support Younger maternal age was identified as significant in only 2 studies. |
| 36 | Lambert | 2019 | Inflammatory Biomarkers and Postpartum Depression: A Systematic Review of Literature. | To examine inflammatory biomarkers associated with postnatal depression. | 52 |  |  |  |  |  | BDI, EPDS, KGB, HAMD, MARDS, MINI, SIGH-ADS 29, ZDRS | Possible predictor of postnatal depression? The dosage of some inflammation biomarkers, in particular C-reactive protein (CRP) in the 3rd trimester of pregnancy or immediately postnatal may predict postnatal depression. Interactions between inflammation & the dysfunction of corticotropic axis could illuminate PPD onset of postnatal depression. |
| 37 | Lara-Cinisomo | 2018 | A systematic review of cultural orientation and perinatal depression in Latina women: are acculturation, Marianismo, and religiosity risks or protective factors? | To examine whether acculturation, Marianismo, and religiosity are risks or not factors for postnatal depression in Latina women who are living in the USA, Latin America, and other countries. | 10 | 4 at least 1 interview prenatal to postpartum 6 cross-sectional studies. | Sample size ranged from 60 to 3952. | Immigrant or native-born Latina women aged 18 years and older. | USA and Mexico. | Not reported. | EPDS, BDI, CES-D, PHQ-9, SCID/DSM-IV | Degree of acculturation was contradictorily related to postnatal depression. Risk of antenatal depression was inconsistently related to 'Marianismo', (the traditional female role of virtue, passivity, & primacy of others over oneself), but: significantly and indirectly related to risk of postnatal depression. Religiosity appeared to act as a protective factor against postnatal depression. |
| 38 | Moameri | 2019 | Association of postpartum depression and cesarean section: A systematic review and meta-analysis. | To examine the association between cesarean section (CS) and postnatal depression. | 32 | 32 cohort /case control studies. | Sample size ranged from 106 to 70, 7701 women. | Mean age ranged from 25 to 33 years. | Not reported. | Not reported. | EPDS, BDI, PHQ-9, BPDS-ICD | Emergency CS are at higher risk of postnatal depression than elective CS The adjusted OR of the association between CS & postnatal depression was 1.15 (95% CI: 1.00, 1.34). The OR of the association of elective CS and postnatal depression was 1.29 (1.12, 1.49) For emergency CS and postnatal depression was 1.36 (1.20, 1.55).The pooled relative risk of the association between CS and postnatal depression was 1.22 (0.94 1.58). |
| 39 | Molyneaux | 2014 | Obesity and mental disorders during pregnancy and postpartum: a systematic review and meta-analysis. | To examine the prevalence and risk of antenatal and postnatal mental disorders among obese and overweight women. | 62 | Cohort case control cross-sectional studies. (39 antenatal depression 23 postnatal depression). | Total: 540,373 women. | Obese or overweight or  normal weight women. | Most High-Income Countries 9 from Low or Middle-Income Countries. | Pregnancy: 10 to 36 weeks Postpartum: 1 week to 1 year. | EPDS, CSED. | Weight: Obese and overweight women had significantly increased risks of having elevated depressive symptoms during both the antenatal and postnatal periods than normal-weight women: Antenatal (obesity OR 1.43, 95%CI 1.27-1.61, overweight OR 1.19, 95%CI 1.09-1.31; average prevalence: 33.0% obese, 28.6% overweight, and 22.2% normal weight) Postnatal (obesity OR 1.30, 95%CI 1.20-1.42, overweight OR 1.09, 95%CI 1.05-1.13; average prevalence: 13.0% obesity, 11.8% overweight, 9.9% normal weight). |
| 40 | Moura, D | 2016 | Oxytocin and depression in the perinatal period—a systematic review. | To examine the relationship between Oxytocin (OT) as a potential depressive biomarker and perinatal depression. | 6 | Not reported. | Total: 620 pregnant women. | Women aged 18 and over. | Not reported. | During pregnancy & postnatal. | EPDS, BDI, CSED, SSI | 4 out of 6 studies associated low OT levels during both ante and postnatal periods with the development of depressive symptoms. This association does not have statistical significance among 2 of the 6 studies. |
| 41 | Olieman | 2017 | The effect of an elective caesarean section on maternal request on peripartum anxiety and depression in women with childbirth fear: a systematic review. | To examine the effect of elective caesarean section (ECS ) on request on perinatal anxiety and depression. | 3 | 3 cohort studies. | Sample size ranged from 91 to 50,462. | Not reported. | Not reported. | Ranged from 30 weeks antenatal to 6 months postnatal. | EPDS, SCL-Anxiety (Hopkins) & SCL-8 (Hopkins). | Elective ECS Women who requested an ECS had higher level of antenatal depression and anxiety, but no observed difference in levels of postnatal depression than those with vaginal deliveries. 1 good quality study found that women who preferred ECS but delivered vaginally had significantly higher levels of childbirth fear, higher symptom levels of post-traumatic stress disorder (PTSD) & depression than women who preferred & delivered vaginally. |
| 42 | Orta | 2018 | The association between maternal cortisol and depression during pregnancy, a systematic review. | To examine the associations between maternal cortisol levels and antenatal depression through timing of cortisol collection. | 29 | Not reported. | Sample size ranged from 29 to 2810. | Mean age range 17 to 37 yrs. Cortisol measured daily via morning collections of saliva, blood, & urine. | European / North American countries included: USA, UK, Canada, Sweden, Norway, and Germany. | Pregnancy: different trimesters. | EPDS, BDI, CES-D, PHQ, SCID | 17 out 29 no association between maternal cortisol & antenatal depression. In 12 studies, an association was found between elevated 2nd & 3rd trimester cortisol levels & depressed women, not present in non-depressed women. The most commonly reported matrix used for cortisol collection were saliva and blood usually collected in the 2nd & 3rd trimester mornings of gestation. |
| 43 | Razurel | 2013 | Relation between perceived stress, social support, coping strategies and maternal well-being. | To examine the association between perceived stress, social support, coping mechanisms and maternal well-being in terms of depression and anxiety. | 37 | 33 longitudinal 4 cross-sectional studies. | Sample size ranged from 42 to 2,365. | Not reported. | Not reported. | Pregnancy to postnatal. | EPDS, BDI, CES-D, HADS, STAI. | The level of perceived antenatal stress was associated with developing antenatal & postnatal depression. Similarly, perceived postnatal stress was associated with postnatal depression & anxiety. The types or nature of stress played a role in perinatal depression. E.g. stress because of childcare was not associated to postnatal depression But stress caused by the parental role created a risk of developing postnatal depression. Lack of social support & family conﬂicts was also associated to both perinatal depression & anxiety. |
| 44 | Ross | 2009 | The prevalence of postpartum depression among women with substance use, an abuse history, or chronic illness: a systematic review. | To examine the prevalence of postnatal depression among women with substance use, an abuse history, or chronic Illness. | 17 | Not reported. | Not reported. | Substance abusers, abuse history or chronic illness. | Finland, USA, China & Canada, Lebanon & Switzerland. | Ranged from 0–52 weeks postpartum. | EPDS, CES-D, BDI, DSM-IV | Substance users & women with a history of abuse showed consistently high rates of postnatal depression. In contrast, there was little evidence associating women with chronic illness to the risk of postnatal depression. |
| 45 | Serati | 2016 | Perinatal Major Depression Biomarkers: A systematic review. | To examine the biological biomarkers that might be used for early detection of perinatal depression. | 127 | Various including; case-control designs, cross-sectional design & cohort studies. | Sample size ranged from 19 to 1745 pregnant women. | Not reported. | Not reported. | Ranged from antenatal to postnatal. | Not reported. | Biochemical studies had not associated maternal iron status with postnatal depression, whereas biopterin, homocysteine, zinc, & vitamin B12 levels associated significantly with postnatal depression. Low vitamin D levels associated to higher depressive symptoms during the perinatal period. In immunological studies, prolonged or excessive proinflammatory immune system activation (IL-1, IL-6, and TNF-α) acts as a predictor for developing postnatal depression. Most studies found an association between cytokines and postnatal depression, despite a little evidence finding no association. In Endocrinological studies, alteration of the HPA axis is identified to be a robust biomarker for depression. Mid-pregnancy depression has been significantly associated with increased cortisol level and OXT concentration. |
| 46 | Shamblaw | 2019 | Abuse as a risk factor for prenatal depressive symptoms: a meta-analysis. | To examine the association between history of abuse and antenatal depression. | 70 | Not reported. | Total: 62,438 participants. | Pregnant women with a lifetime history of abuse. | Developed and developing countries. | During pregnancy. | SCID, HASD, PHQ, CESD, EPDS, BDI, SCL-90, IACLIDE, IDS, CIS. | A lifetime history of abuse is as strong predictor for antenatal depression as well as for depression in the general population. An abuse history was associated significantly with antenatal depression, with effect sizes in the moderate range for: any abuse: r¯ = 0.287 (95% CI = 0.220, 0.351), p < 0.001; physical abuse : r¯ = 0.271 (95% CI = 0.243, 0.299), p < 0.001; sexual abuse: r¯ = 0.259 (95% CI = 0.220, 0.296), p < 0.001; emotional abuse: r¯ = 0.340 (95% CI = 0.229, 0.442), p < 0.001. |
| 47 | Silveira | 2015 | The role of body image in prenatal and postpartum depression: a critical review of the literature. | To examine the association between body image and perinatal depression. | 19 | Cross-sectional & cohort studies. | Sample size ranged from 30 to 506. | Majority: homogenous samples of white women or college-educated or with middle-to-high income levels. A few studies included more than15 % Hispanic or African-American women. | USA, Australia, a few from other international countries | Ranged from pregnancy to 1 year postpartum. | CES-D, BDI, PHQ, EPDS. | Body image dissatisfaction (BID) 'consistently but weakly' associated with antenatal & postnatal depression by most studies. Cross-sectional: BID & perinatal depression consistently associated   Cohort studies: BID linked to onset of perinatal depression 'steadily but weakly'. Prospective studies: depression appeared to influence the onset of some aspects of antenatal BID but found little BID in the postnatal period. |
| 48 | Sparling | 2017 | The role of diet and nutritional supplementation in perinatal depression: a systematic review. | To examine whether dietary intake influences the risk for antenatal and postnatal depression. | 35 | 6 randomized controlled trials 12 cohort 1 case‐control 16 cross‐sectional studies. | Total: 88,051 | Recruited from hospitals or clinics. | Global. | Ranged from pregnancy to 1 year postpartum. | EPDS, CES-D, BDI, SCID‐CV, K‐10 PDSS. | Prevalence of perinatal depression: 10% to 15%. Cross‐sectional: many showed a very high prevalence of depression from 40% to 94%. Cohort studies found prevalence of perinatal depression from 12% to 31.7%.  4 main study categories: dietary intake full panel of essential nutrients 8 specific nutrients (including B vitamins, Vitamin D, calcium and zinc) 12 intake of fish or polyunsaturated fatty acids (PUFAs). Limited evidence found that dietary intake influences the risk of developing perinatal depression. 13 (including three PUFA supplementation trials), showed no evidence of relationship 22 found some protective effects from using healthy dietary patterns, multivitamin supplementation, fish & PUFA intake, calcium, zinc and selenium. |
| 49 | Sparling | 2017 | Nutrients and perinatal depression: A systematic review. | To examine whether blood nutrient levels influence the risk of perinatal depression. | 24 | cohort, case‐control, cross‐sectional studies. | Total: 14262. | Not reported. | Global. | Ranged from pregnancy to 1 year postpartum. | DSM-IV, SCID-CV, EPDS, CES-D. | Overall, evidence on inﬂuence of nutritional biomarker levels on perinatal depression is inconsistent due to the methodological limitations, with stronger evidence for some nutrients. 14 found associations between perinatal depression and lower levels of folate, vitamin D, Fe, Se, Zn, & fats & fatty acids.2 found associations between perinatal depression and higher nutrient levels 8 found no evidence of a relationship. |
| 50 | Steinig | 2017 | Antenatal and postnatal depression in women with obesity: a systematic review. | To examine the association between pre-pregnancy obesity and perinatal depression. | 14 | 8 prospective 6 cross-sectional. | Sample size ranged from 219 to 13,314. | Pre-pregnancy obese & normal weight women. | USA, UK, Belgium, Germany, Finland, Iran, Serbia, Australia, Ireland, New Zealand. | Ranged from pregnancy to 1 year postpartum. | CES-D, BDI, HADS, DSM-IV, SCID, EPDS. | 9 13 studies investigating pre-pregnancy obesity & depression found that obese women are at a higher risk of developing depression during pregnancy than women of normal weight. 4 showed no relationship. 2 4 studies investigating pre-pregnancy obesity and depression after the birth found association.1 found diverse results. 1 found no association. |
| 51 | Suzuki | 2019 | Association of second- hand smoke and depressive symptoms in non-smoking pregnant women: A systematic review and meta-analysis. | To examine the association of antenatal depression and exposure to second hand smoke (SHS) in non-smoking pregnant women. | 7 | 7 prospective cohort studies. | Not reported. | Non-smoking pregnant women exposed to SHS. | USA, China, Japan. | Not reported. | Not reported | Most studies found an association between SHS exposure & increased risk to the mental health of ante & postnatal women. 2 involving a total of 4103 women found that antenatal and postnatal depression had significantly increased (ORs = 1.77 [95% CI = 1.12 – 2.79]; p = 0.01; I2 = 28%), & significantly increased antenatal suicidal ideation in 2670 SHS exposed women (ORs = 1.75 [95% CI = 1.14 – 2.70]; p = 0.01; I2 = 51%). |
| 52 | Teychenne | 2013 | Physical activity, sedentary behaviour, and postnatal depressive symptoms: a review. | To examine the association between physical activity, sedentary lifestyle, and postnatal depression. | 17 | 10 observational (cross-sectional, longitudinal) 7 intervention studies (RCT & nonrandomized trials) . | Sample size ranged from 18 to 4720. | Non–clinically depressed postpartum women. | Most from USA & Australia. | Ranged from pregnancy to 1 year postpartum. | EPDS, BDI, CES-D. | 16 showed an inverse association between physical activity (either pre-gestation, during gestation or postnatal) & postnatal depressive symptoms. 1 positively related occupational physical activity to postnatal depression. The evidence was inconclusive regarding protection against postnatal depression by an 'ideal dose' of postnatal physical activity and there was very little evidence to link sedentary behaviour to postnatal depression. |
| 53 | Ti | 2019 | postpartum hormonal contraception use and incidence of postpartum depression: a systematic review. | to examine the association between postnatal hormonal contraceptive use and postnatal depression. | 4 | 2 RCTs 2 retrospective cohort studies. | 76,409 participants. Sample size ranged from 181 to 75,528. | Women using injectable forms of contraception recruited from teaching hospitals/outpatient obstetric clinics. Comparison groups were generally women not using contraception or a hormonal method (e.g. barriers or copper IUD). | South Africa or USA. | Ranged from 1 month to 1 year postpartum. | EPDS, BDI-II, MADRS. | No differences in rates of postnatal depression between women using postpartum depot medroxyprogesterone & those not using hormonal contraception. But women receiving injectable norethisterone enanthate immediately after the birth are at a 2–3x increased risk of developing postnatal depressive symptoms at 6 weeks, (not observed at 3 months.) .When combined hormonal contraception, progestin-only pills (POPs), etonogestrel implants, and levonorgestrel intrauterine devices (LNG-IUDs) were compared to no hormonal contraception, research indicates a 35â€“44% lower risk of postpartum depression with POPs and LNG-IUDs, a slightly higher risk of postpartum antidepressant use among women using the etonogestrel implant and vagina. |
| 54 | Wang | 2018 | Association between vitamin D deficiency and antepartum and postpartum depression: a systematic review and meta-analysis of longitudinal studies. | To examine the association between vitamin D deficiency and perinatal depression. | 9 | 7 cohort studies, 1 case–control design, 1 randomized controlled trial. | Total 8470 participants. Sample sizes ranged from 153 to 4236. | Mean age ranged from 26 to 31.2. At baseline, mean gestational age ranged from 5 to 39 weeks. | Australia, USA , Brazil, China, Amsterdam, Turkey, Denmark, Iran. | during pregnancy and postpartum period. | EPDS, CES-D. | Serum 25(OH)D levels < 50 nmol/l was associated with 2.67 times (OR 3.67; 95% CI 1.72–7.85) increased risk of developing postnatal depression c.f. those who had 25 (OH)D levels ≥ 50 nmol/l. No significant association was found between low vitamin D levels and depression during pregnancy with a serum 25(OH)D cut-off level of 30 nmol/l (OR 1.47; 95% CI 0.92–2.36). |
| 55 | Warfa | 2014 | Adult attachment style as a risk factor for maternal postnatal depression: a systematic review. | To examine the associations between attachment style and postnatal depression. | 20 | quasi-experimental  longitudinal cross-sectional studies. | Total: 2306 participants. Sample size ranged from 49 to 319 mothers. |  | Majority from Western countries, but cohorts from Israel & Turkey. | Ranged from 8 weeks to 12 months postpartum. | EPDS, SCID, CIDI-D, CES-D, HADS-D. | Insecure adult attachment style can create an additional risk factor for postnatal depression. Insecure & anxious adult attachment styles were related to postnatal depression more commonly than those that were avoidant or rejecting. Factors associated with adult attachment style include: low economic status, being female, childhood maltreatment, parental psychopathology and neuroticism, lower levels of marital relationship quality /satisfaction social support. |
| 56 | Wassef | 2019 | Anaemia and depletion of iron stores as risk factors for postpartum depression. | To examine the association between anaemia and/or iron-deficiency and postnatal depression. | 17 | 6 randomized-controlled trials 11 observational studies, (2 case-controls, 9 cohort studies). | Sample size ranged from 37 to 729 women. | Compared postnatal depression in women with or without anaemia, low iron stores, or both. | USA, Europe, Asia, Africa, Middle East. | Ranged from 12–20 weeks of pregnancy to 9 months postpartum. | EPDS, CES-D, SCL-90-R. | 8 of 10 studies focused on association of postnatal depression & anaemia & found a higher risk of postnatal depression among anaemic women (r − 0.19 to −0.43 and ORs 1.70–4.64). Postnatal low ferritin level was associated with increased risk of postnatal depression, but without association during pregnancy. 4 studies found that postnatal iron supplementation was associated with decreased postnatal depression. But iron supplementation during pregnancy did not seem to protect against postnatal depression. |
| 57 | Wittkowski | 2014 | Culturally determined risk factors for postnatal depression in Sub-Saharan Africa: a mixed method systematic review. | To identify culturally determined risk factors of postnatal depression within Sub-Saharan Africa. | 12 | 9 quantitative: (7 cross-sectional design, 1 cohort study 1 case-controlled study) 3 qualitative studies. | Quantitative: sample size ranged from 83 to 1035 women. Qualitative studies (2 N=78 & N=55 3rd study not reported. | Women recruited from community or clinical setting. | Sub-Saharan Africa. | Not reported. | DSM-IV, ICD-10, SPI, PDQ, SQR-25, EPDS, SDS, GHQ-28. | Risk factors shared with developed countries included: lack of support, age, depression during pregnancy, unwanted pregnancy, and difficulties in relationships. Specific African cultural factors included: polygamy, rejected paternity, negative cultural perception of others, the threat of spirit attack following African cultural customs. |
| 58 | Wojcicki | 2011 | Maternal omega-3 fatty acid supplementation and risk for perinatal maternal depression. | To identify the association between omega-3 fatty acid supplementation and perinatal depression. | 10 | 3 longitudinal cohort studies, 5 randomized controlled trials 2 pilot trials. | Sample size ranged from 7 to 54,202 women. | Not reported. | Not reported. | Ranged from pregnancy to the 12th postnatal month. | EPDS, BDI, HAM-D, CGI 5 diagnostic interview schedules. | Most studies (6/10) showed no association between Omega-3 polyunsaturated fatty acid (PUFA) supplementation & perinatal depression, but 2 mixed findings. 2 studies found a positive association between Omega-3 PUFAs and decreased risk of perinatal depression: thought more likely to have been: using higher doses, close to 2 g of docosahexaenoic acid (DHA)+  eicosapentaenoic acid (EPA), taking the supplementation earlier in gestation. |
| 59 | Wong | 2009 | The role of traditional confinement practices in determining postpartum depression in women in Chinese cultures: a systematic review of the English language evidence. | To examine relationship between confinement and postpartum depression in Chinese cultures. | 16 | 6 qualitative 10 various quantitative approaches. | Quantitative sample ranged from 50 to 959 women. Most of the Qualitative studies had very small samples (≤ 20 participants). | 2 included primiparae, 4 those that were married (3 women aged ≥ 20 years & 4 women who spoke a particular language. All recruited participants from clinical services, no report of numbers of home births. | Taiwan, Hong Kong, ethnically Chinese groups in Australia, Singapore, Scotland, UK. | Ranged from pregnancy to specific different points of the postnatal month. | SSI (Semi-Structured Interviews), EPDS, GHQ, PSSQ, CES-D, BDI | The role of confinement in postnatal is complicated: 8 found that confinement practice may offer protective factors; 4 found that confinement practices increased risk of postnatal depression. 4 had inconclusive results. Aspects associated to postnatal depression include: low social support in modern society, conflict with mothers-in-law Increased pressure or stress on modern women trying to balance traditional and modern values. |
| 60 | Wosu | 2015 | History of childhood sexual abuse and risk of prenatal and postpartum depression or depressive symptoms: an epidemiologic review. | To examine the association between history of childhood sexual abuse (CSA) and perinatal depression. | 14 | Cross-sectional prospective studies. | Sample size ranged from 40 to 8292. | Ante & postnatal (within months to years) exposed to CSA <18 years of age/adulthood. | USA, Germany, Israel, Canada, Spain, UK. | Ranged from pregnancy to specific different points of the postnatal month. | CES-D, EPDS, BDI-II, SCID. | 6 of 7 studies of antenatal depression consistently found positive & statistically significant associations between women exposed to CSA & antenatal depression or depressive symptoms. However, results on CSA & postnatal depressive symptoms were mixed. The combined unadjusted odds ratio was 1.82 (95% confidence interval (CI) 0.92, 3.60), while the combined adjusted odds ratio was 1.20 (95% CI 0.81, 1.76). |
| 61 | Wu | 2012 | Violence as a risk factor for postpartum depression in mothers: a meta-analysis. | To examine the association between violence and postnatal depression. | 6 | 1 quasi-experimental study 2 prospective cohort studies 3 cross-sectional surveys. | Sample size ranged from 125 to 1,519 with a total of 3,950. | Women aged >18 exposed to violence c.f. women not exposed to violence. | USA, Iran, China, Brazil, India, Pakistan. | Ranged from 2 weeks to 1 year postnatal. | Majority: EPDS, AKUADS, DSS. | 6 studies conclude that violence creates a major risk factor of postnatal depression [OR = 3.47; 95% confidence interval (CI; 2.13–5.64)]. The heterogeneity was highly significant in this meta-analysis (P < 0.00001; I 2 = 79%) Publication bias was identified by a funnel plot. A sensitivity analysis found 3.00, 95%CI (2.44–3.68), p < 0.00001, underlining the stability & reliability of this review. |
| 62 | Xiao | 2014 | The impact of sleep, stress, and depression on postpartum weight retention: a systematic review. | To examine the effect of sleep, stress, and depression on postnatal weight retention. | 13 | 2 cross-sectional studies 11 longitudinal studies. | Sample size ranged from 74 to 37, 127. | Most studies recruited participants from hospitals, community newspaper birth announcements or from registry of gestating women. Mean sample ages ranged from 26 to 38 years. | USA, Netherlands , Belgium , UK, Denmark, Taiwan. | Ranged from 20 weeks antenatal to 3 years postnatal. | EPDS, Chinese version of the Beck Depression Inventory ,CES-D, and Symptoms Checklist-92 . | 3 of 7 studies focusing on associating postnatal depression & postnatal weight retention found non-significant association 6 months after the birth & postnatal weight retention over 5 kg at 1 year postpartum, & over 0.4 kg at 2.6 years postnatal. 4 studies found significant associations, claiming that women who experienced only postnatal depression had a higher risk of postnatal weight retention over 5 kg, c.f. women who had not experienced depression. Findings conflicted regarding women who had experienced both antenatal & postnatal depression. Studies reported that depression in both phases had not increased women's risk of postnatal weight retention over 5 kg at 1 year after the birth. |
| 63 | Xu | 2017 | Caesarean section and risk of postpartum depression: A meta-analysis. | To examine the association between elective caesarean section (ElCS) and emergency caesarean section (EmCS) and postnatal depression. | 28 | cohort cross-sectional case control studies. | Total: 532,630 participants. Sample size ranged from 86 to 472,583. | Women with (ElCS) or (EmCS). | Asia, North America, Europe, South America, Oceania. | Ranged from 2 weeks to 9 months postnatal. | Majority EPDS. A few: ICD-9-CM, ICD-10. | The pooled OR for the relationship between CS and postnatal depression was 1.26 (95% CI: 1.16–1.36). In subgroup analyses grouped by study design, the pooled ORs were: for cohort studies, (1.25, 95% CI: 1.10–1.41); case-control studies, (1.25, 95% CI: 1.00–1.56) cross-sectional studies (1.44, 95% CI: 1.14–1.82). In subgroup analysis grouped by adjustment status of prenatal complications, the pooled ORs were (1.29, 95% CI: 1.12–1.48) (adjustment status of prenatal complications & not-adjusted (1.24, 95% CI: 1.13–1.36). The pooled ORs were 1.15 (95% CI:0.92–1.43) for (EICS) & 1.47 (95% CI:1.33–1.62) for (EmCS). The meta-analysis concluded that CS and EmCS associated to increase the risk of postnatal depression, but no statistically significant association was found between ElCS and postnatal depression. |
| 64 | Yim | 2015 | Biological and psychosocial predictors of postpartum depression: systematic review and call for integration. | To identify biological and psychosocial factors associated with postnatal depression. | 199 | Not reported. | Total: 151,651 women in 1st postpartum year. Sample sizes ranged from 16 to 15,389. | Not reported. | USA, Australia, Canada, UK, Brazil. | Not reported. | Not reported. | Biological Risk Factors for Postnatal Depression: Hypothalamic-pituitary-adrenal dysregulation, inflammatory processes, & genetic & epigenetic vulnerabilities present stronger biological risk factors for postnatal depression. Strong Psychosocial factors for postnatal depression are severe stressful life events, chronic strains such as work demands & financial difficulties, relationship quality, & lack of support from partner & mother. |
| 65 | Zhang | 2019 | Maternal violence experiences and risk of postpartum depression: A meta-analysis of cohort studies. | To examine the association between maternal violence experiences and postnatal depression. | 32 | 32 cohort studies. | Total of 177,531 participants. Sample size ranged from 72 to 53,065. | Not reported. | 12 Middle & Low Income Countries 20 High Income Countries. | 4-7days to 12 months postpartum. | Most: EPDS, BDI, PHQ-9, BDI-FS, PDSS. | Overall, women who had experienced maternal violence was significantly related to a higher risk of postnatal depression (odds ratio [OR] = 2.04; 95% confidence interval [CI]: 1.72–2.41). This risk was significantly increased in women with a history of having experienced: sexual violence (OR = 1.56; 95%CI: 1.35–1.81), emotional violence (OR = 1.75; 95%CI: 1.61–1.89), physical violence (OR = 1.90; 95%CI: 1.36–2.67), domestic (OR = 2.05; 95%CI: 1.50–2.80) and childhood violence (OR = 1.59; 95%CI: 1.34–1.88). The association between history of maternal violence & higher risks of postnatal depression was observed even after subgroup and sensitivity analyses which indicates that the study findings are steady & reliable. |
| 66 | Paulson | 2020 | Intimate partner violence and perinatal post-traumatic stress and depression symptoms: a systematic review of findings in longitudinal studies. | To examine the associations between IPV and post-traumatic stress and perinatal depression. | 47 | 47 longitudinal studies | Sample size ranged from 72 to 13617 pregnant women. | Not reported. | East Asia & Pacific, Europe & Central Asia, Latin America & Caribbean, Middle East & North Africa, North America, South Asia, Sub-Saharan Africa | within pregnancy and 2 year postpartum. | Most: EPDS, CES-D | A strong association between IPV exposure and perinatal depression. |
| 67 | Madeghe | 2021 | Nutritional Deficiencies and Maternal Depression: Associations and Interventions in Lower and Middle-Income Countries: a Systematic Review of Literature | To examine the associations between nutritional deficiencies and perinatal depression and detect the role of diet in depression. | 25 | 13 cross-sectional studies, 8 cohort studies, 4 intervention studies | Sample size ranged from 27 to 7814 pregnant women. | Women of childbearing age, pregnant, and lactating women and there were no limitations placed on age. | Middle & Lower Income Countries | Pregnant and up to 1 year postpartum. | Most: EPDS, PSS, GHQ-12, SCID, DSM-IV, The Kessler Psychological Distress Scale (K-10), CES-D, MADRS, Xhosa version of the 10-item Edinburgh Postnatal Depression Scale, DASS-21-D | About 95% of these studies showed that there were positive associations between nutrition deficiencies, poor diet, and perinatal depression; 5% showed that there were no associations. |
| 68 | Ribamar | 2020 | Relationship between vitamin D deficiency and both gestational and postpartum depression | To examine the associations between vitamin D deficiency and perinatal depression | 8 | 1 prospective, 1 case-control, 1 cross-sectional, 3 cohort, 1 randomized clinical trial, 1 descriptive exploratory | Sample size ranged from 97 to 1,480 women, comprising 8,583 women | Not reported. | Iran, Netherlands, USA, Turkey, Australia, Denmark, China | Pregnant and postpartum. | Most: EPDS | 6 out of 8 articles found that there is a probable an association between vitamin D deficiency and both gestational and postnatal depression. |
| 69 | Desta | 2021 | Postpartum depression and its association with intimate partner violence and inadequate social support in Ethiopia: a systematic review and meta-analysis | To examine the pooled prevalence of postnatal depression in Ethiopia, as well as its relationship with intimate partner violence and a lack of social support | 13 | 12 studies were cross-sectional (8 studies were community-based cross-sectional studies (CBCS) and the remaining were facility-based cross-sectional studies (FBCS)), 1 cohort study | Total: 9,084 postnatal women. Sample size ranged from 122 to 3,147. | Not reported. | Ethiopia | Ranged from one month to 12 months postnatal | Most: EPDS, PHQ-9 | Postnatal depression was found in 21.55% of women (95% CI: 17.89, 25.94). Addis Abeba had the highest prevalence of postnatal depression (23.3%), while Harar had the lowest (13.11%). Meta-analysis results showed that women exposed to intimate partner violence had a 5.46 fold increased risk of postnatal depression (POR = 5.46, 95% CI: 3.94, 7.56, I2=38.8%) and women with insufficient social support had a 6.27 fold increased risk of postnatal depression (POR = 6.27, 95% CI: 4.83, 8.13, I2=0) compared to those with adequate social support. The meta-analysis also revealed a relationship between postnatal depression and marital dissatisfaction (POR = 2.6%; 95% CI: 1.48-4.65), past postnatal depression (POR = 2.03%; 95% CI: 1.72-2.4), and substance abuse (POR = 2.03%; 95% CI: 1.72-2.4). |
| 70 | Pereira | 2021 | Association between premenstrual dysphoric disorder and perinatal depression: a systematic review | To determine if premenstrual dysphoric disorder (PMDD) is a risk factor for perinatal depression. | 7 | 6x case-control, 1x cohort | Sample size ranged from 56 to 1308 | The study participants ranged in age from 18 to 44 years old, with the majority being married and living with their spouse, and a wide range of educational backgrounds. | Various countries such as Korea, the USA, England, Israel, Peru, Sweden and Switzerland | Ranged from 32 weeks of gestation to six months postnatal. | EPDS, GHQ-12, BDI, SCI | According to the studies, there is a positive and significant link between PMDD and the development of perinatal depression, particularly postnatal depression. Only one study has identified no significant link between PMDD and PPD at 4 weeks postnatally. |
| 71 | Yang | 2022 | Risk factors of perinatal depression in women: a systematic review  and meta-analysis | To examine the risk factors of perinatal depression in women. | 31 | cross-sectional studies, cohort studies, secondary data | Total: 79,043 women. Sample size ranged from 103 to 34,633 with a median of 564. | The study participants ranged in age from 18 to 44 years old, with the majority being married and living with their spouse, and a wide range of educational backgrounds. | There were 18 trials in underdeveloped nations and 13 trials in developed countries, with England having the most (5 trials). | Ranged from 32 weeks of gestation to six months postnatal. | EPDS, GHQ-12, ICD, HADS, SRQ-20 | The risk factors of perinatal depression included: educational level (P = 0.0001, odds ratio [OR]: 1.40, 95% CI: [1.18,1.67]), family economic status (P = 0.0001, OR: 1.69, 95%CI: [1.29,2.22]), history of mental illness (P < 0.00001, OR: 0.29, 95% CI: [0.18, 0.47]), intimate partner violence (P < 0.00001, OR: 0.24, 95% CI: [0.17,0.34]), perinatal smoking or drinking (P = 0.005, OR: 0.63; 95% CI [0.45, 0.87]; P = 0.008, OR: 0.43, 95% CI, [0.23 to 0.80]; respectively), and multiparity (P = 0.0003, OR: 0.74, 95% CI: [0.63, 0.87]) |
| 72 | Koric | 2021 | Polycystic ovary syndrome and postpartum depression: A systematic review and meta-analysis of observational studies | To examine the risk factors of perinatal depression in women with polycystic ovary syndrome (PCOS) and examine the moderators who are involved. | 6 | 3 cross-sectional studies, 3 cohort studies | Total: 934,922 women. Sample size ranged from 566 to 934,922. | The average age was 31.0 ± 5.5 years, and the average BMI was 25.8 ± 5.0 kg/m2. A total of 44,167 patients with PCOS (mean age=31.3 ± 4.7 years, mean BMI 28.6 ± 6.3 kg/m2) and 890,755 women without PCOS (mean age=31.0 ± 5.5 years, mean BMI 25.6 ± 4.8 kg/m2). | Not reported. | Not reported. | EPDS, CES-D, ICD | In six studies, 44,167 PCOS women had a higher risk of postnatal depression than 890,755 non-PCOS women (OR= 1.45, 95% CI= 1.18 to 1.79, p <0.001).The study found an OR of 1.59 (95% CI= 1.56 to 1.62, p <0.001) with decreased heterogeneity (I2= 45.3 %) after eliminating one research that overestimated PCOS prevalence. Lower proportion of preterm birth moderated higher ORs of PPD in women with PCOS (co-efficient -0.07, 95% CI= -0.1 to -0.04, p <0.001). After eliminating low-quality studies, the OR was 1.58 (95% CI= 1.56 to 1.59, p <0.001), with heterogeneity decreasing (I2= 14.0%). |
| 73 | Jin, X. | 2022 | Continuous supplementation of folic acid in pregnancy and the risk of perinatal depression: a meta-analysis. | To examine the risk of folic acid (FA) on perinatal depression. | 15 | 11 cross-sectional studies and cohort studies, 4 randomized clinical trial | Total: 26,275 women. Sample size ranged from 26 to 4044. | The average age was 31.0 ± 5.5 years, and the average BMI was 25.8 ± 5.0 kg/m2. A total of 44,167 patients with PCOS (mean age=31.3 ± 4.7 years, mean BMI 28.6 ± 6.3 kg/m2) and 890,755 women without PCOS (mean age=31.0 ± 5.5 years, mean BMI 25.6 ± 4.8 kg/m2). | Asia (n=11), Europe (n=2), Africa (n=1), and America (n=1). | Pregnant women or women within 12 months after giving the birth. | EPDS, CES-D, HAS, HAD, GHQ-20 | The overall odds ratio for the primary outcome of folic acid supplementation behaviour and risk of perinatal depression was 0.742 (95% CI: (0.647–0.852)), with a combined effect value of 0.84 (95% CI: (0.76, 0.93)) for studies in which an OR might be retrieved. Blood folate levels were shown to be negatively associated with depressed symptoms (standardised mean difference (SMD) =-0.127, 95% CI: (-0.183,-0.071)). There was no correlation between folic acid intervention and EPDS score. Continuous folic acid supplementation throughout pregnancy may minimise the occurrence of perinatal depression symptoms (R = 0.017, 95% CI: (0.014, 0.021)). |
| 74 | Schmidt | 2022 | Postpartum depression in maternal thyroidal changes. | To examine the potential of thyroid biomarkers as predictors for the development of postnatal depression. | 15 | 4 cross-sectional studies, 10 cohort studies, 1 case control study | Sample size ranged from ranged from 31 to 1075. | Not reported. | Not reported. | During pregnancy and in the 12 months postpartum period. | EPDS, HADS, HDRS, GHQ28, SCI, PSS, TAS, PQB | Prevalence estimates for postnatal depression in pregnant women with thyroid issues ranged from 8.3% to 36.0%. In a high-city survey for follow-up research, the cumulative incidence of self-reported depression from the initial episode in the first postnatal year was 6.3%. |
