# Supplementary material for: Prevalence and correlates of perinatal depression
Source: Soc Psychiatry Psychiatr Epidemiol. 2023 Jan 16;58(11):1581–90. doi: 10.1007/s00127-022-02386-9 (PMC9842219; doi:10.1007/s00127-022-02386-9)
Supplement: Supplementary file 4 — Supplementary file4 (DOCX 29 KB) [file 127_2022_2386_MOESM4_ESM.docx]

Table 3: The quality of the methodologies of all included systematic reviews, assessed using the AMSTAR tool

| **No.** | **Author** | **Was an 'a priori' design provided?** | **Was there duplicate study selection and data extraction?** | **Was a comprehensive literature search performed?** | **Was the status of publication (i.e. grey literature) used as an inclusion criterion?** | **Was a list of studies (included and excluded) provided?** | **Were the characteristics of the included studies provided?** | **Was the scientific quality of the included studies assessed and documented?** | **Was the scientific quality of the included studies used appropriately in formulating conclusions?** | **Were the methods used to combine the findings of studies appropriate?** | **Was the likelihood of publication bias assessed?** | **Was the conflict of interest stated?** | **Total** |
| --- | --- | --- | --- | --- | --- | --- | --- | --- | --- | --- | --- | --- | --- |
| 1 | Abajobir, A. A., 2016 | 1 | 0 | 0 | 1 | 1 | 1 | 1 | 0 | 1 | 0 | 1 | 7 |
| 2 | Aghajafari, F., 2018 | 1 | 1 | 1 | 0 | 1 | 1 | 1 | 1 | 1 | 0 | 1 | 9 |
| 3 | Alhasanat, D., 2015 | 0 | 0 | 1 | 1 | 1 | 1 | 0 | 0 | 1 | 0 | 0 | 5 |
| 4 | Alhasanat, D.,2017 | 0 | 0 | 1 | 1 | 1 | 1 | 0 | 0 | 1 | 0 | 0 | 5 |
| 5 | Alipour, Z., 2018 | 0 | 1 | 1 | 1 | 1 | 1 | 1 | 1 | 1 | 0 | 0 | 8 |
| 6 | Alvarez-Segura, M., 2014 | 0 | 0 | 1 | 1 | 1 | 1 | 1 | 1 | 1 | 0 | 0 | 7 |
| 7 | Alshikh Ahmad, H.,2021 | 0 | 1 | 1 | 0 | 1 | 1 | 1 | 1 | 1 | 1 | 1 | 9 |
| 8 | Anderson, F. M,2017 | 1 | 1 | 1 | 1 | 1 | 1 | 1 | 1 | 1 | 0 | 0 | 9 |
| 9 | Ahmed, A.,2019 | 0 | 1 | 1 | 1 | 1 | 1 | 1 | 1 | 1 | 1 | 1 | 10 |
| 10 | Atif, M.,,2021 | 1 | 1 | 1 | 0 | 1 | 1 | 1 | 1 | 1 | 1 | 0 | 9 |
| 11 | Ayano, G.,2019 | 1 | 1 | 1 | 1 | 1 | 1 | 1 | 1 | 1 | 1 | 0 | 10 |
| 12 | Azami, M.,2018 | 0 | 1 | 1 | 1 | 1 | 1 | 1 | 1 | 1 | 1 | 0 | 9 |
| 13 | Azami, M,2019 | 0 | 1 | 1 | 1 | 1 | 1 | 1 | 1 | 1 | 1 | 1 | 10 |
| 14 | Barakat, S.,2014 | 0 | 1 | 1 | 0 | 1 | 1 | 0 | 0 | 0 | 0 | 0 | 4 |
| 15 | Beck, C. T,2001 | 0 | 1 | 1 | 1 | 1 | 0 | 1 | 1 | 1 | 0 | 0 | 7 |
| 16 | Beck, C. T,1996 | 0 | 1 | 1 | 1 | 1 | 0 | 1 | 1 | 1 | 1 | 0 | 8 |
| 17 | Bell, A. F.,2016 | 0 | 0 | 1 | 1 | 1 | 1 | 1 | 1 | 1 | 0 | 1 | 8 |
| 18 | Bennett, H. A.,2004 | 0 | 1 | 1 | 1 | 1 | 1 | 1 | 1 | 1 | 1 | 0 | 9 |
| 19 | Beydoun, H. A.,2012 | 0 | 1 | 0 | 1 | 1 | 1 | 1 | 1 | 1 | 1 | 0 | 8 |
| 20 | Bhati, S.,2015 | 0 | 1 | 1 | 1 | 1 | 1 | 1 | 1 | 1 | 1 | 0 | 9 |
| 21 | Biaggi, A.,2016 | 0 | 1 | 1 | 1 | 1 | 0 | 0 | 0 | 1 | 0 | 0 | 5 |
| 22 | Brown, H. K.,2018 | 0 | 1 | 1 | 1 | 1 | 1 | 1 | 1 | 1 | 1 | 0 | 9 |
| 23 | Campbell-Jackson, L.,2014 | 0 | 1 | 1 | 1 | 1 | 1 | 0 | 0 | 1 | 0 | 0 | 6 |
| 24 | Carter, F. A.,2006 | 0 | 0 | 1 | 1 | 1 | 1 | 1 | 1 | 1 | 0 | 0 | 7 |
| 25 | Chapman, S. L. C.,2013 | 0 | 1 | 1 | 1 | 1 | 1 | 0 | 1 | 1 | 0 | 0 | 7 |
| 26 | Choi, K. W., 2016 | 0 | 0 | 1 | 1 | 1 | 1 | 0 | 1 | 1 | 0 | 0 | 6 |
| 27 | Chen, Q.,2022 | 0 | 1 | 1 | 0 | 1 | 1 | 1 | 1 | 1 | 1 | 1 | 9 |
| 28 | e Couto, T. C.,2015 | 0 | 0 | 1 | 1 | 1 | 0 | 0 | 0 | 1 | 0 | 0 | 4 |
| 29 | Corcoran, J.,2022 | 0 | 1 | 0 | 0 | 1 | 1 | 1 | 1 | 1 | 1 | 1 | 8 |
| 30 | Endomba, F. T,2021 | 0 | 1 | 1 | 0 | 1 | 1 | 1 | 1 | 1 | 0 | 1 | 8 |
| 31 | Dama, M.,2016 | 0 | 1 | 1 | 0 | 1 | 1 | 1 | 1 | 1 | 0 | 0 | 7 |
| 32 | Delahaije, D. H.,.2013 | 0 | 1 | 1 | 1 | 1 | 1 | 1 | 1 | 1 | 0 | 0 | 8 |
| 33 | Desta, M.,2021 | 0 | 1 | 1 | 0 | 1 | 1 | 1 | 1 | 1 | 1 | 1 | 9 |
| 34 | Dias, C. C., ,2015 | 0 | 1 | 1 | 1 | 1 | 1 | 1 | 1 | 1 | 0 | 0 | 8 |
| 35 | Elwood, J., ,2019 | 0 | 0 | 1 | 0 | 1 | 1 | 1 | 1 | 1 | 0 | 0 | 6 |
| 36 | Falah-Hassani,2017 | 1 | 1 | 1 | 0 | 1 | 1 | 0 | 0 | 1 | 0 | 1 | 7 |
| 37 | Falah-Hassani,2015 | 1 | 1 | 1 | 1 | 1 | 1 | 1 | 1 | 1 | 1 | 1 | 11 |
| 38 | Fellmeth, G.,,2017 | 1 | 1 | 1 | 0 | 1 | 1 | 1 | 1 | 1 | 0 | 1 | 9 |
| 39 | Fisher, J.,2012 | 0 | 1 | 1 | 1 | 1 | 1 | 1 | 1 | 1 | 1 | 0 | 9 |
| 40 | Gavin, N. I., ,2005 | 1 | 1 | 1 | 1 | 1 | 1 | 1 | 1 | 1 | 0 | 0 | 9 |
| 41 | Gelaye, B.,2016 | 0 | 0 | 1 | 0 | 1 | 1 | 0 | 1 | 1 | 1 | 1 | 7 |
| 42 | Getinet, W.,2018 | 1 | 1 | 1 | 1 | 1 | 1 | 1 | 1 | 1 | 1 | 1 | 11 |
| 43 | Hahn-Holbrook, J.,2018 | 0 | 0 | 1 | 1 | 1 | 0 | 0 | 1 | 1 | 0 | 1 | 6 |
| 44 | Halbreich, U.,2006 | 0 | 0 | 1 | 1 | 1 | 1 | 0 | 0 | 1 | 0 | 0 | 5 |
| 45 | Halim, N.,2018 | 0 | 1 | 1 | 1 | 1 | 1 | 1 | 1 | 1 | 0 | 1 | 9 |
| 46 | Han, A.,2014 | 0 | 0 | 1 | 0 | 1 | 1 | 0 | 0 | 1 | 0 | 1 | 5 |
| 47 | Howard, L. M.,2013 | 1 | 1 | 1 | 1 | 1 | 1 | 1 | 1 | 1 | 0 | 0 | 9 |
| 48 | Hutchens, B. F.2017 | 0 | 1 | 1 | 0 | 1 | 1 | 1 | 1 | 1 | 0 | 1 | 8 |
| 49 | Hymas, R.,2018 | 1 | 1 | 1 | 1 | 1 | 1 | 1 | 1 | 1 | 0 | 0 | 9 |
| 50 | James-Hawkins, L.2019 | 1 | 1 | 1 | 1 | 1 | 1 | 1 | 1 | 1 | 0 | 1 | 10 |
| 51 | Jha, S.,2018 | 0 | 0 | 1 | 1 | 1 | 1 | 1 | 1 | 1 | 0 | 1 | 8 |
| 52 | Jin, X.,2022 | 1 | 1 | 1 | 0 | 1 | 1 | 1 | 1 | 1 | 1 | 1 | 10 |
| 53 | Jones, E.,2013 | 0 | 0 | 1 | 1 | 1 | 1 | 1 | 1 | 1 | 0 | 0 | 7 |
| 54 | Karaçam, Z., 2018 | 1 | 1 | 1 | 1 | 1 | 1 | 1 | 1 | 1 | 0 | 0 | 9 |
| 55 | Klainin, 2009 | 0 | 0 | 1 | 1 | 1 | 1 | 0 | 0 | 1 | 0 | 1 | 6 |
| 56 | Koric, A.,2021 | 1 | 1 | 1 | 0 | 1 | 1 | 1 | 1 | 1 | 1 | 1 | 10 |
| 57 | Klaman, S. L., 2016 | 0 | 0 | 1 | 1 | 1 | 1 | 1 | 1 | 1 | 0 | 1 | 8 |
| 58 | Lambert, M.,2019 | 0 | 0 | 1 | 0 | 1 | 1 | 0 | 0 | 1 | 0 | 1 | 5 |
| 59 | Lancaster, C. A.,2010 | 0 | 1 | 1 | 1 | 1 | 1 | 1 | 1 | 1 | 0 | 0 | 8 |
| 60 | Lara-Cinisomo, S.,2016 | 0 | 0 | 1 | 1 | 1 | 1 | 0 | 0 | 1 | 0 | 0 | 5 |
| 61 | Lara-Cinisomo, S., 2018 | 0 | 1 | 1 | 1 | 1 | 1 | 1 | 1 | 1 | 0 | 1 | 9 |
| 62 | Lawson, A.,2015 | 0 | 1 | 1 | 1 | 1 | 1 | 1 | 1 | 1 | 0 | 1 | 9 |
| 63 | Liu, X.,2021 | 1 | 1 | 1 | 0 | 1 | 1 | 1 | 1 | 1 | 1 | 1 | 10 |
| 64 | Madeghe, B. A.,2021 | 0 | 1 | 1 | 0 | 1 | 1 | 1 | 1 | 1 | 0 | 0 | 7 |
| 65 | Mahendran, R.,2019 | 1 | 1 | 1 | 1 | 1 | 1 | 1 | 1 | 1 | 1 | 1 | 11 |
| 66 | Lobato, G.,2011 | o | o | 1 | 0 | 1 | 1 | 0 | 0 | 1 | 0 | 0 | 4 |
| 67 | Khatri, G. K., 2019 | 1 | 0 | 1 | 1 | 1 | 1 | 1 | 1 | 1 | 0 | 1 | 9 |
| 68 | Mersha, A. G.,2018 | 0 | 1 | 1 | 1 | 1 | 1 | 1 | 1 | 1 | 1 | 1 | 10 |
| 69 | Moameri, H.,2019 | 0 | 1 | 1 | 1 | 1 | 1 | 1 | 1 | 1 | 1 | 1 | 10 |
| 70 | Molyneaux, E.,2014 | 1 | 1 | 1 | 1 | 1 | 1 | 1 | 1 | 1 | 1 | 0 | 10 |
| 71 | Moura, D.,2016 | 0 | 0 | 1 | 1 | 1 | 1 | 1 | 1 | 1 | 0 | 1 | 8 |
| 72 | Mu, T. Y., 2019 | 0 | 1 | 1 | 0 | 1 | 1 | 1 | 1 | 1 | 1 | 1 | 9 |
| 73 | Mukherjee, S.,2014 | 1 | 1 | 1 | 1 | 1 | 1 | 1 | 1 | 1 | 1 | 0 | 10 |
| 74 | Mukherjee, S.,2016 | 1 | 0 | 1 | 1 | 1 | 1 | 1 | 1 | 1 | 0 | 0 | 8 |
| 75 | Nilaweera, I., 2014 | 0 | 0 | 1 | 1 | 1 | 1 | 1 | 1 | 1 | 0 | 1 | 8 |
| 76 | Olieman, R. M.,2017 | 1 | 1 | 1 | 1 | 1 | 1 | 1 | 1 | 1 | 0 | 0 | 9 |
| 77 | Orta, O. R.,2018 | 0 | 0 | 1 | 1 | 1 | 1 | 0 | 0 | 1 | 0 | 1 | 6 |
| 78 | Özcan, N.2017 | 0 | 0 | 1 | 1 | 1 | 1 | 1 | 1 | 1 | 0 | 0 | 7 |
| 79 | Paulson, J. L.,2020 | 0 | 1 | 1 | 0 | 1 | 1 | 0 | 1 | 1 | 1 | 1 | 8 |
| 80 | Pereira, D., 2021 | 0 | 1 | 1 | 1 | 1 | 1 | 0 | 0 | 1 | 1 | 1 | 8 |
| 81 | Petzoldt, J.2018 | 0 | 0 | 1 | 1 | 1 | 1 | 1 | 1 | 1 | 0 | 1 | 8 |
| 82 | Ribamar, A.,2020 | 0 | 0 | 1 | 0 | 1 | 1 | 0 | 1 | 1 | 0 | 0 | 5 |
| 83 | Razurel, C.,2013 | 0 | 1 | 1 | 1 | 1 | 1 | 0 | 0 | 1 | 0 | 0 | 6 |
| 84 | Recto, P., 2017 | 0 | 0 | 1 | 1 | 1 | 1 | 0 | 0 | 1 | 0 | 1 | 6 |
| 85 | Ren, J. H., 2014 | 0 | 0 | 1 | 1 | 1 | 1 | 1 | 1 | 1 | 0 | 0 | 7 |
| 86 | Ross, L. E., 2009 | 0 | 0 | 1 | 1 | 1 | 1 | 0 | 0 | 1 | 0 | 1 | 6 |
| 87 | Ross, L. E.,2010 | 0 | 1 | 1 | 1 | 1 | 1 | 1 | 1 | 1 | 0 | 0 | 8 |
| 88 | Sawyer, A.,2010 | 0 | 0 | 1 | 1 | 1 | 1 | 1 | 1 | 1 | 0 | 1 | 8 |
| 89 | Schmied, V.,2013 | 0 | 0 | 1 | 1 | 1 | 1 | 1 | 1 | 1 | 0 | 0 | 7 |
| 90 | Serati, M.,2016 | 0 | 0 | 1 | 0 | 1 | 1 | 0 | 0 | 1 | 0 | 0 | 4 |
| 91 | Shamblaw, A. L.,2019 | 0 | 1 | 1 | 0 | 1 | 1 | 1 | 1 | 1 | 1 | 1 | 9 |
| 92 | Shorey, S.,2018 | 1 | 1 | 1 | 1 | 1 | 1 | 1 | 1 | 1 | 0 | 1 | 10 |
| 93 | Shorey, S. Y., 2021 | 0 | 1 | 1 | 0 | 1 | 1 | 1 | 1 | 0 | 1 | 1 | 8 |
| 94 | Schmidt, P. M. D. S., 2022 | 0 | 1 | 1 | 1 | 1 | 0 | 0 | 0 | 1 | 0 | 1 | 6 |
| 95 | Silveira, M. L., 2015 | 0 | 0 | 1 | 1 | 1 | 1 | 0 | 0 | 1 | 0 | 1 | 6 |
| 96 | Sowa, N. A., 2015 | 0 | 1 | 1 | 1 | 1 | 1 | 1 | 1 | 1 | 0 | 1 | 9 |
| 97 | Sparling, T. M.,2017 | 0 | 1 | 1 | 0 | 1 | 1 | 1 | 1 | 1 | 0 | 1 | 8 |
| 98 | Sparling, T. M., 2017 | 0 | 0 | 1 | 1 | 1 | 1 | 1 | 1 | 1 | 0 | 0 | 7 |
| 99 | Safi‐Keykaleh, M.,2022 | 1 | 1 | 1 | 0 | 1 | 1 | 1 | 1 | 1 | 1 | 1 | 10 |
| 100 | Steinig, J.,2017 | 0 | 1 | 1 | 1 | 1 | 1 | 1 | 1 | 1 | 0 | 1 | 9 |
| 101 | Suzuki, D., 2018 | 0 | 1 | 1 | 1 | 1 | 1 | 1 | 1 | 1 | 0 | 0 | 8 |
| 102 | Takegata, M.,2017 | 0 | 0 | 1 | 1 | 1 | 1 | 0 | 0 | 1 | 0 | 1 | 6 |
| 103 | Teychenne, M.,2013 | 0 | 0 | 1 | 1 | 1 | 1 | 1 | 1 | 1 | 0 | 0 | 7 |
| 104 | Ti, A., 2019 | 1 | 1 | 1 | 0 | 1 | 1 | 1 | 1 | 1 | 0 | 0 | 8 |
| 105 | Trujillo, J., 2018 | 1 | 1 | 1 | 0 | 1 | 1 | 1 | 1 | 1 | 0 | 0 | 8 |
| 106 | Underwood, L.,2016 | 0 | 0 | 1 | 1 | 1 | 1 | 0 | 0 | 1 | 0 | 1 | 6 |
| 107 | Upadhyay, R. P., 2017 | 0 | 1 | 1 | 1 | 1 | 1 | 1 | 1 | 1 | 1 | 1 | 10 |
| 108 | Veisani, Y.,2012 | 0 | 0 | 1 | 0 | 1 | 1 | 0 | 0 | 1 | 0 | 0 | 4 |
| 109 | Veisani, Y., 2013 | 0 | 1 | 1 | 1 | 1 | 1 | 0 | 0 | 1 | 1 | 0 | 7 |
| 110 | Vigod, S. N., 2010 | 0 | 1 | 1 | 1 | 1 | 1 | 1 | 1 | 1 | 0 | 1 | 9 |
| 111 | Villegas, L., 2011 | 0 | 1 | 1 | 1 | 1 | 1 | 0 | 0 | 1 | 0 | 0 | 6 |
| 112 | Vliegen, N.,2014 | 0 | 1 | 1 | 1 | 1 | 1 | 0 | 0 | 1 | 0 | 1 | 7 |
| 113 | Wang, J.,2018 | 0 | 1 | 1 | 1 | 1 | 1 | 1 | 1 | 1 | 1 | 1 | 10 |
| 114 | Warfa, N.,2014 | 0 | 0 | 1 | 0 | 1 | 1 | 1 | 1 | 1 | 0 | 0 | 6 |
| 115 | Wassef, A.,2019 | 0 | 1 | 1 | 1 | 1 | 1 | 0 | 0 | 1 | 0 | 1 | 7 |
| 116 | Wittkowski, A.,2014 | 0 | 1 | 1 | 1 | 1 | 1 | 1 | 1 | 1 | 0 | 1 | 9 |
| 117 | Wojcicki, J. M.,2011 | 0 | 0 | 1 | 0 | 1 | 1 | 0 | 0 | 1 | 0 | 1 | 5 |
| 118 | Wong, J., 2009 | 0 | 0 | 1 | 1 | 1 | 1 | 0 | 0 | 1 | 0 | 1 | 6 |
| 119 | Woody, C. A.,2017 | 0 | 0 | 1 | 1 | 1 | 0 | 0 | 0 | 1 | 1 | 1 | 6 |
| 120 | Wosu, A. C., 2015 | 0 | 0 | 1 | 1 | 1 | 1 | 1 | 1 | 1 | 0 | 1 | 8 |
| 121 | Wu, Q., 2012 | 0 | 1 | 1 | 1 | 1 | 1 | 1 | 1 | 1 | 1 | 1 | 10 |
| 122 | Xiao, R. S., 2014 | 0 | 1 | 1 | 1 | 1 | 1 | 1 | 1 | 1 | 0 | 0 | 8 |
| 123 | Xu, H., 2017 | 0 | 1 | 1 | 0 | 1 | 1 | 1 | 1 | 1 | 1 | 1 | 9 |
| 124 | Yim, I. S., 2015 | 0 | 0 | 1 | 1 | 1 | 1 | 0 | 0 | 1 | 0 | 0 | 5 |
| 125 | Yin, X.,2021 | 0 | 1 | 1 | 0 | 1 | 1 | 1 | 1 | 1 | 1 | 1 | 9 |
| 126 | Yang, K.,2022 | 0 | 0 | 1 | 0 | 1 | 1 | 1 | 1 | 1 | 1 | 1 | 8 |
| 127 | Zegeye, A.,2018 | 0 | 1 | 1 | 1 | 1 | 1 | 1 | 1 | 1 | 1 | 0 | 9 |
| 128 | Zhang, S.,2019 | 0 | 1 | 1 | 0 | 1 | 1 | 1 | 1 | 1 | 1 | 1 | 9 |
