# Supplementary material for: Prevalence and correlates of perinatal depression
Source: Soc Psychiatry Psychiatr Epidemiol. 2023 Jan 16;58(11):1581–90. doi: 10.1007/s00127-022-02386-9 (PMC9842219; doi:10.1007/s00127-022-02386-9)
Supplement: Supplementary file 5 — Supplementary file5 (DOCX 19 KB) [file 127_2022_2386_MOESM5_ESM.docx]

Table of Abbreviations for table 1 and table 2

| **Table of Abbreviations** | **Full terms** |
| --- | --- |
| ADS-K | Allgemeine Depressions Skala (German version of CESD) |
| AKUADS | Aga Khan University Anxiety and Depression Scale |
| BAI | Beck Anxiety Inventory |
| BDI | Beck Depression Inventory |
| BDI-II | Beck Depression Inventory-Second Edition |
| BDI-FS | Beck Depression Inventory Fast Screen |
| BDS | Beck Depression Scale |
| BHQ | Brief Patient Health Questionnaire |
| BSSQ | Brief Symptom Severity Questionnaire |
| BPDS | Bromley Postnatal Depression Scale |
| BSI | Brief Symptom Inventory (BSI). |
| CD | Clinical Diagnosis |
| CDRS | Children’s Depression Rating Scale |
| CDRS-R | Children's Depression Rating Scale-Revised |
| CES-D | Centre for Epidemiological Studies Depression Scale. |
| CES-DMS | Centre for Epidemiologic Studies Depressed Mood Scale. |
| CHQ-12 | Chinese Health Questionnaire |
| CIDI | Composite International Diagnostic Interview |
| CIDI-D | Composite International Diagnostic Interview-Depression module |
| CIS | Clinical Interview Schedule |
| CIDI-SF | Composite International Diagnostic Interview-Short Form |
| CIS-R | Clinical Interview Schedule-Revised |
| DIS | Diagnostic Interview Schedules |
| CRQS | Clinician-Rated Questionnaire Scales. |
| DACL | Depression Adjective Checklist |
| DASS-21 | Depression Anxiety Stress Scales |
| DASS-42 | Depression Anxiety Stress Scale-42 item |
| DIGS | Diagnostic Criteria for Genetic Studies. |
| DDI | Depression Detailed Inventory |
| DIS-IV | Diagnostic Interview Schedule |
| DPI | Diagnostic Psychiatric Interview. |
| DSM-IV | Diagnostic and Statistical Manual |
| DSM IV-TR | Diagnostic and Statistical Manual of Mental Disorders Text Revision |
| DSS | Daily Scoring System. |
| DSSI | Delusion-States-Symptoms Inventory Anxiety and Depression Scale |
| DSSI-7 | 7-item depression subscale of DSSI. |
| EPDS | Edinburgh Postpartum Depression Scale. |
| FDA | Formal Diagnostic Assessment |
| GHQ-12 | General Health Questionnaire -12 item |
| GHQ-28 | General Health Questionnaire-28 item |
| GHQ-30 | General Health Questionnaire-30 item. |
| GHQ-20 | General Health Questionnaire-20 item |
| GADS | Goldberg Anxiety and Depression Scale |
| HADS | Hospital Anxiety and Depression Scale |
| HADS-D | Hospital Anxiety and Depression Scale-Depression Scale |
| HAS | Hamilton Anxiety Scale |
| HDRS | Hamilton Depression Rating Scale. |
| HRSD-17 | Hamilton Rating Scale for Depression for a Clinical Syndrome using Structured Diagnostic Interviews |
| HSCL-8, HSCL-15 | Subsets of the Hopkins Symptoms Checklist |
| HSCL | Hopkins Symptom Checklist |
| HSCL-25 | Hopkins Symptom Checklist-25. |
| IACLIDE | Inventory of the Clinical Evaluation of Depressions |
| ICD | The International Classification of Diseases |
| ICD-9-CM | Ninth Edition of the International Classification of Diseases' Clinical Modification |
| ICD-10. | Tenth Edition of the International Classification of Diseases (ICD-10). |
| IC-10 PCVC | International Classification of Diseases10 Primary Care Version Criteria. |
| IDD | Inventory to Diagnose Depression. |
| IDQ | Investigator-Developed Questionnaire |
| IDS | Inventory for Depression Symptomatology |
| K6 | Kessler 6-item Psychological Distress Scale |
| K-10 | Kessler Psychological Distress Scale |
| KGB | Kennerley and Gath Maternity Blues Assessment Scale |
| MADRS | Montgomery-Asberg Depression Scale |
| MINI | Mini International Neuropsychiatric Interview. |
| MMI | Modified Malaise Inventory score |
| OGTT | Oral Tolerance Glucose Test |
| PCEMD | Primary Care Evaluation of Mental Disorders |
| PDQ | Pitt Depression Questionnaire |
| PDRS | Postpartum Depression Risk Schedule |
| PDS | Postpartum Depression Scale. |
| PDSS | Postpartum Depression Screening Scale |
| PDSS-S | Postpartum Depression Screening Scale- Spanish |
| PDSS-SF | Postpartum Depression Screening Scale-Short Form |
| PEDQ | Postpartum Emotional Disorders Questionnaire. |
| PHQ-2 | Patient Health Questionnaire |
| PHQ-9 | Patient Health Questionnaire Depression Module. |
| POMS-D | Profile of Mood States’ Subscale for Depression-Dejection. |
| PRAQ | Pregnancy Related Anxiety Questionnaire. |
| PRQ | Pregnancy Risk Questionnaire |
| PRIME‐MD | Primary Care Evaluation of Mental Disorders. |
| PSRS | Pregnancy Stress Rating Scale |
| PSS-SR | PTSD Symptom Scale – Self‐Report |
| PTSD-SS-SRQ | Post-Traumatic Stress Disorder Symptom Scale Self-Report-Questionnaire. |
| *PQB* | Podromal Questionnaire Brief version |
| RDC | Research Diagnostic Criteria |
| SADS | Schedule for Affective Disorders and Schizophrenia |
| SC-90 | Symptom Checklist 90 |
| SCAN | Schedule for Clinical Assessment in Neuropsychiatry |
| SCI | Structured Clinical Interviews |
| SCI/CD | Structured Clinical Interview / Clinical Diagnosis. |
| SCL-A | Symptom-Checklist -Anxiety (Hopkins) |
| SCL-8 | Symptom-Checklist-8 (Hopkins) |
| SCL-90 | Symptom Checklist-90 Depression Subscale |
| SCL-90-R | Symptom Distress Check List |
| SCL-92 | Symptoms Checklist-92 . |
| SDS | Self-Rating Depression Scale |
| SDAS | Spanier Dyadic Adjustment Scale-7 |
| SF36 | Short Form 36 Mental Health Subscale (revised to conﬁrm the diagnosis of postnatal depression.) |
| SRDS | Self-Rating Scale for Depression. |
| SI | Structured Interviews |
| SIGH-ADS29 | Structured Interview Guide for the Hamilton Depression Rating Scale-Seasonal Affective Disorder |
| SI-ICD 10 | Structured Interview for ICD-10 |
| SPI | Standard Psychiatric Interview |
| S-R | Self-Report |
| SRDS | Self-Rating Scale for Depression. |
| SRQ | Self-Reporting Questionnaire |
| SRQ-20 | WHO Self-Reporting Questionnaire |
| SRQ-25 | Self-reporting Questionnaire-25 |
| SSI | Semi-Structured Interviews |
| SSQ | Shona Symptom Questionnaire |
|  |  |
| SSDS | Siddiqui-Shah Depression Scale |
| TAS | Toronto Alexithymia Scale |
